# Supplementary material for: Chemical manipulation of an activation/inhibition switch in the nuclear receptor PXR
Source: Nat Commun. 2024 May 14;15:4054. doi: 10.1038/s41467-024-48472-1 (PMC11094003; doi:10.1038/s41467-024-48472-1)
Supplement: Supplementary file 1 — Supplementary Information [file 41467_2024_48472_MOESM1_ESM.pdf]

Chemical manipulation of an activation/inhibition switch in the nuclear receptor PXR

Efren Garcia-Maldonado[1,†], Andrew D. Huber[1,†,*], Sergio C. Chai[1,†], Stanley Nithianantham[1,†], Yongtao Li[1,†], Jing Wu[1], Shyaron Poudel[1], Darcie J. Miller[2], Jayaraman Seetharaman[2], Taosheng Chen[1,*]

[1]Department of Chemical Biology and Therapeutics, St. Jude Children's Research Hospital, 262 Danny Thomas Place, Memphis, TN, 38105, USA.

[2]Department of Structural Biology, St. Jude Children's Research Hospital, 262 Danny Thomas Place, Memphis, TN, 38105, USA.

[†]These authors contributed equally to this work

*Corresponding authors; address: 262 Danny Thomas Place, MS 1000, Memphis, TN 38105; email address: andrew.huber@stjude.org; phone: (901) 595-6030; email address: taosheng.chen@stjude.org; phone: (901) 595-5937

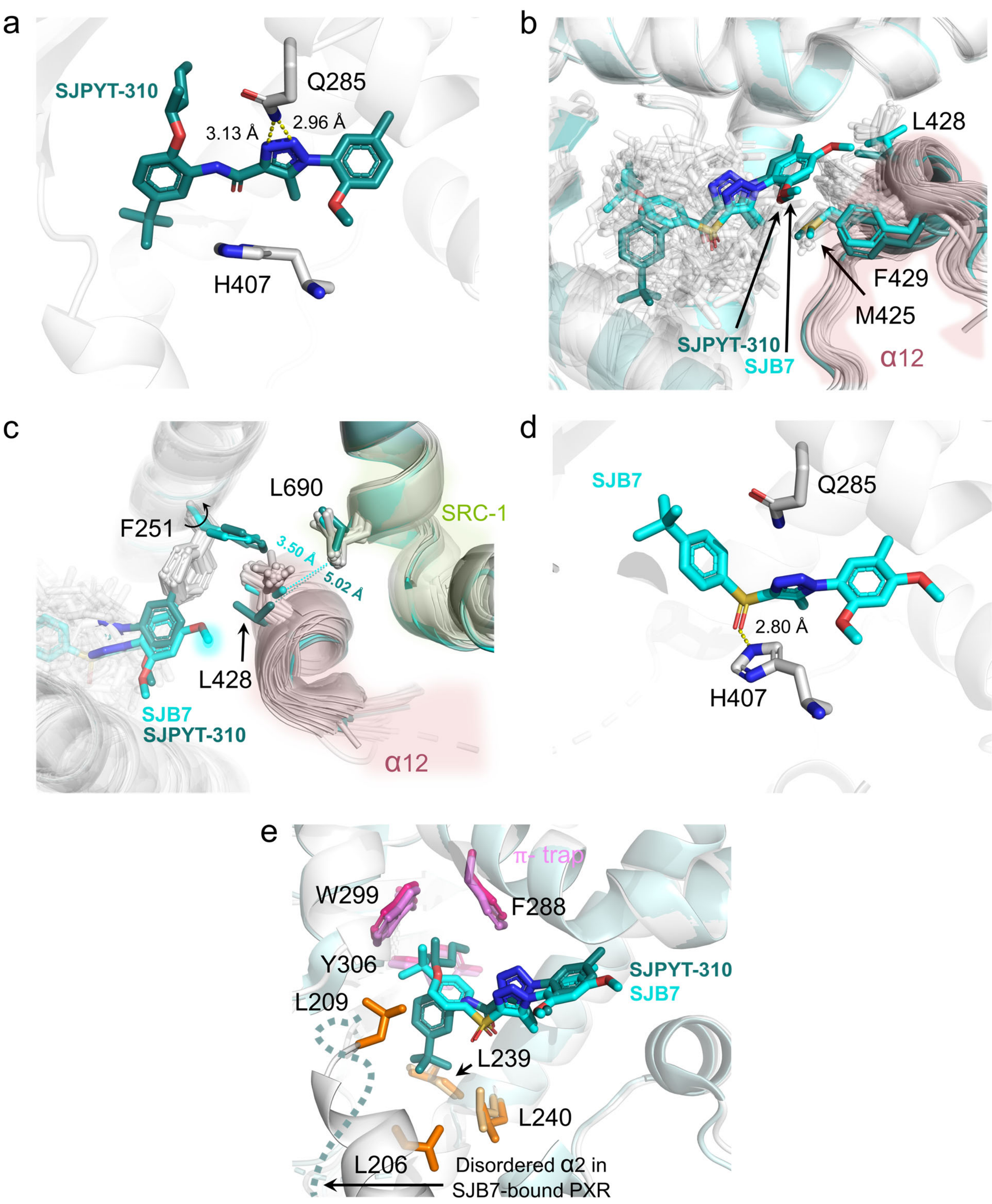

Supplementary Figure 1. SJPYT-310 has a unique PXR LBD binding mode. (a) The triazole ring of SJPYT-310 interacts with Q285 through a pair of N-H···N hydrogen bonds (dashed yellow lines). **(b)** Comparison of SJPYT-310-bound PXR LBD structure (deep teal) with all other ligand-bound PXR LBD structures (gray). α12 residues M425, L428, and F429 and all ligands are shown as stick representations. SJB7 is shown as cyan sticks. **(c)** SJB7-bound PXR LBD maintains contact between L428 and SRC-1 residue L690 compared to SJPYT-310-bound PXR LBD. Like SJPYT-310, SJB7 reorients F251 due to steric hindrance. **(d)** The sulfonyl moiety of SJB7 forms a hydrogen bond (dashed yellow line) with H407. **(e)** SJPYT-310 (deep teal) interacts with the π-trap (violet) and leucine cage (orange), whereas SJB7 only binds to the π-trap (pink) and not the leucine cage (light orange). α2 (containing L206 and L209) is disordered and unobserved in the SJB7-bound PXR LBD structure (dotted line).

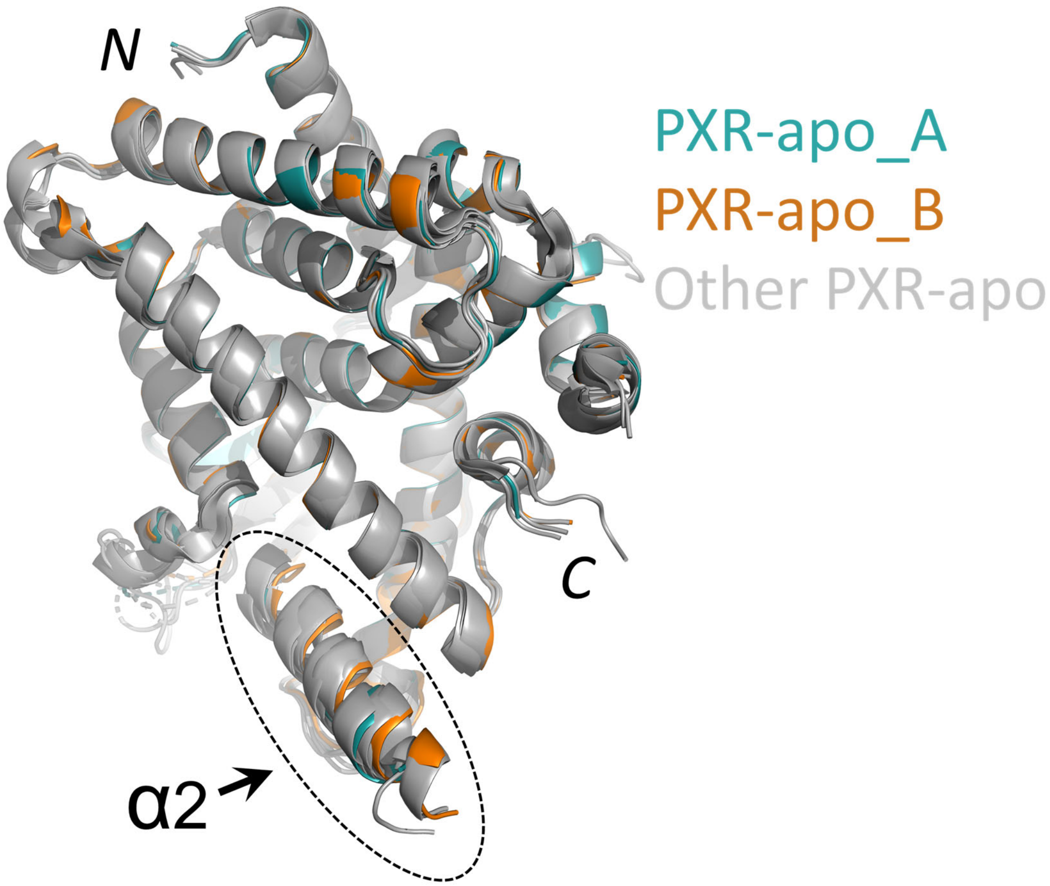

Supplementary Figure 2. **Comparative analysis of apo PXR LBD structures**. Superimposition of apo PXR LBD (PXR-apo) (chain A, cyan; chain B, orange) onto previously reported apo PXR LBD structures (Other PXR-apo, gray).

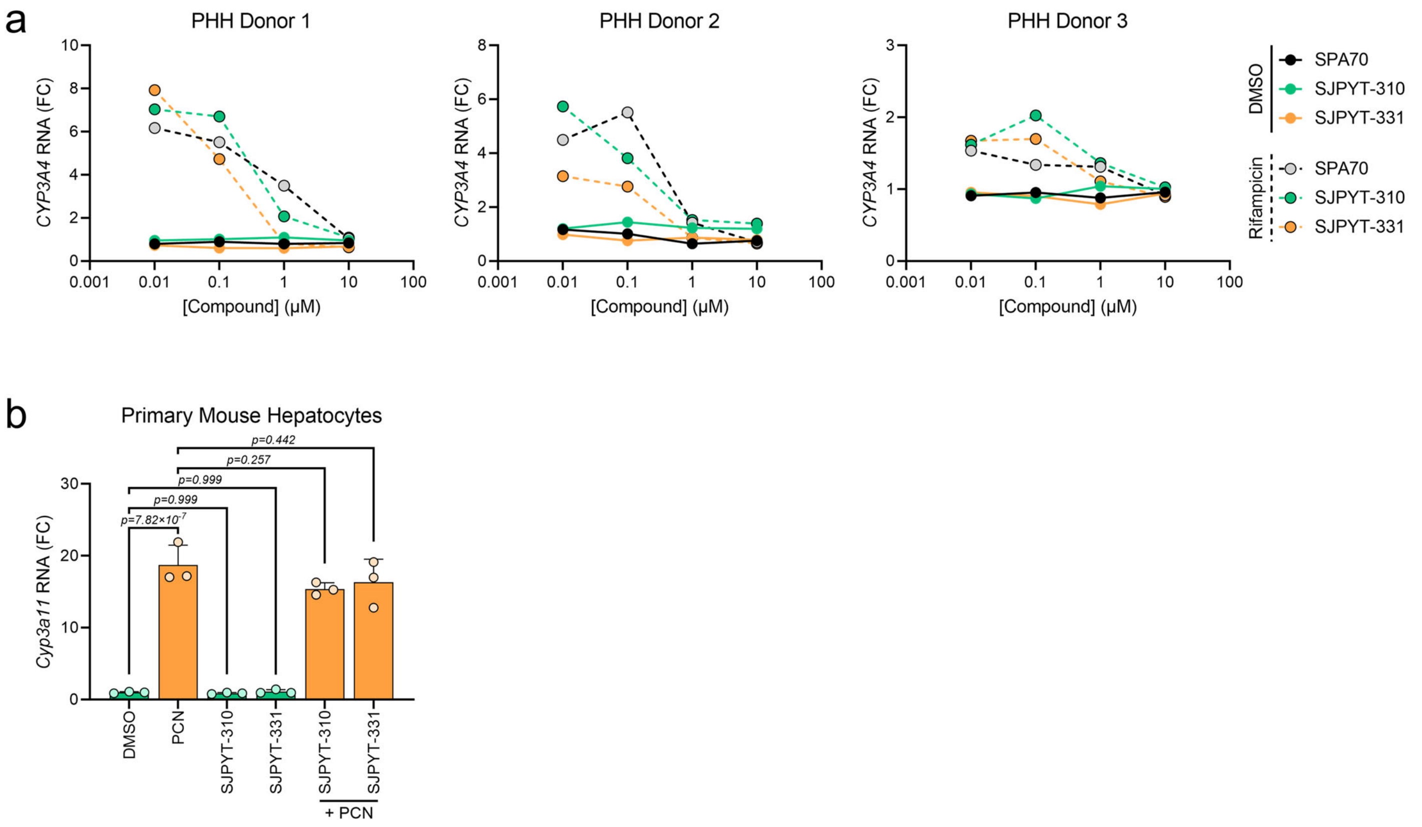

Supplementary Figure 3. **Antagonists block PXR activation in primary human hepatocytes (PHH)**. **(a)** PHH were treated with DMSO or compounds for 24 h, and RNA was extracted and subjected to RT-qPCR. *CYP3A4* RNA level was normalized to *18S* RNA for each sample, and data were then normalized as fold change (FC) relative to the DMSO control. **(b)** Primary mouse hepatocytes were treated with DMSO, 3 µM PCN, 10 µM SJPYT-310, or 10 µM SJPYT-331 for 48 h. RNA was extracted and subjected to RT-qPCR, *Cyp3a11* RNA level was normalized to *18S* RNA for each sample, and data were normalized as FC relative to the DMSO control. Data are presented as mean values +/- standard deviation (SD), and each point represents a biological replicate (n = 3 for each treatment). Significance was assessed with one-way ANOVA followed by Dunnett's test for each treatment compared to either DMSO or PCN. Source data are provided as a Source Data file.

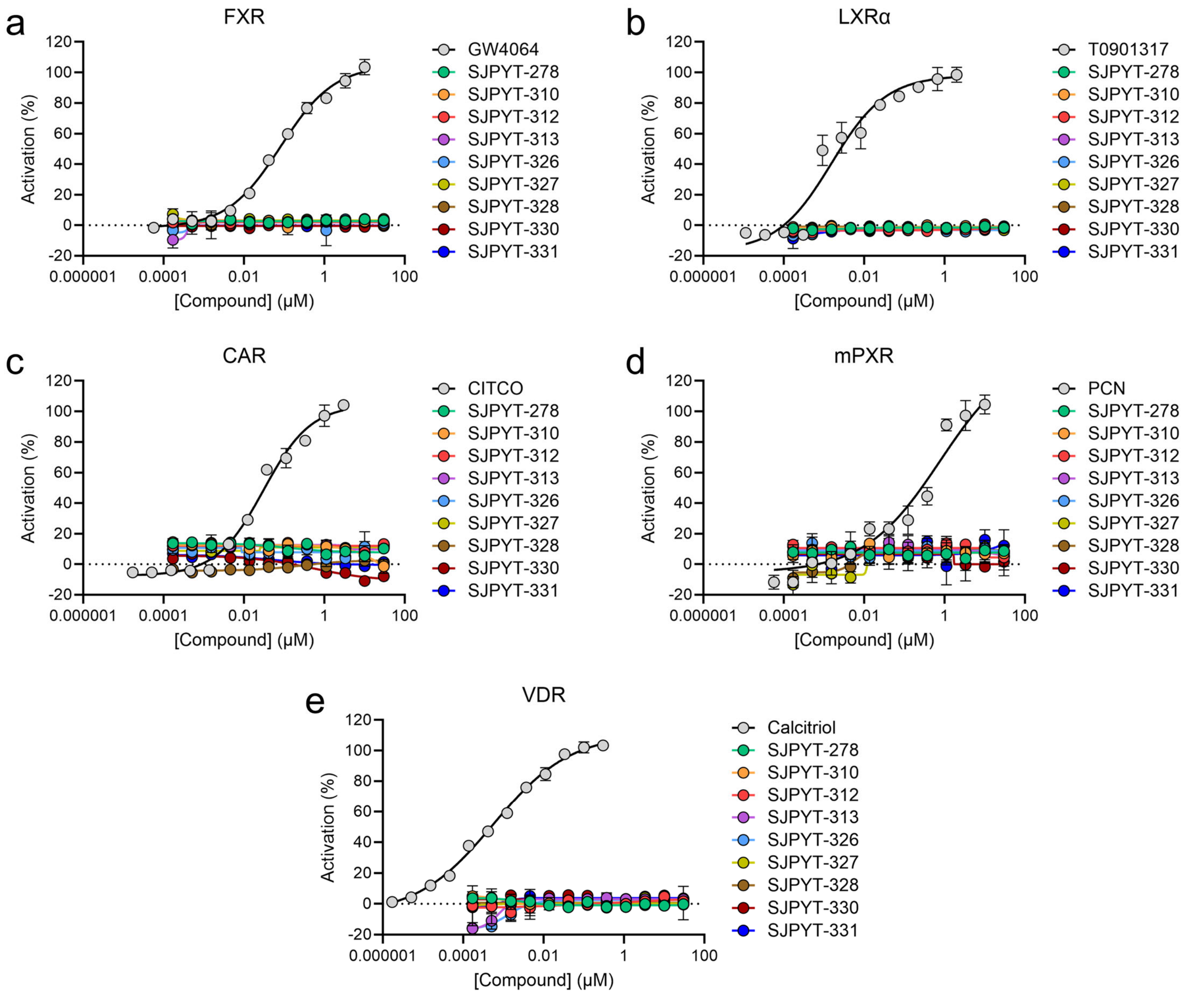

Supplementary Figure 4. PXR modulators do not activate a panel of other NRs. Nuclear receptor activation assays were conducted for **(a)** farnesoid X receptor (FXR), **(b)** liver X receptor α (LXRα), **(c)** constitutive androstane receptor (CAR), **(d)** mouse PXR (mPXR), or **(e)** vitamin D receptor (VDR). Known agonists for each receptor are included as reference. Data were normalized from 0% to 100% using DMSO (0%) and the respective nuclear receptor agonist (100%). Data were derived from n=3 independent experiments and are presented as mean values +/- SD. Source data are provided as a Source Data file.

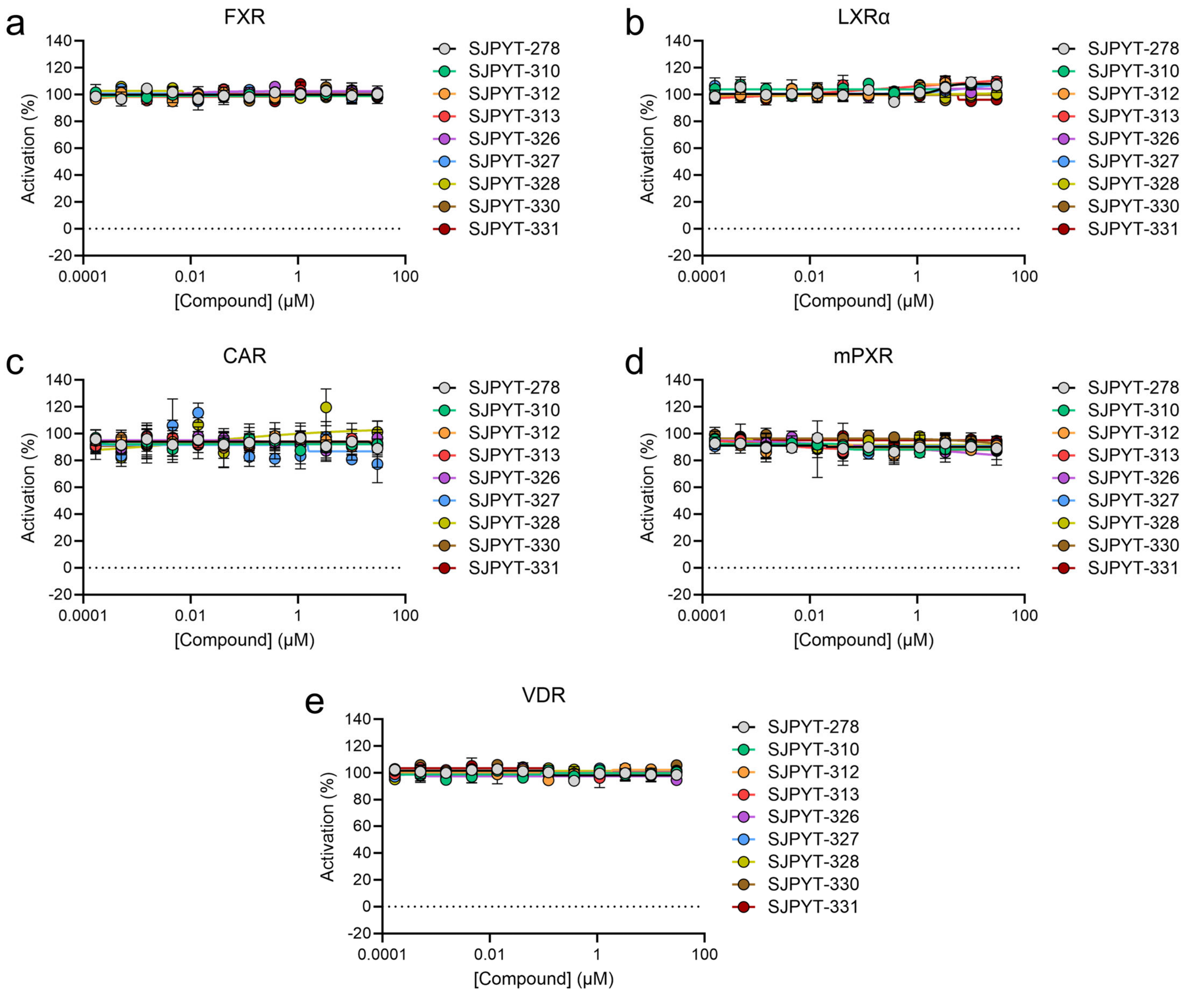

Supplementary Figure 5. PXR modulators do not block activation of a panel of other NRs. Nuclear receptor antagonist assays were conducted for **(a)** FXR, **(b)** LXRα, **(c)** CAR, **(d)** mPXR, or **(e)** VDR. Data were normalized from 0% to 100% using DMSO (0%) and the respective nuclear receptor agonist (100%). Data were derived from n=3 independent experiments and are presented as mean values +/- SD. Source data are provided as a Source Data file.

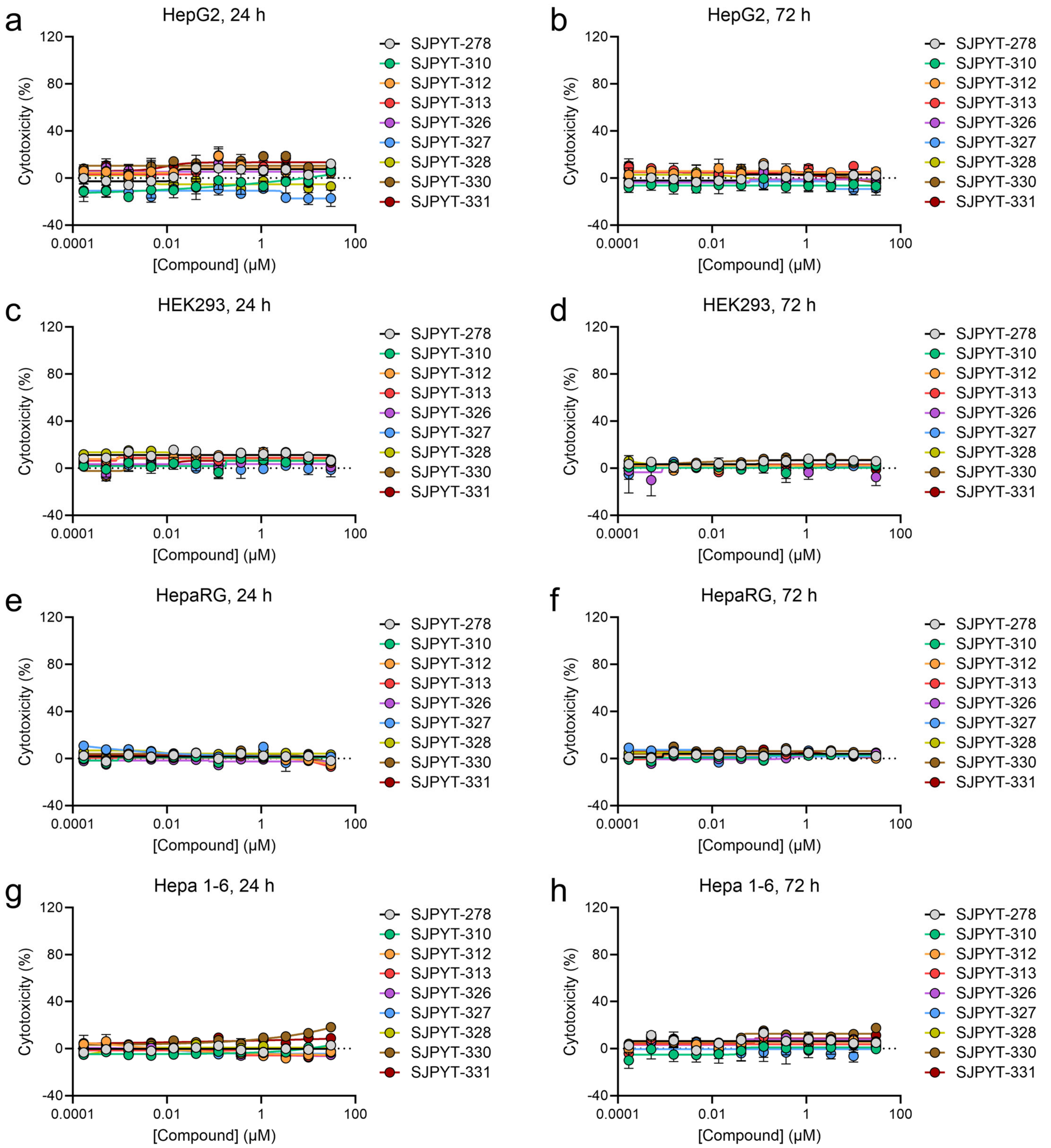

Supplementary Figure 6. PXR modulators are not cytotoxic. Cells were treated with compounds for 24 or 72 h and assessed for viability with the CellTiter-Glo Luminescent Cell Viability Assay. Data were normalized from 0% to 100% using DMSO (0%) and 56 μM staurosporine (100%). **(a)** HepG2, 24 h incubation. **(b)** HepG2, 72 h incubation. **(c)** HEK293, 24 h incubation. **(d)** HEK293, 72 h incubation. **(e)** HepaRG, 24 h incubation. **(f)** HepaRG, 72 h incubation. **(g)** Hepa 1-6, 24 h incubation. **(h)** Hepa 1-6, 72 h incubation. Data were derived from n=3 independent experiments and are presented as mean values +/- SD. Source data are provided as a Source Data file.

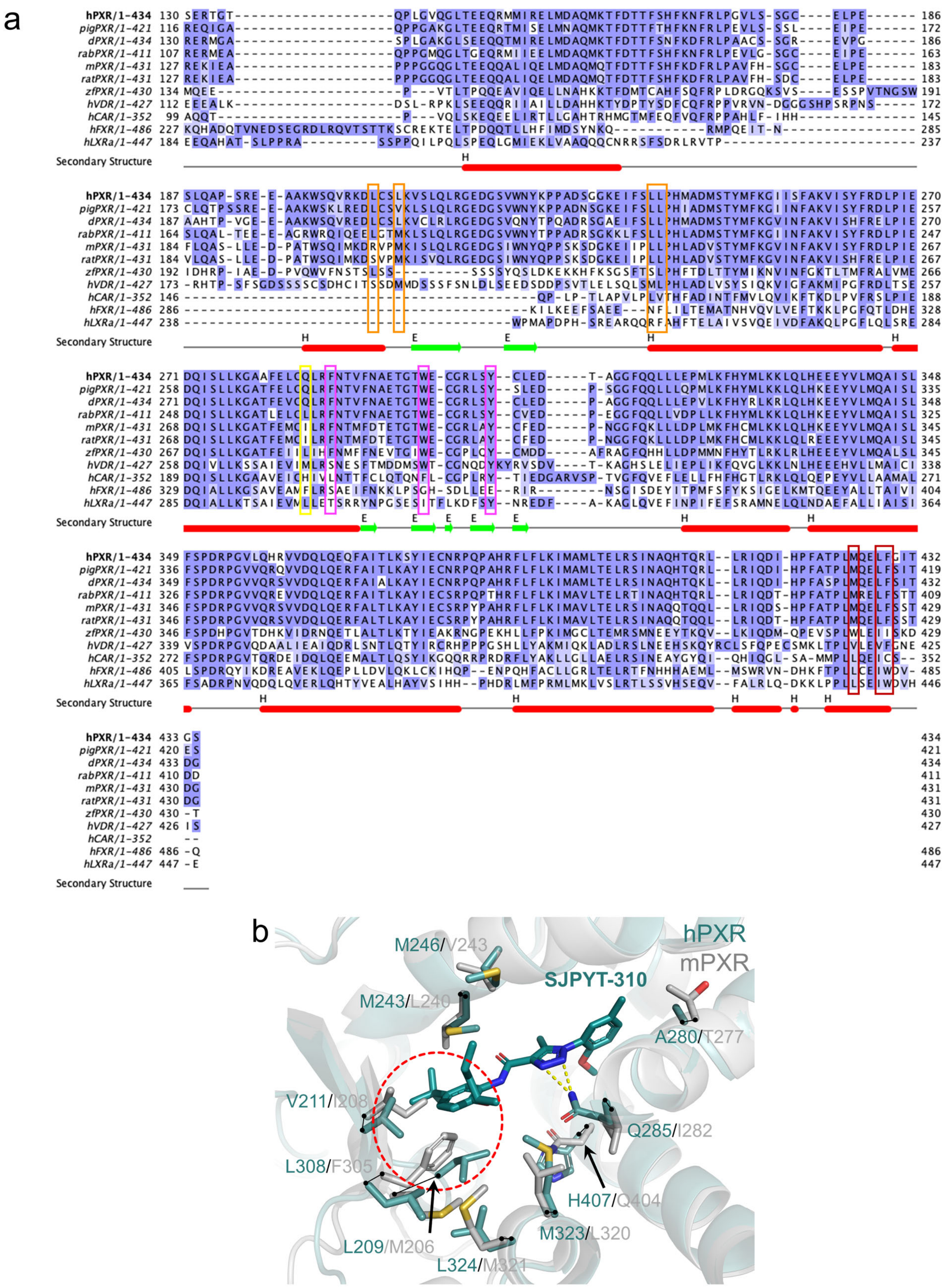

Supplementary Figure 7. PXR ligand binding pocket residues are not conserved across NRs. (a) Sequence alignment of indicated NR LBDs. The π-trap (violet), leucine cage (orange), Q285 (yellow), and α12 residues of interest (dark red) are highlighted in rectangular boxes. **(b)** SJPYT-310-bound PXR LBD (dark teal) is overlaid on the AlphaFold model of mPXR LBD (AF-O54915-F1, gray). PXR ligand binding pocket residues are not conserved in mPXR. The red dashed circle indicates clashes of SJPYT-310 with bulkier mPXR residues.

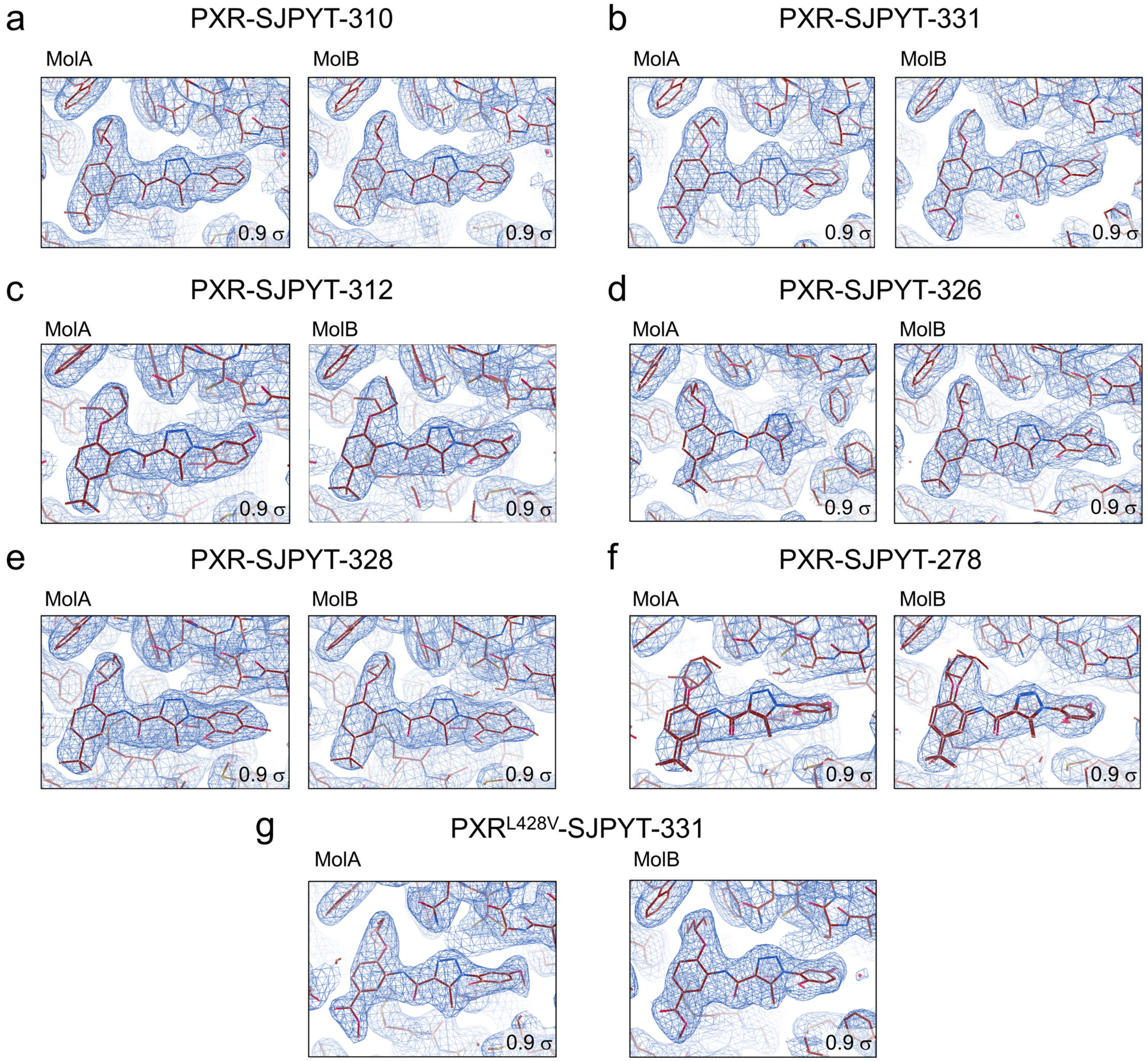

Supplementary Figure 8. **Identification of ligand binding sites.** Electron density maps (2Fo-Fc, blue) rendered at 0.9 σ for indicated structures: **(a)** WT + SJPYT-310, **(b)** WT + SJPYT-331, **(c)** WT + SJPYT-312, **(d)** WT + SJPYT-326, **(e)** WT + SJPYT-328, **(f)** WT + SJPYT-278, and **(g)** L428V + SJPYT-331 show unambiguous ligand density. Compounds were clearly identified in each monomer, except in **(d)**, where only partial SJPYT-326 electron density was observed in chain A. PXR LBD and PXR^{L428V} LBD are referred to as PXR and PXR^{L428V}, respectively.

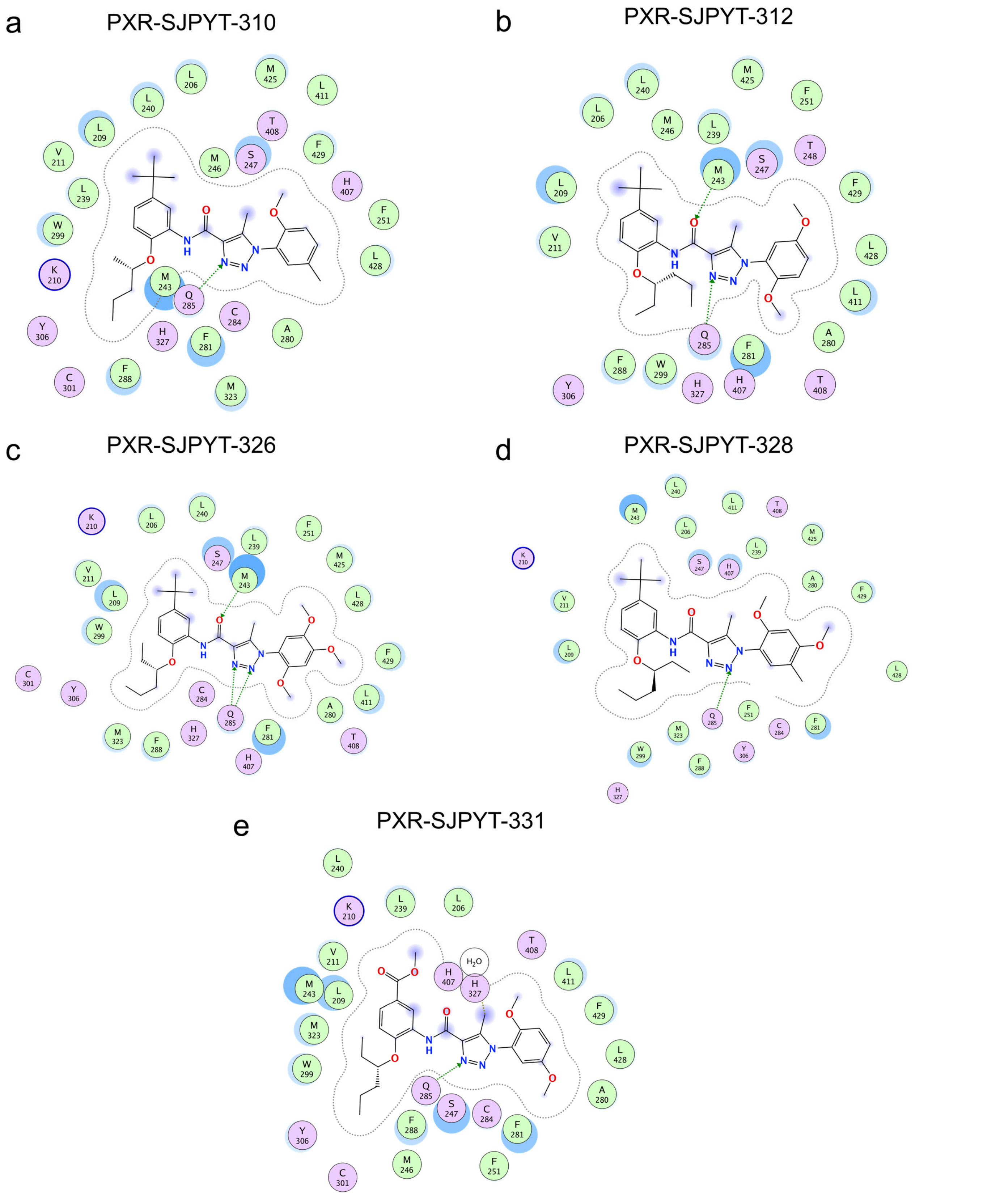

Supplementary Figure 9. Exploring ligand binding modes. 2D ligand interaction diagrams are shown for the indicated PXR LBD-ligand complexes: **(a)** WT + SJPYT-310, **(b)** WT + SJPYT-312, **(c)** WT + SJPYT-326, **(d)** WT + SJPYT-328, and **(e)** WT + SJPYT-331. Nonbonded interactions involving ligand atoms were determined within a radius of 4.5 Å (light green, hydrophobic residues; purple, polar residues; white, water molecules; dashed green lines, hydrogen bonds).

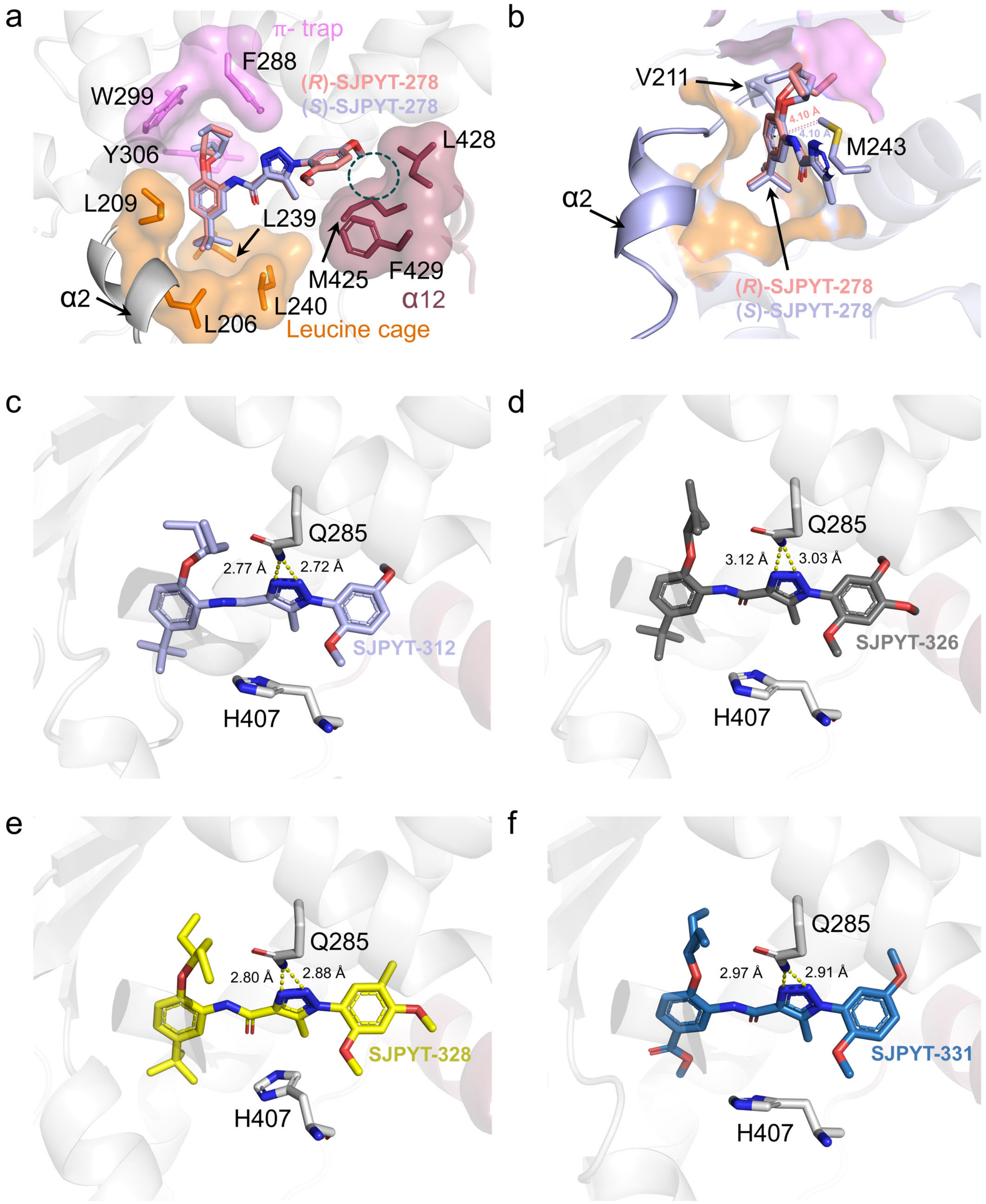

Supplementary Figure 10. **Ligand binding modes within the PXR ligand binding pocket**. **(a-b)** Orientations of the two SJPYT-278 enantiomers: (*R*), salmon; (*S*), light blue. Residues of α12 (dark red), π-trap (violet), and leucine cage (orange) are shown as stick representation with transparent surface model. The dashed circle indicates lack of SJPYT-278 interaction with the α12 cleft. **(c-f)** Orientations of indicated compounds are shown. The triazole ring of each ligand interacts with Q285 through N-H···N hydrogen bonds (dashed yellow lines).

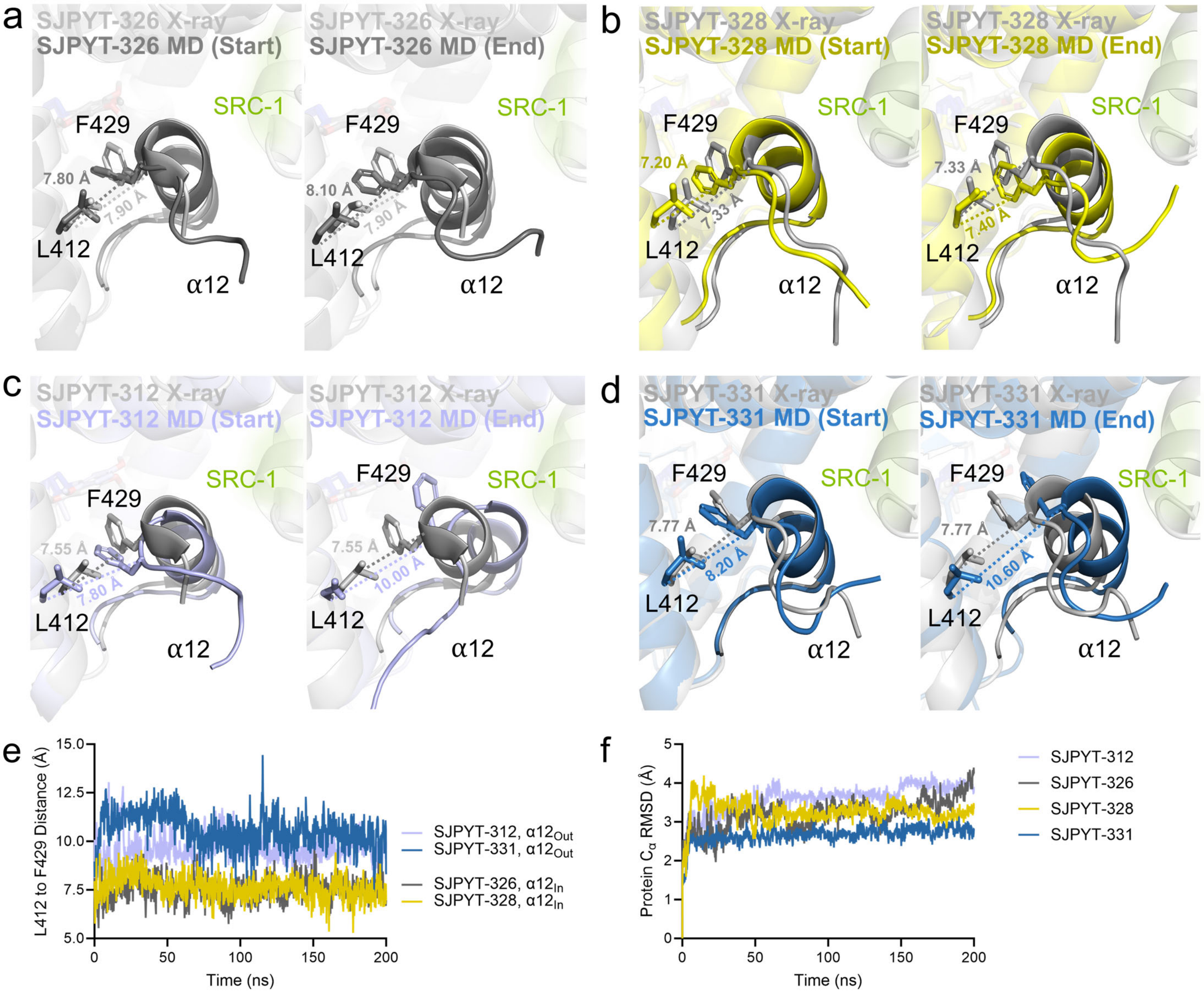

Supplementary Figure 11. α12 of antagonist-bound PXR LBD reorients to a position incompatible with SRC-1 binding. 200 ns molecular dynamics (MD) simulations were performed for PXR LBD bound to **(a-b)** agonists SJPYT-326 or SJPYT-328 or **(c-d)** antagonists SJPYT-312 or SJPYT-331. The SRC-1 peptide sequence was removed from the structures prior to simulation. The left panels show overlays of the PXR LBD-ligand-SRC-1 X-ray crystal structures (gray) and the models at the beginning of the simulations. The right panels show overlays of the PXR LBD-ligand-SRC-1 X-ray crystal structures and the models at the end of the simulations. **(e)** The distance between L412 C_{alpha} and F429 C_{alpha} was calculated for each frame of each simulation. A larger distance indicates outward motion of α12. **(f)** The root mean squared deviation (RMSD) of the protein Cα for each MD system is plotted. The systems quickly reached equilibrium. Source data for **(e-f)** are provided as a Source Data file.

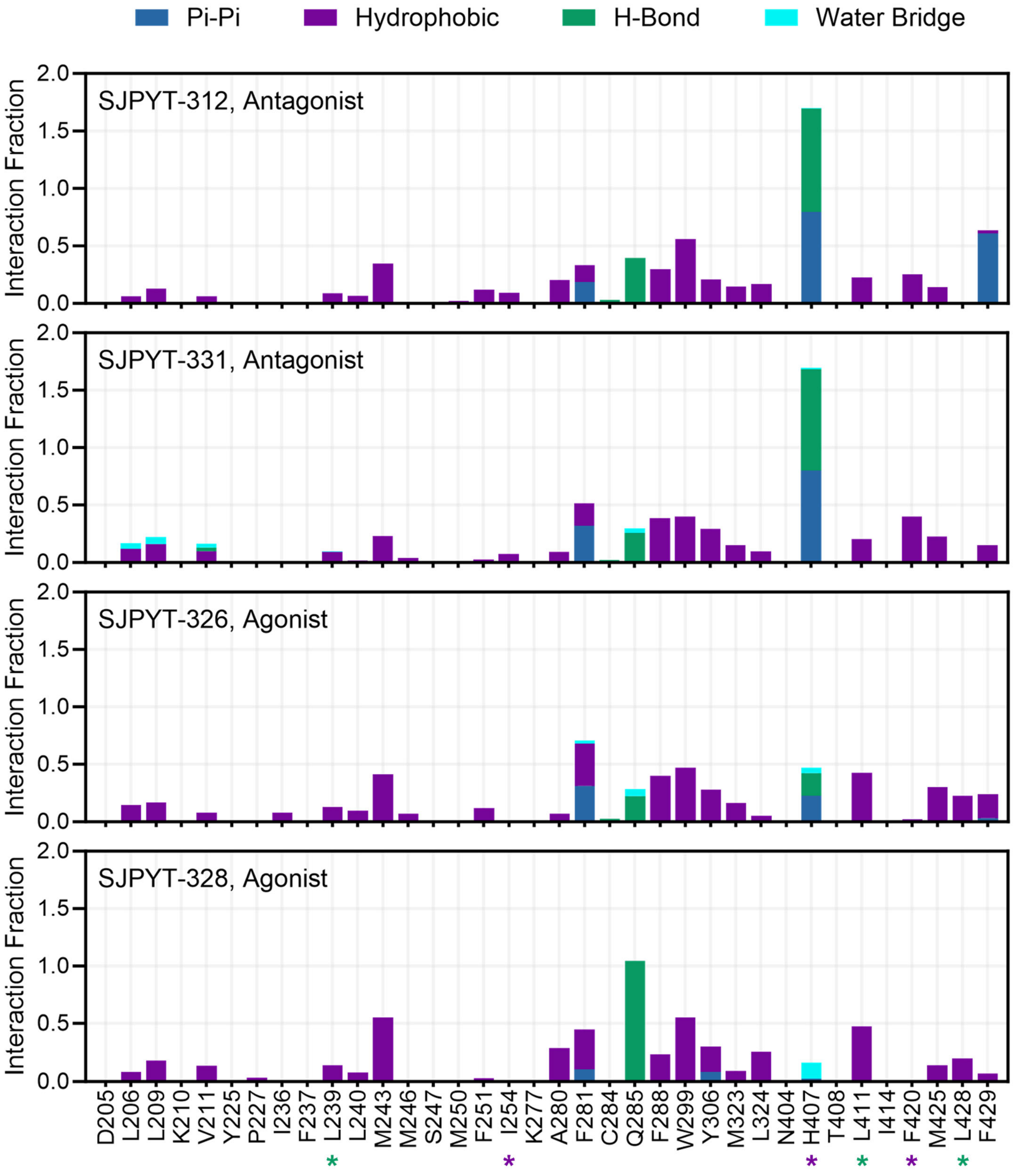

Supplementary Figure 12. Agonists and antagonists have distinct interactions within the PXR ligand binding pocket. Residue interaction diagrams are shown for each MD simulation, quantifying the fraction of simulation time that each ligand-residue interaction occurred. Only residues with ≥1% for at least one simulation course are shown. T-tests were performed for average interaction fractions of agonists versus antagonists for each residue, and the asterisks below the residue names indicate significantly more interaction with agonists (green) or antagonists (purple) with $p \leq 0.05$ (p = 0.039, 0.014, 0.012, 0.013, 0.050, and 0.006 for L239, I254, H407, L411, F420, and L428, respectively). Source data for are provided as a Source Data file.

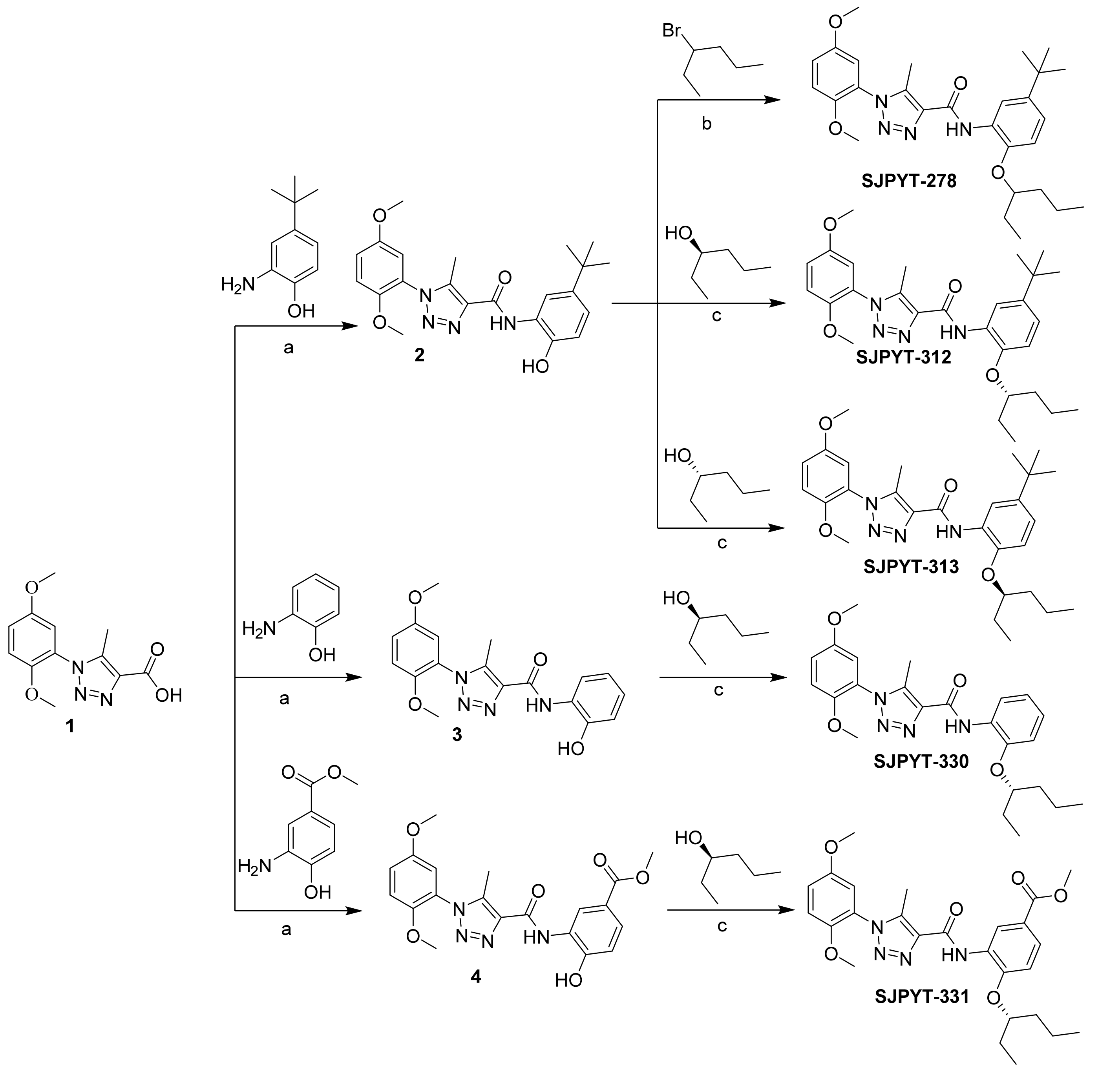

Supplementary Figure 13. Synthesis of SJPYT-278, 312, 313, 330, 331 and 332. Reagents and conditions: **(a)** EDCI, HOBt, DIEA, DMF, room temperature, overnight; **(b)** Cs_2CO_3, DMF, 60°C, overnight; **(c)** PPh_3, DIAD, DCM, room temperature, overnight.

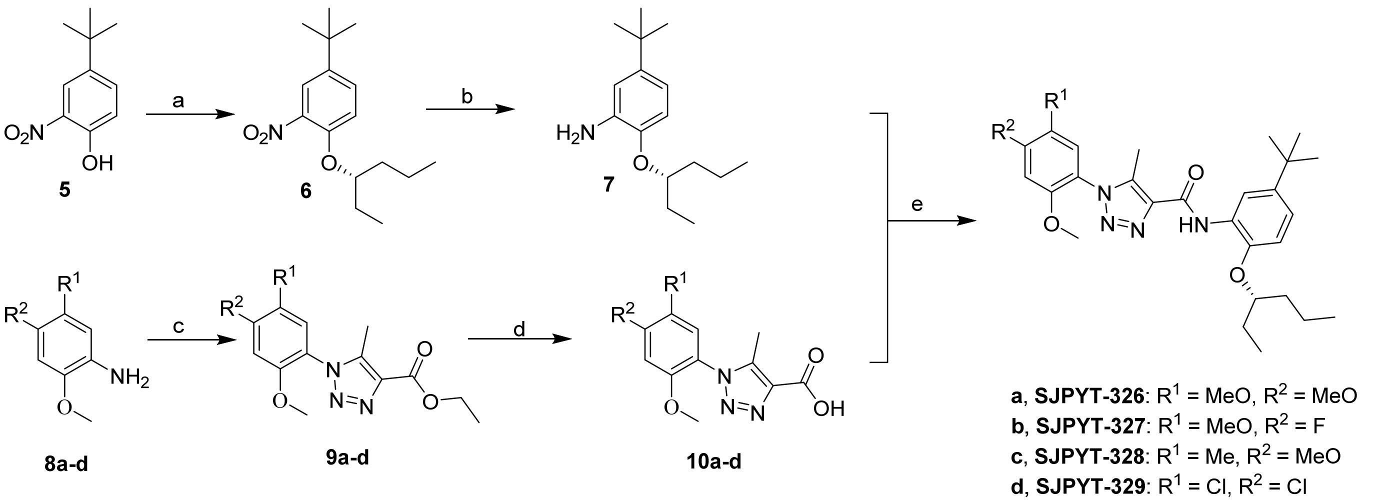

a, **SJPYT-326**: R^1 = MeO, R^2 = MeO
b, **SJPYT-327**: R^1 = MeO, R^2 = F
c, **SJPYT-328**: R^1 = Me, R^2 = MeO
d, **SJPYT-329**: R^1 = Cl, R^2 = Cl

Supplementary Figure 14. Synthesis of SJPYT-326, 327, 328 and 329. Reagents and conditions: **(a)** (*R*)-pentan-2-ol, PPh_3, DIAD, DCM, room temperature, overnight; **(b)** $HCOONH_4$, Pd/C, CH_3OH, 60 °C, overnight; **(c)** $TiCl_4$, ethyl 2-diazo-3-oxobutanoate, Toluene, 80 °C, overnight; **(d)** LiOH, CH_3OH/H_2O, room temperature, overnight; **(e)** EDCI, HOBt, DIEA, DMF, room temperature, overnight.

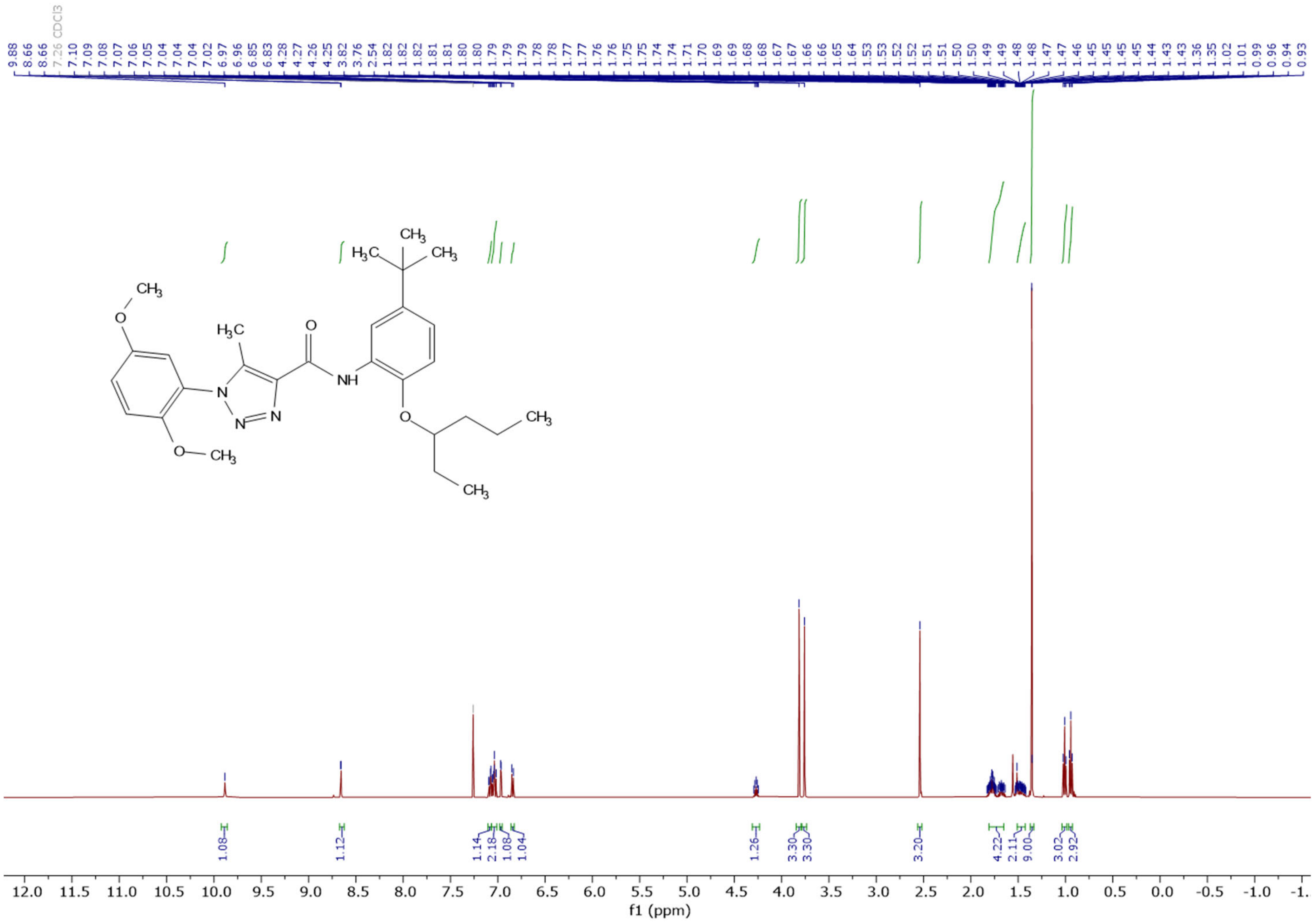

Supplementary Figure 15. ^{1}H NMR of compound SJPYT-278.

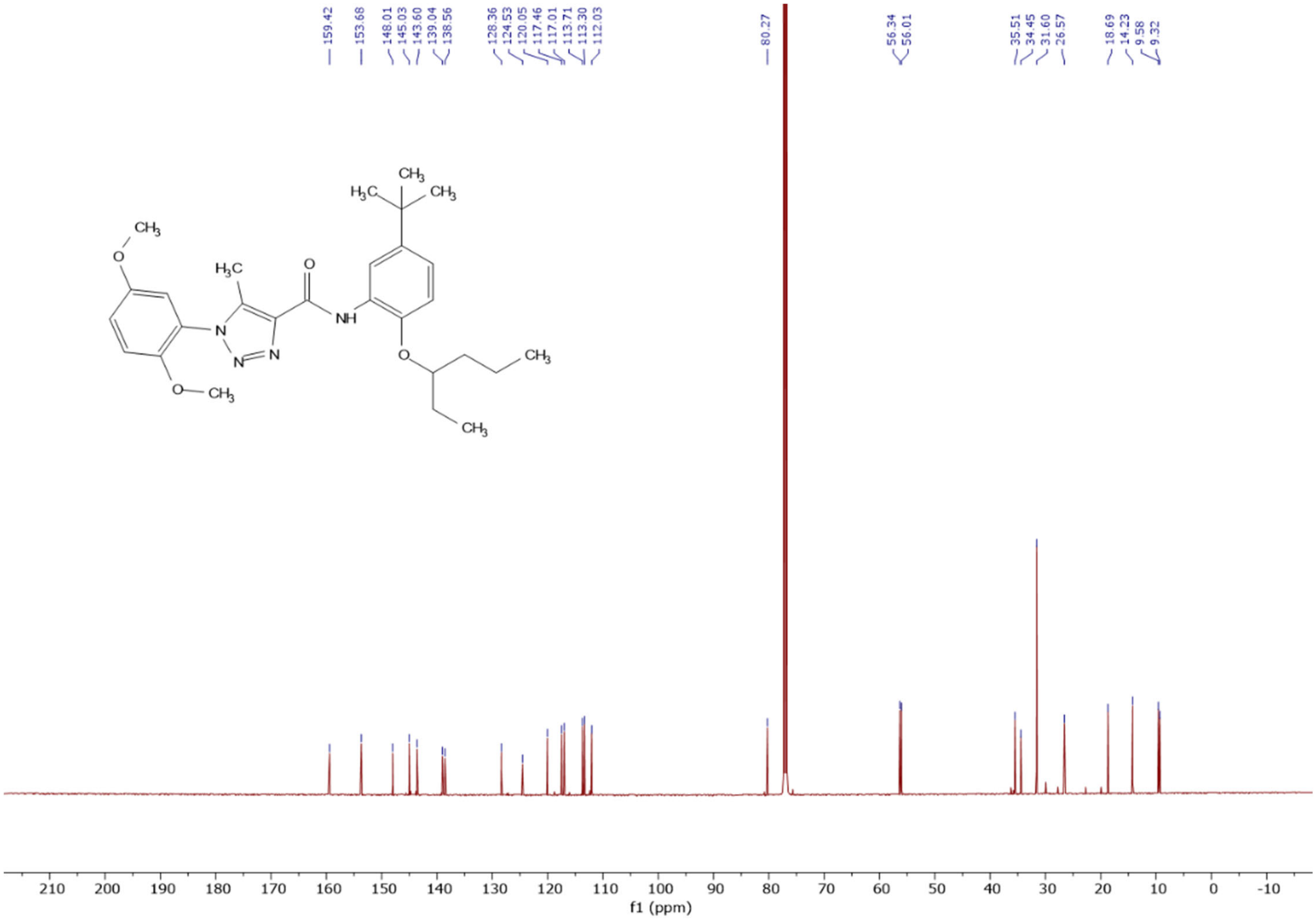

Supplementary Figure 16. ^{13}C NMR of compound SJPYT-278.

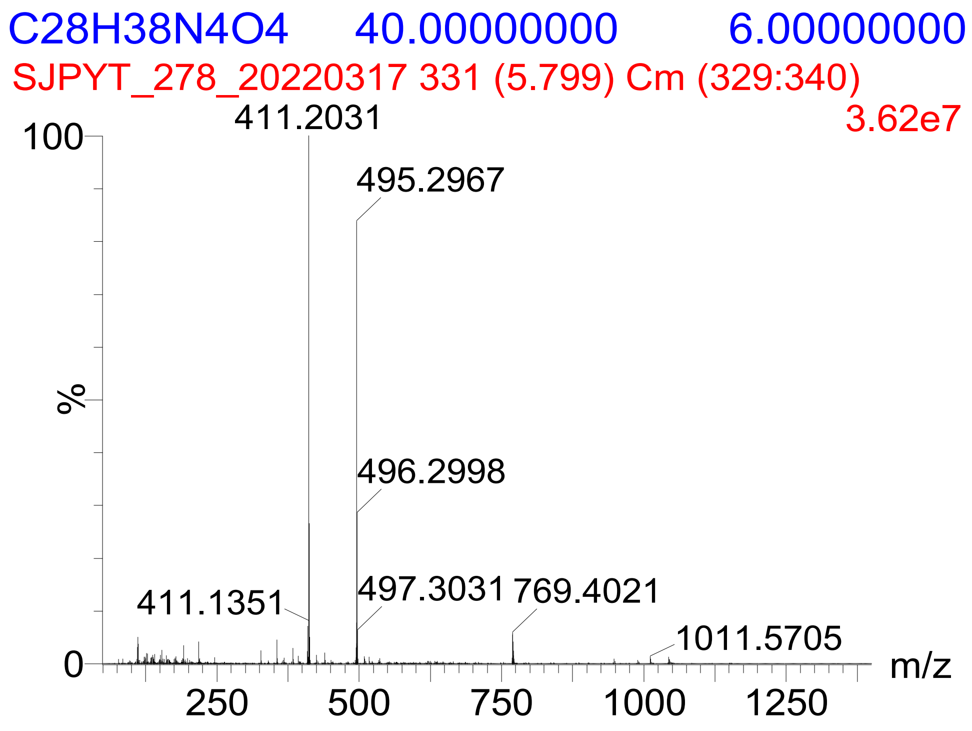

Supplementary Figure 17. HRMS of compound SJPYT-278.

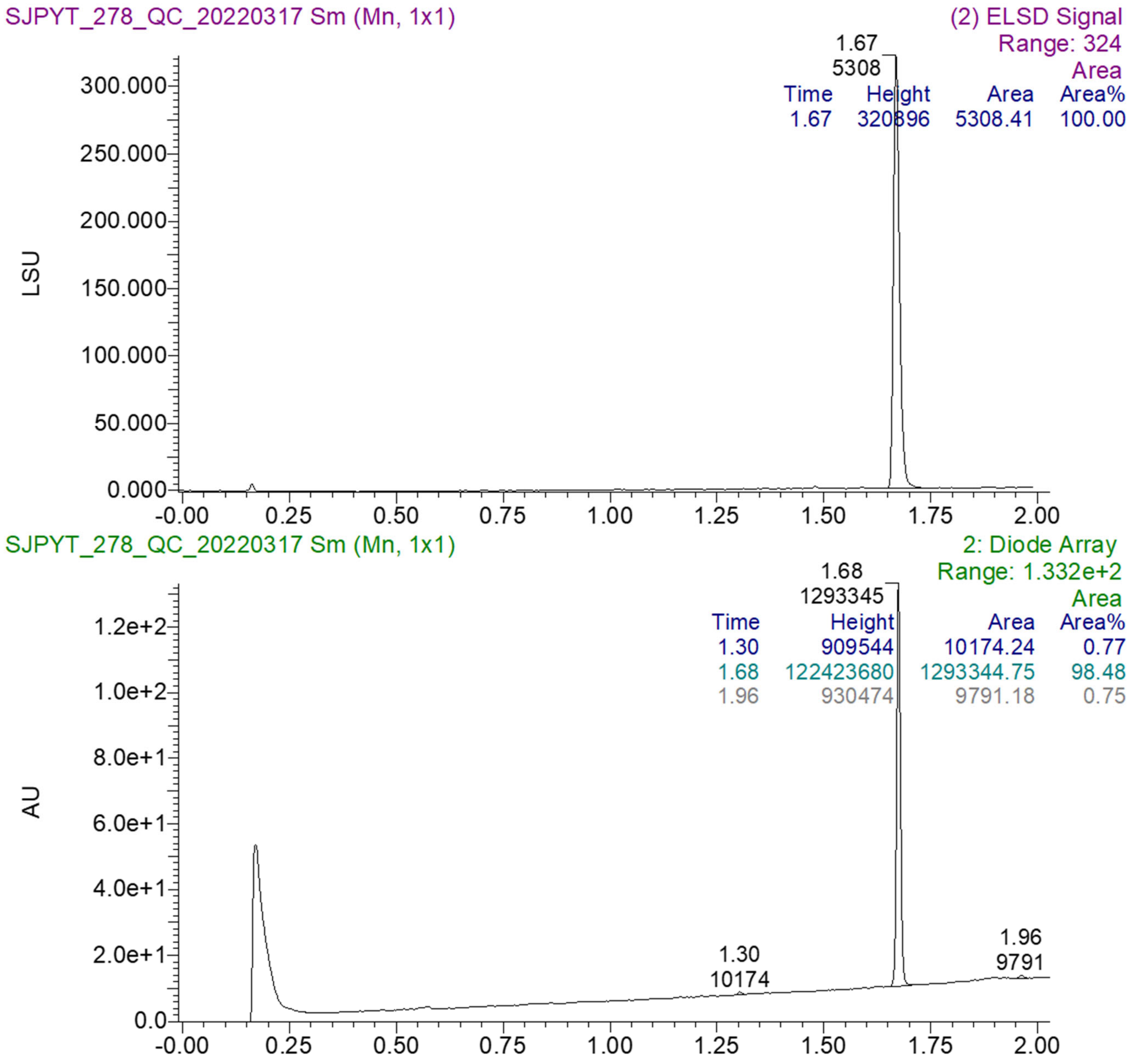

Supplementary Figure 18. HPLC of compound SJPYT-278.

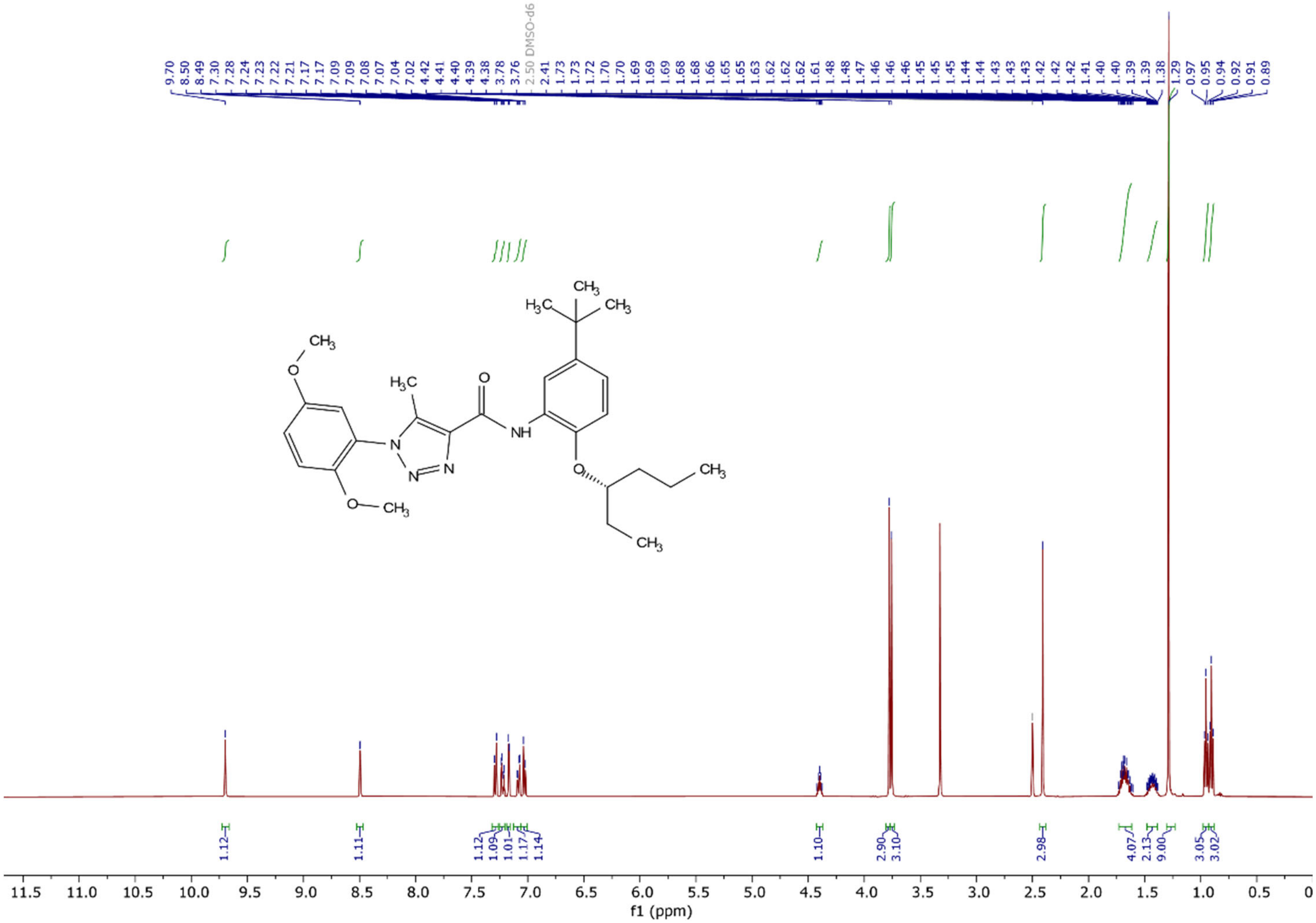

Supplementary Figure 19. ^{1}H NMR of compound SJPYT-312.

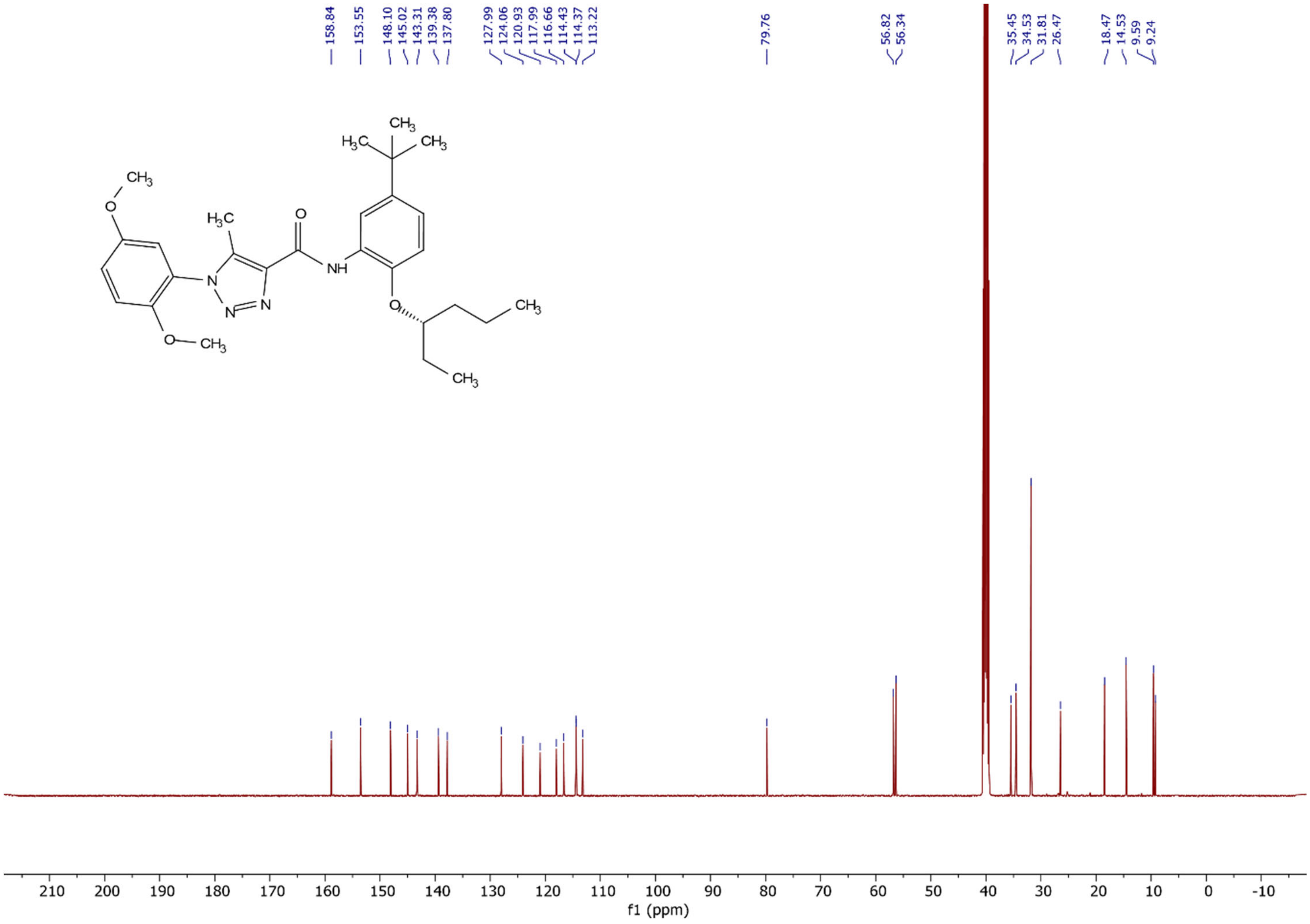

Supplementary Figure 20. ^{13}C NMR of compound SJPYT-312.

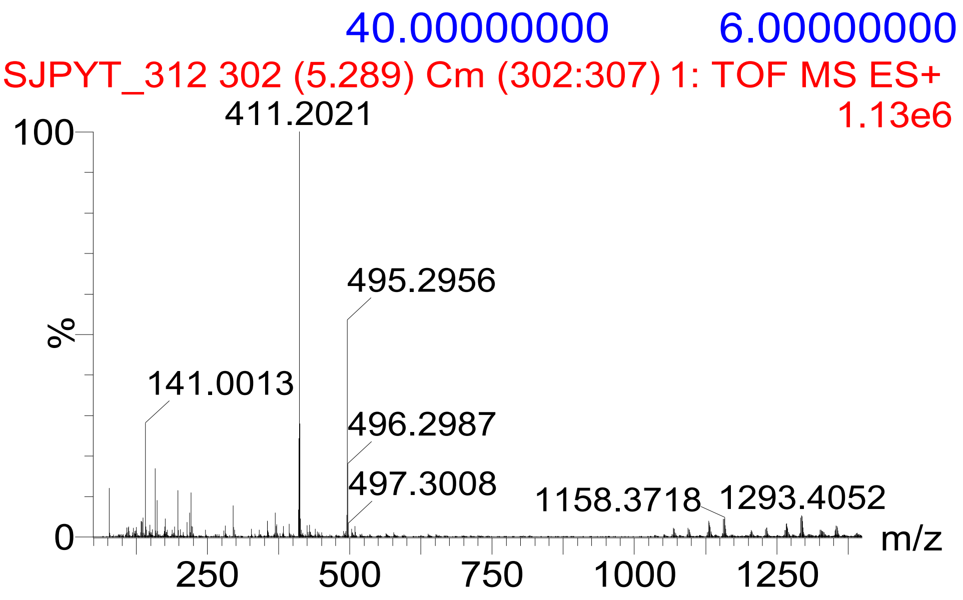

Supplementary Figure 21. HRMS of compound SJPYT-312.

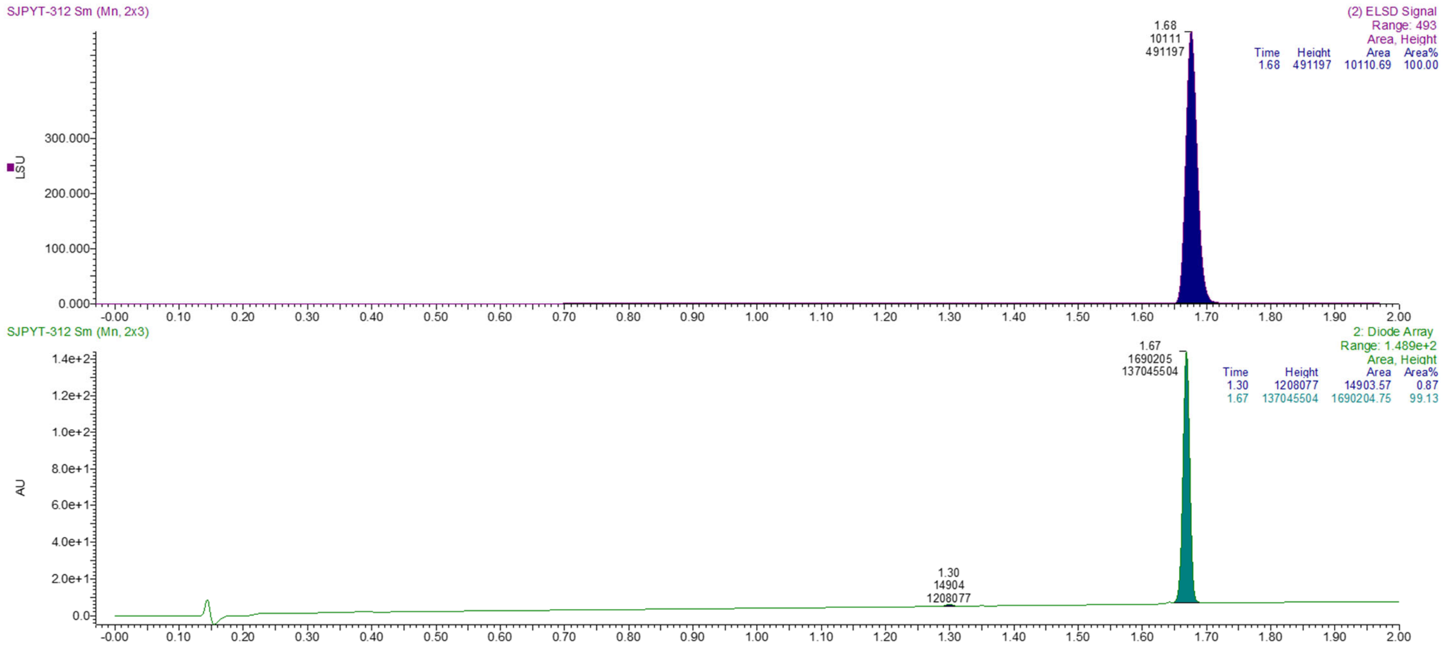

Supplementary Figure 22. HPLC of compound SJPYT-312.

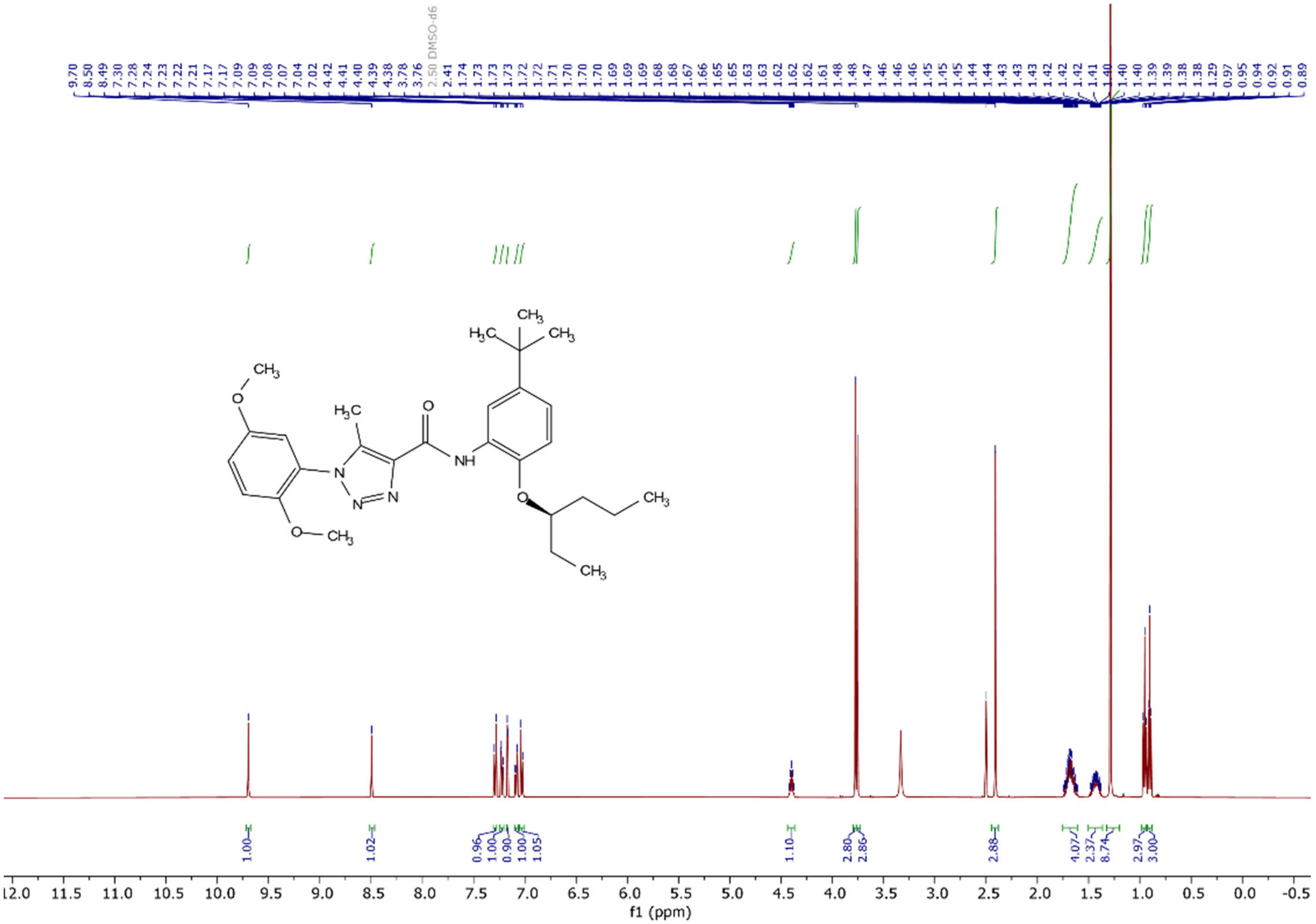

Supplementary Figure 23. ^{1}H NMR of compound SJPYT-313.

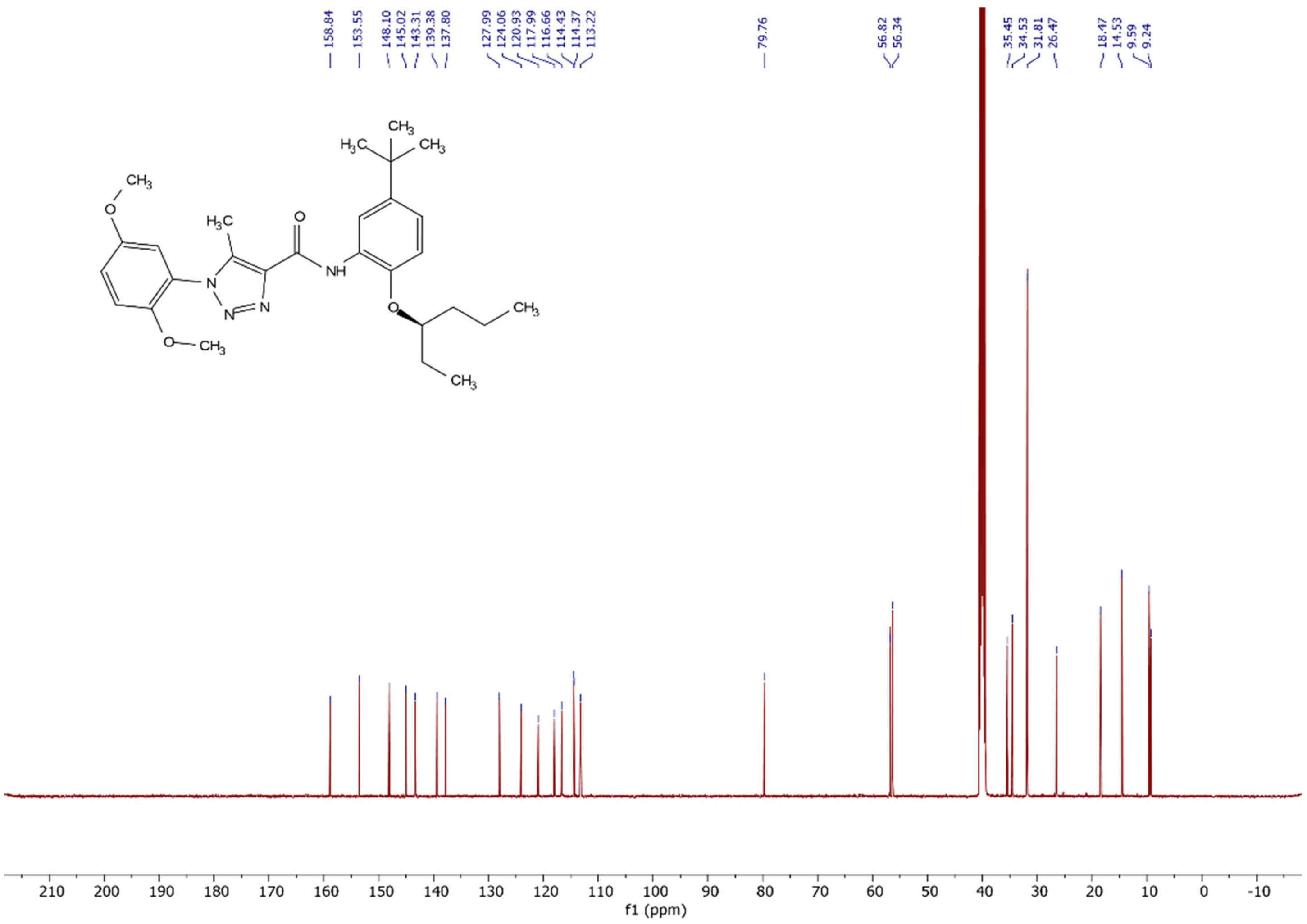

Supplementary Figure 24. ^{13}C NMR of compound SJPYT-313.

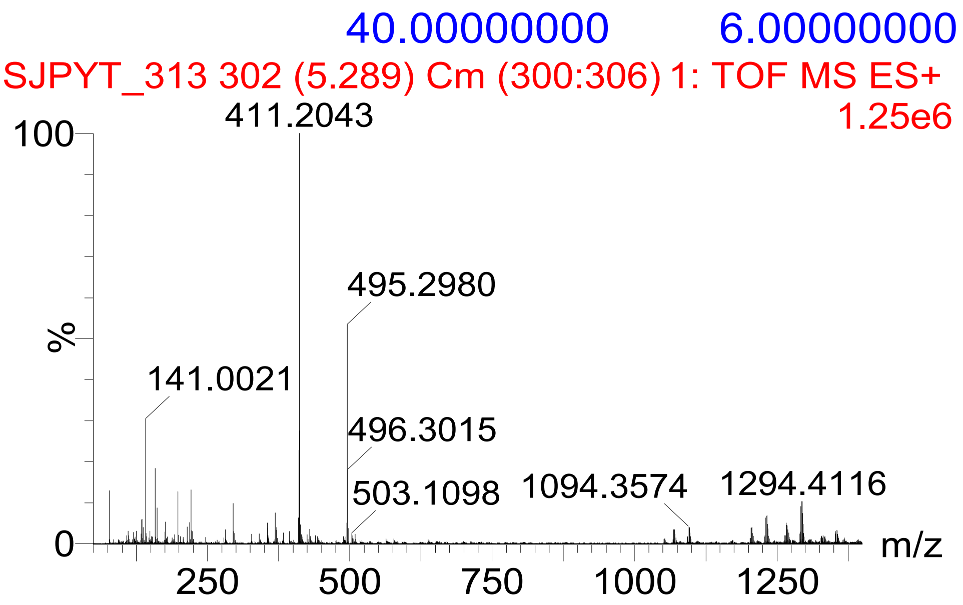

Supplementary Figure 25. HRMS of compound SJPYT-313.

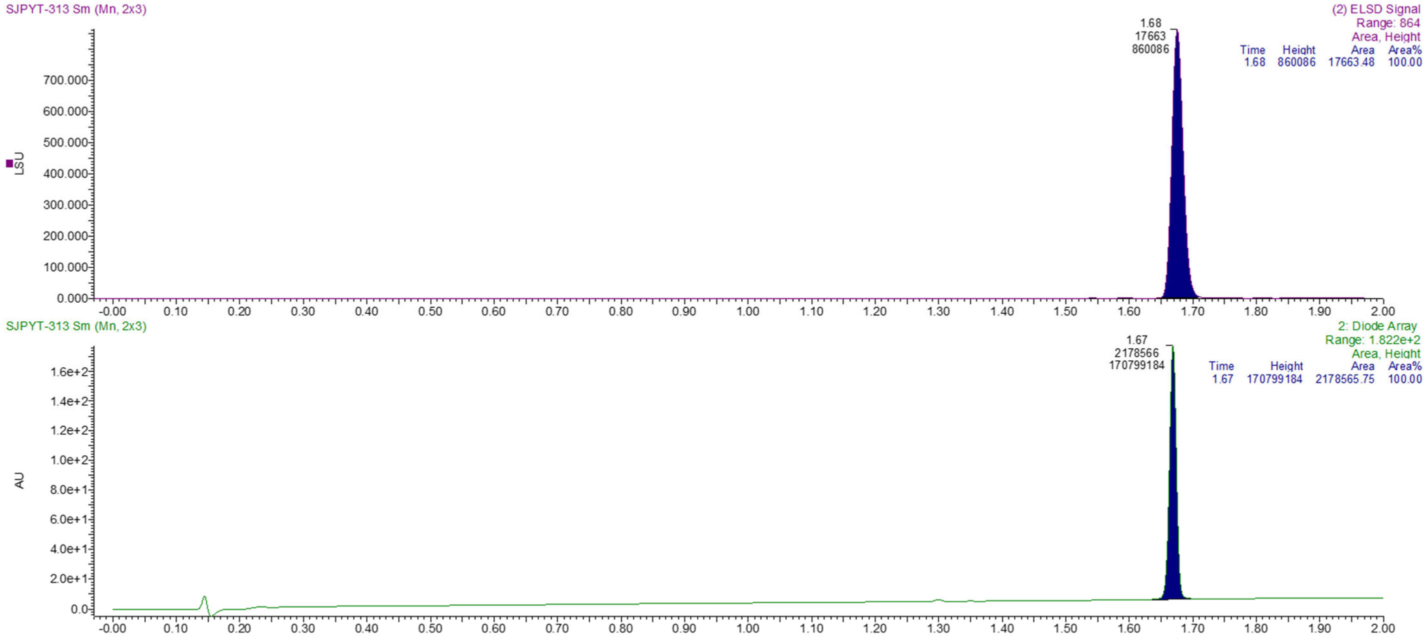

Supplementary Figure 26. HPLC of compound SJPYT-313.

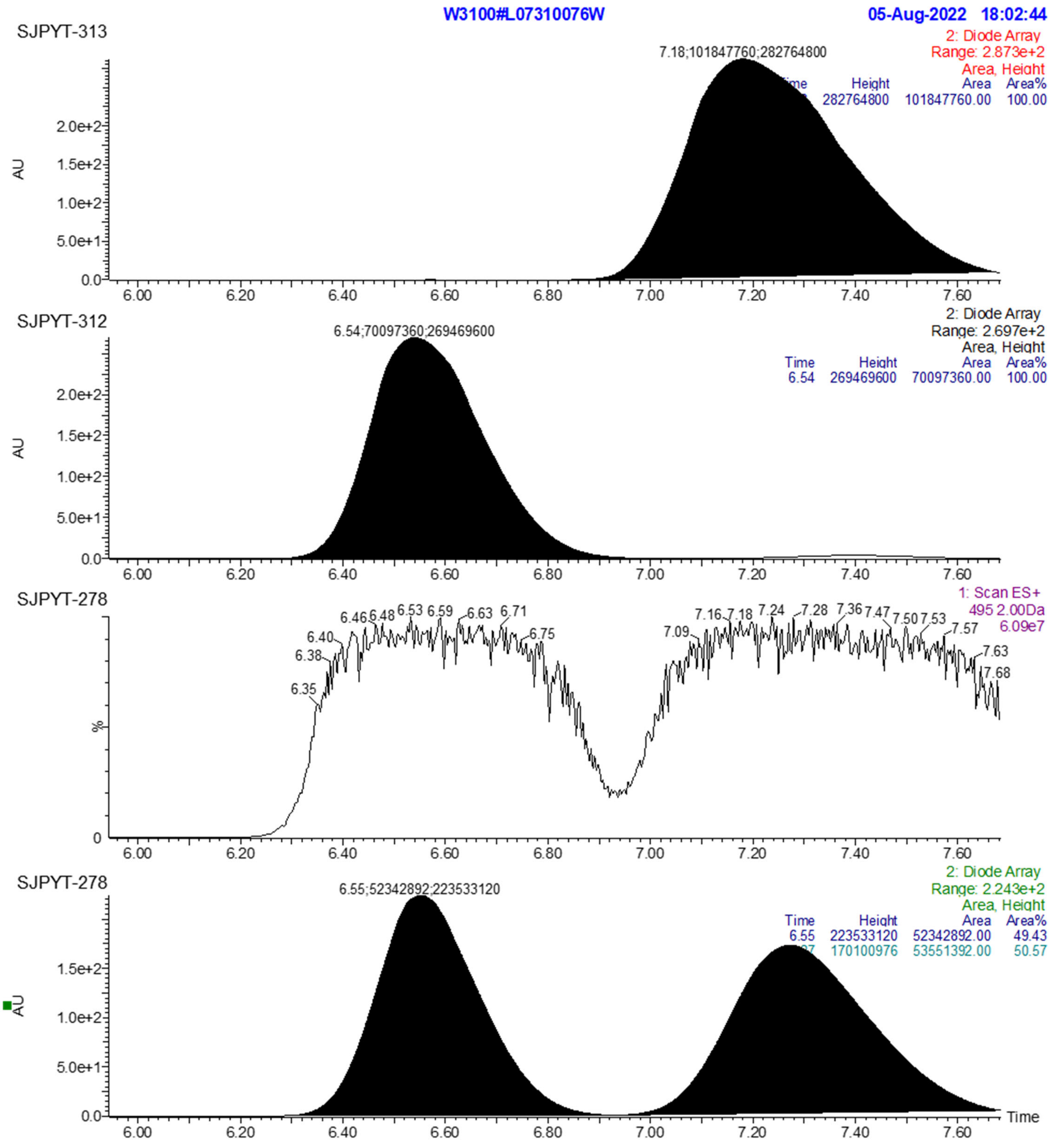

Supplementary Figure 27. Supercritical fluid chromatography (SFC) spectra of compounds SJPYT-278, SJPYT-312 and SJPYT-313.

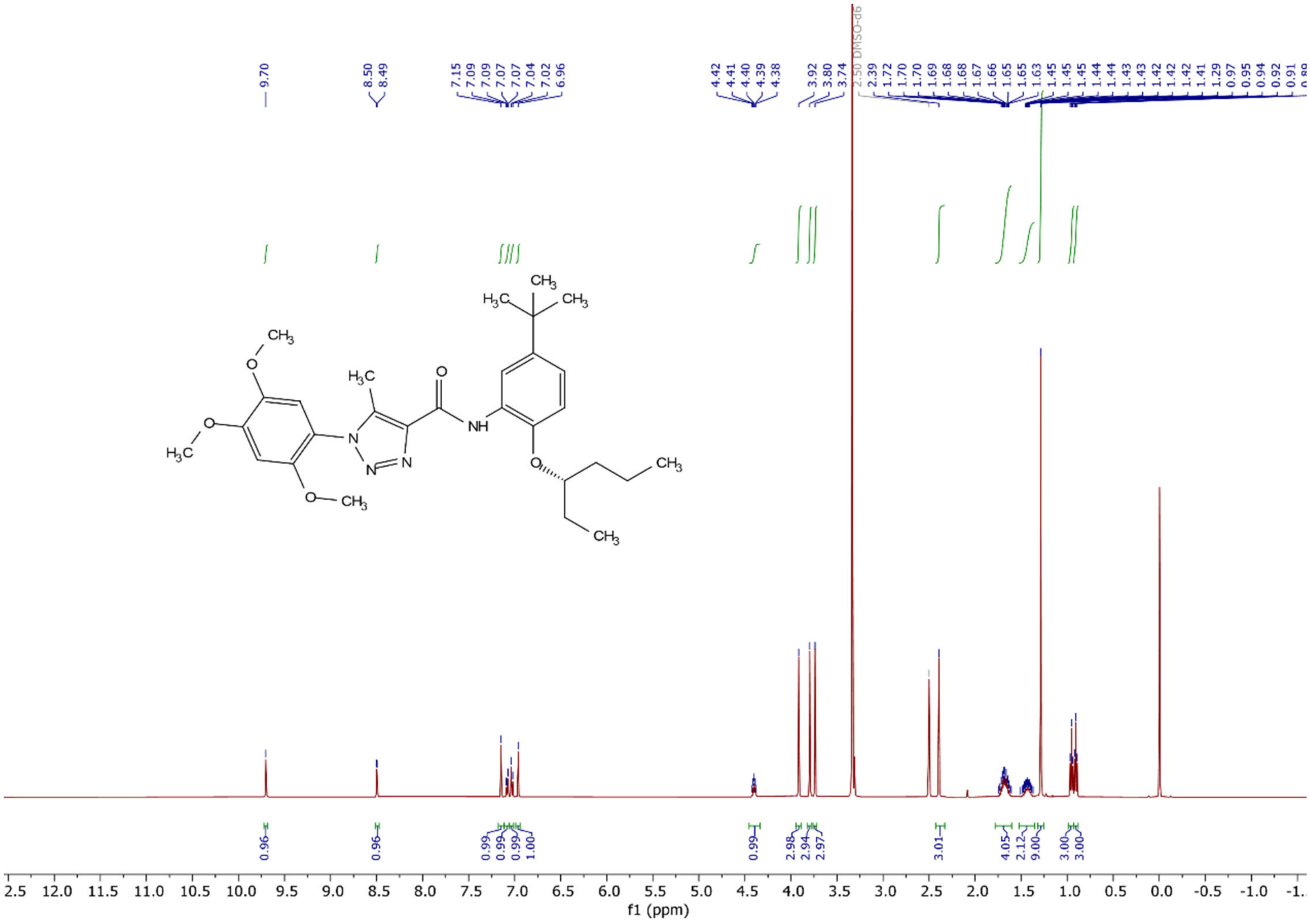

Supplementary Figure 28. ^{1}H NMR of compound SJPYT-326.

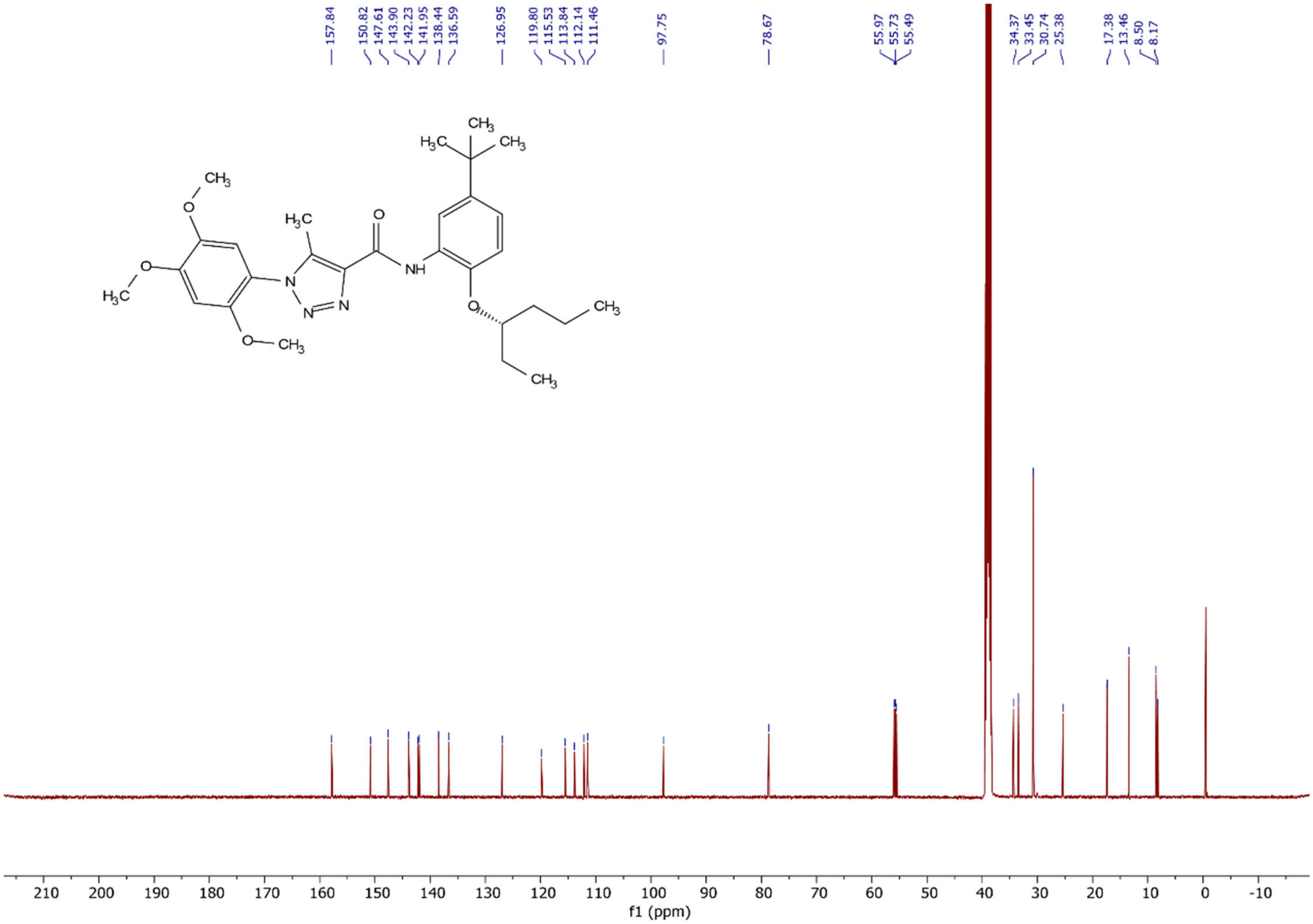

Supplementary Figure 29. ^{13}C NMR of compound SJPYT-326.

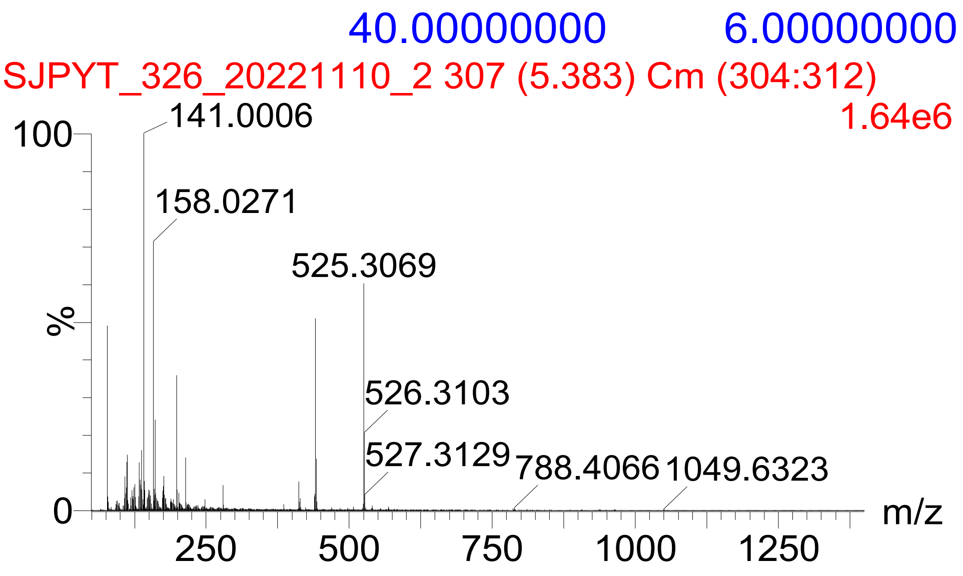

Supplementary Figure 30. HRMS of compound SJPYT-326.

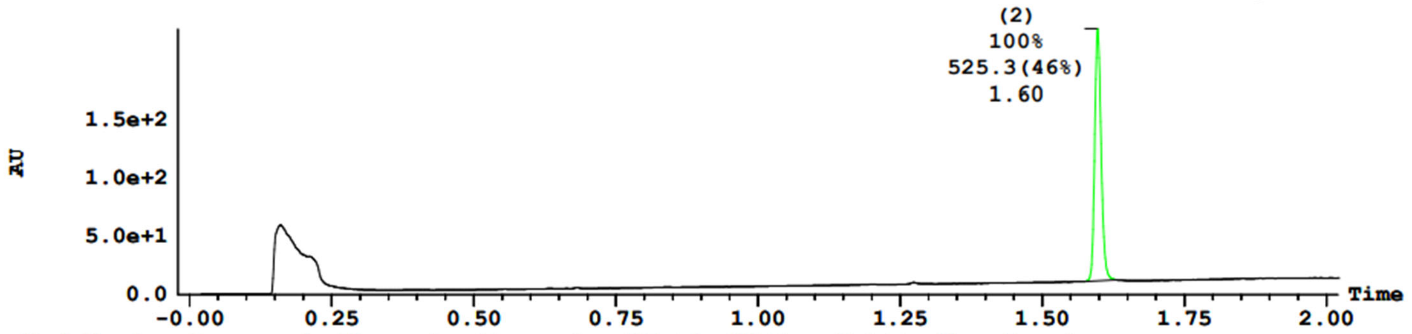

Peak Number	Compound	Time	AreaAbs	Area %Total	Width	Height	Mass Found
2	Found	1.60	3e+006	100.00	0	2e+008	525.3071

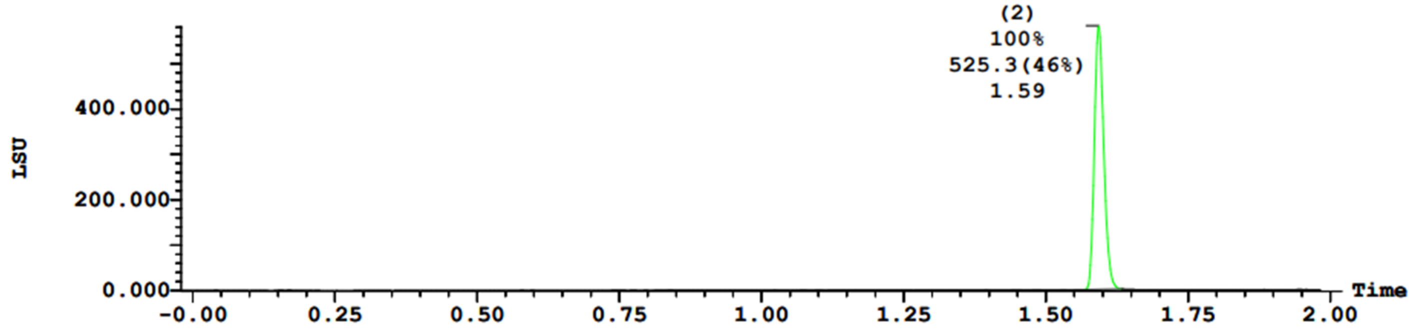

Peak Number	Compound	Time	AreaAbs	Area %Total	Width	Height	Mass Found
2	Found	1.59	1e+004	100.00	0	6e+005	525.3071

Supplementary Figure 31. HPLC of compound SJPYT-326.

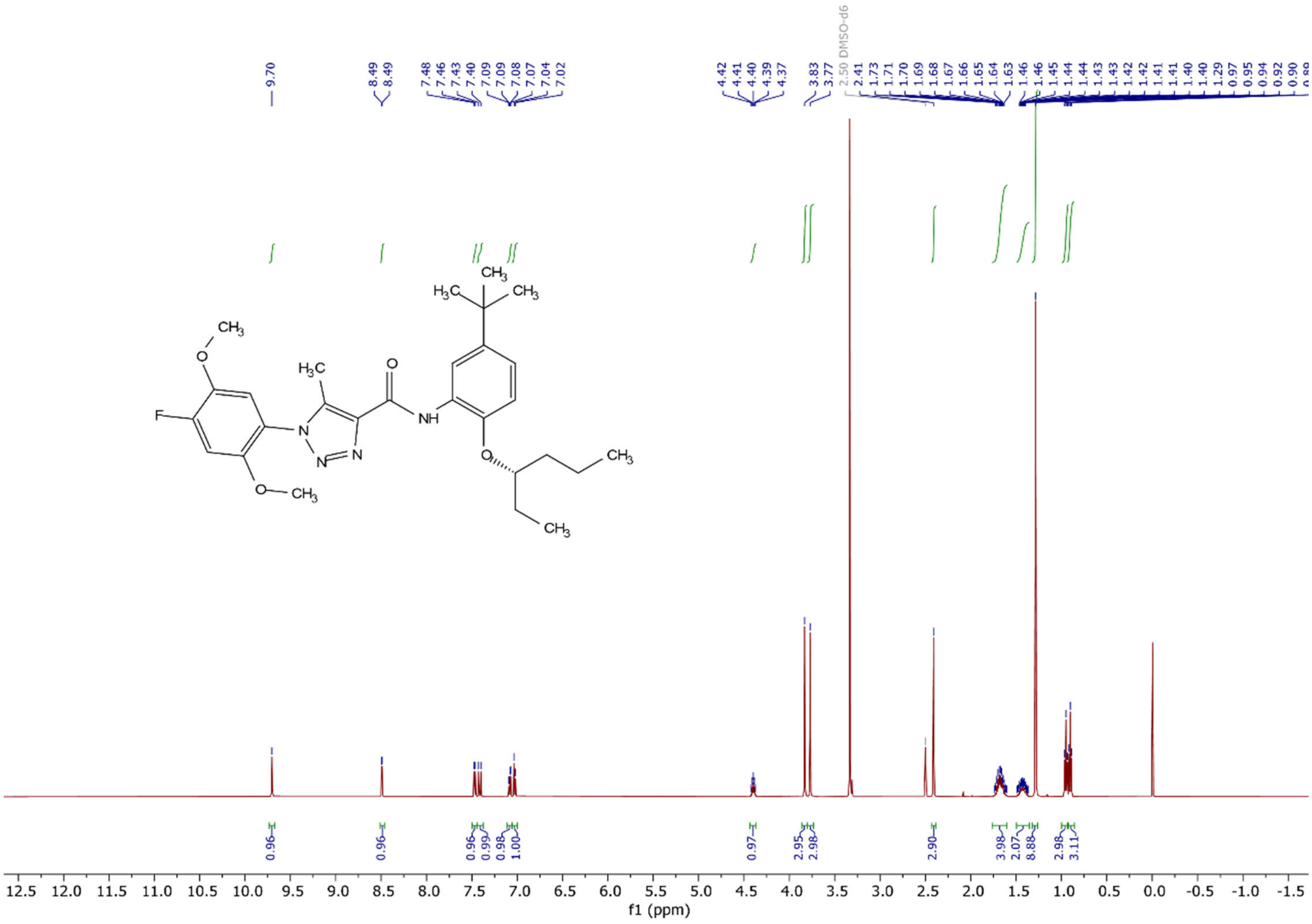

Supplementary Figure 32. ^{1}H NMR of compound SJPYT-327.

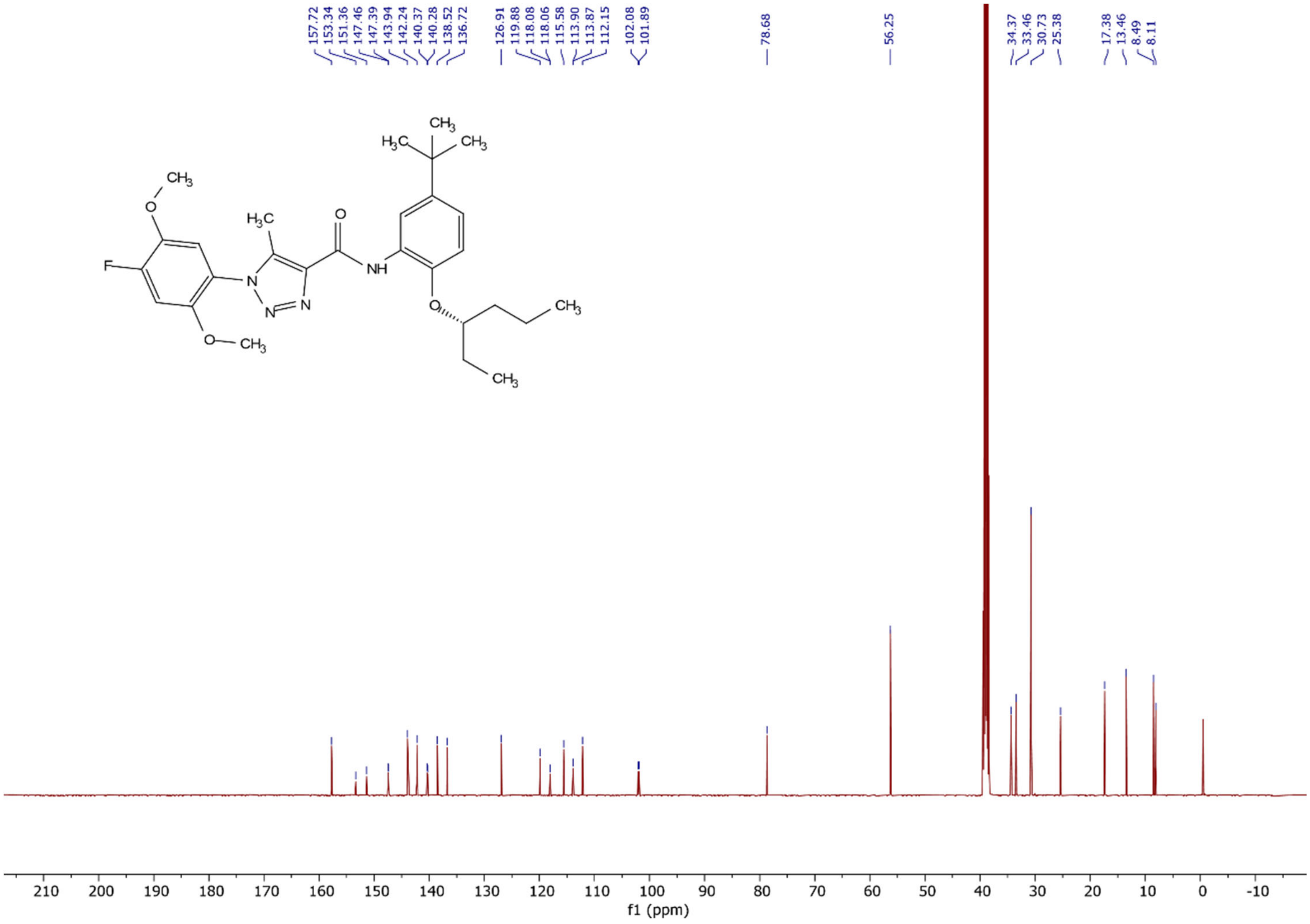

Supplementary Figure 33. ^{13}C NMR of compound SJPYT-327.

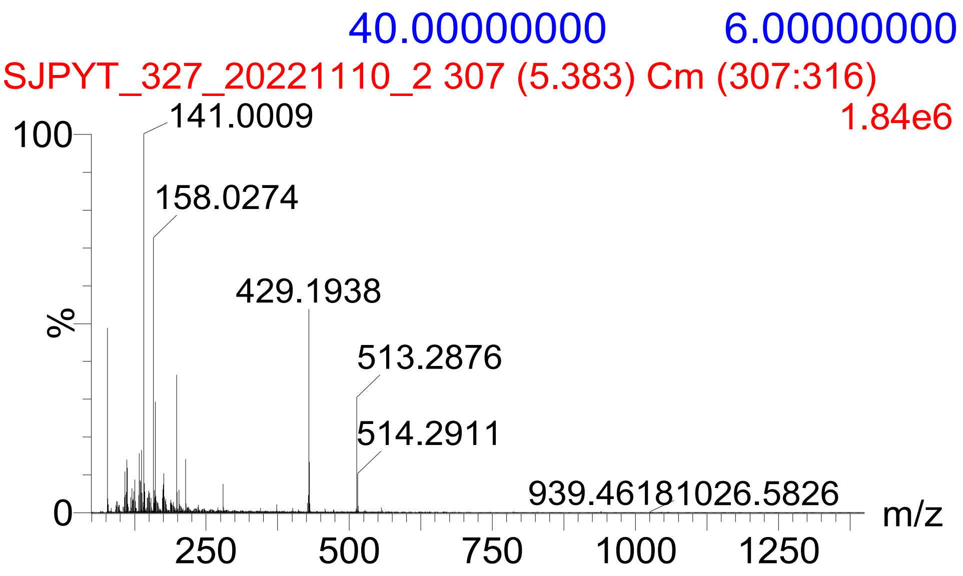

Supplementary Figure 34. HRMS of compound SJPYT-327.

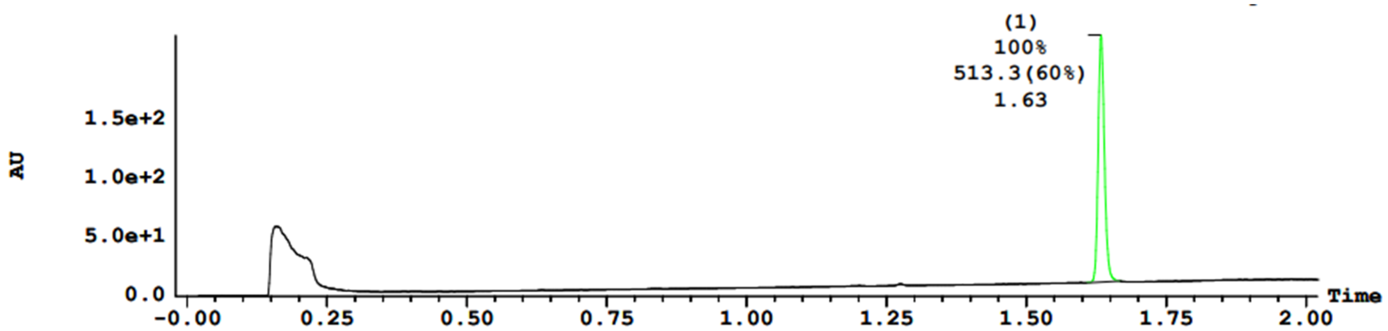

Peak Number	Compound	Time	AreaAbs	Area %Total	Width	Height	Mass Found
1	Found	1.63	3e+006	100.00	0	2e+008	513.2872

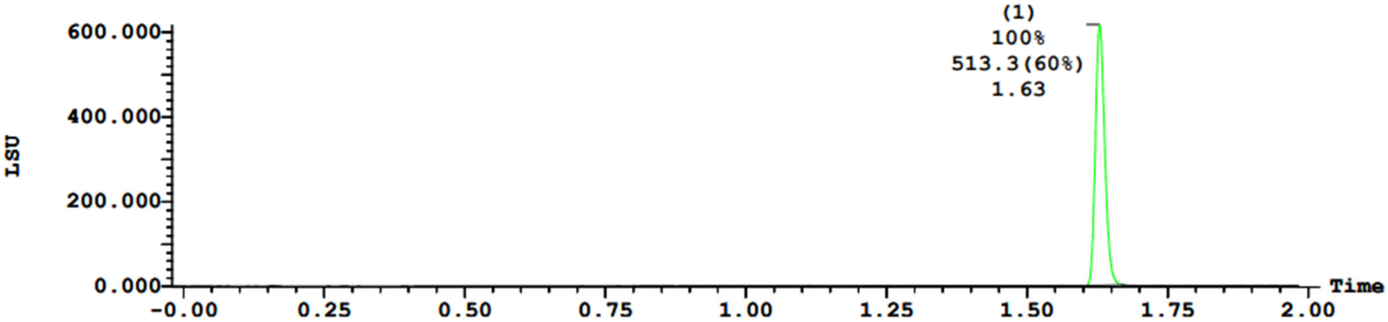

Peak Number	Compound	Time	AreaAbs	Area %Total	Width	Height	Mass Found
1	Found	1.63	1e+004	100.00	0	6e+005	513.2872

Supplementary Figure 35. HPLC of compound SJPYT-327.

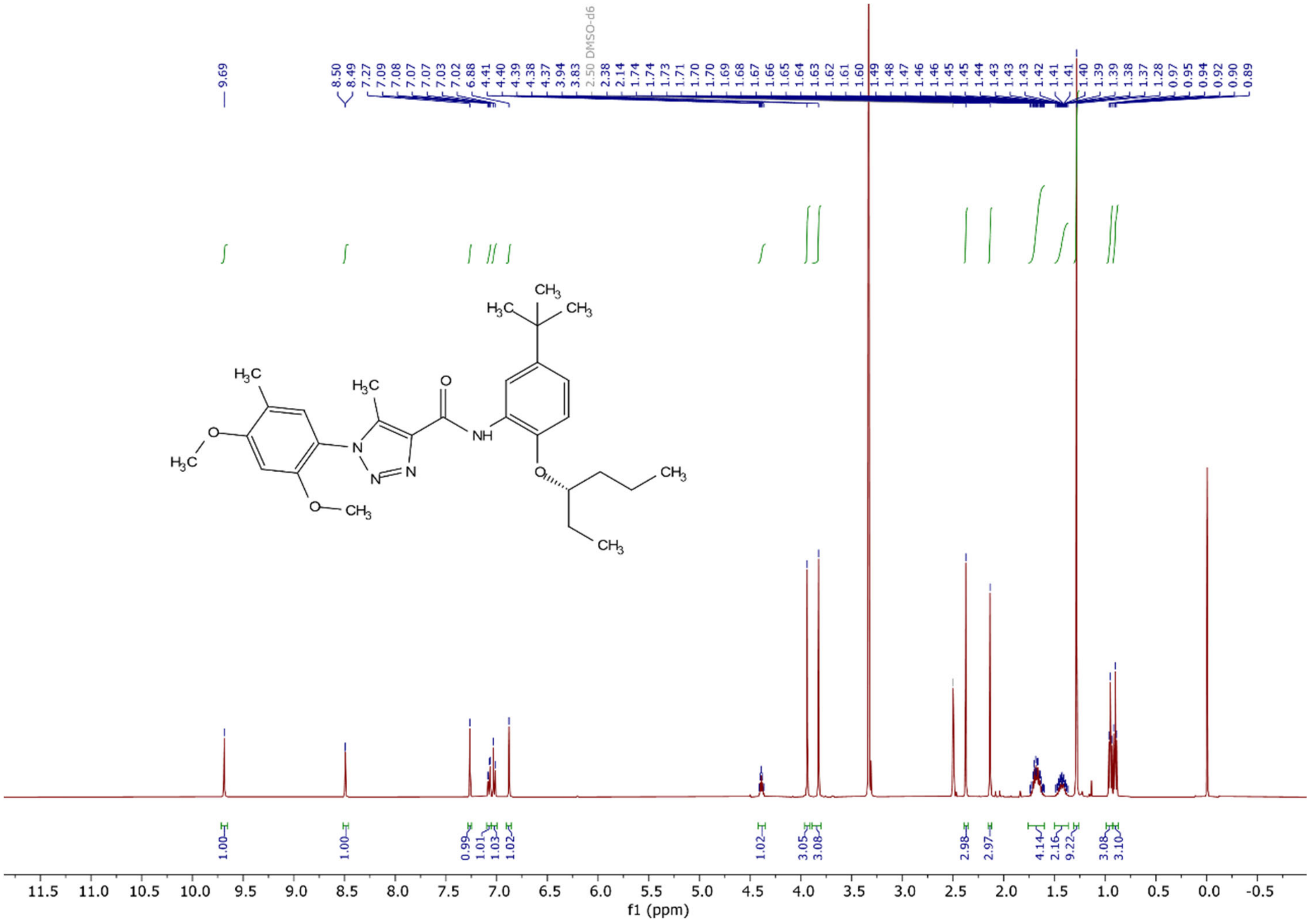

Supplementary Figure 36. ^{1}H NMR of compound SJPYT-328.

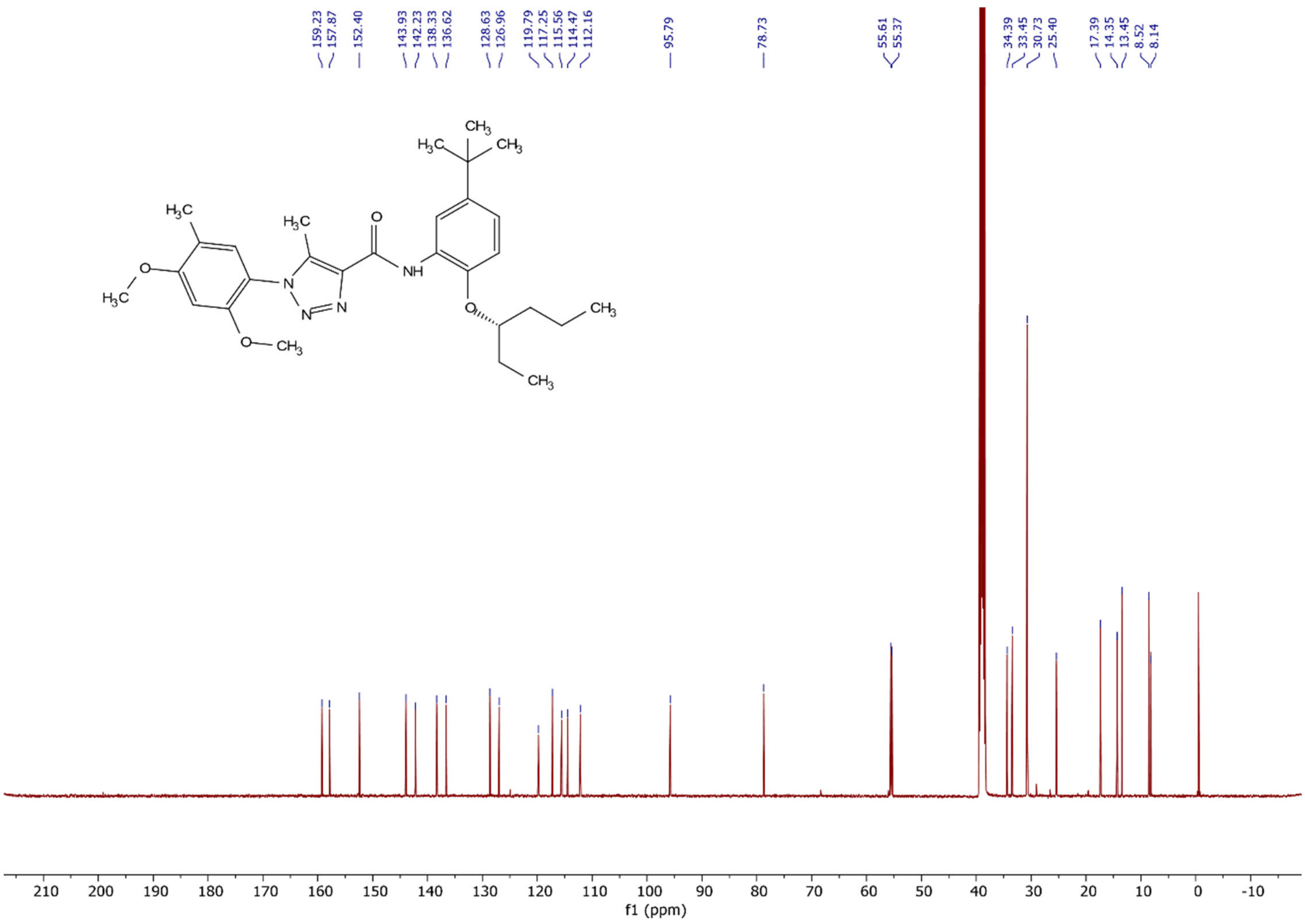

Supplementary Figure 37. ^{13}C NMR of compound SJPYT-328.

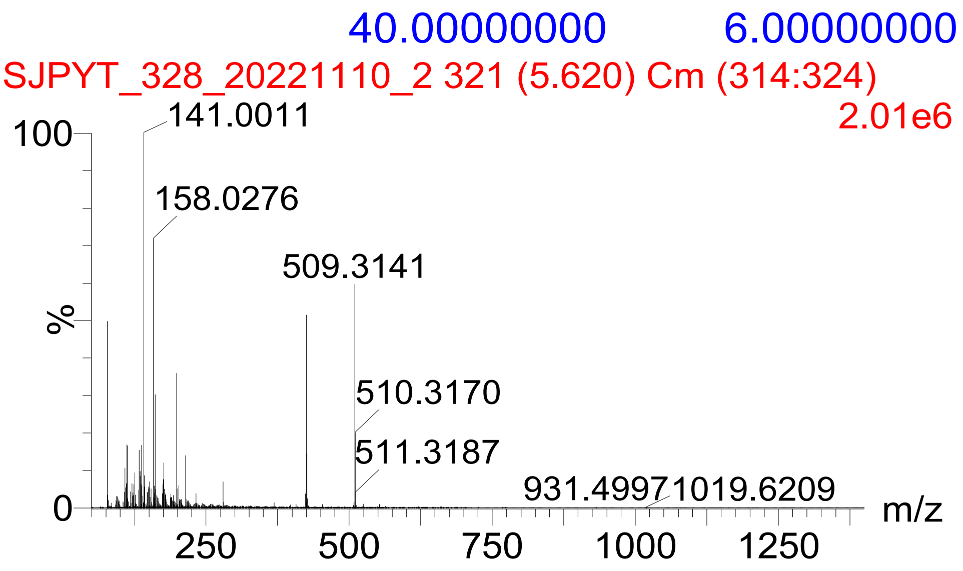

Supplementary Figure 38. HRMS of compound SJPYT-328.

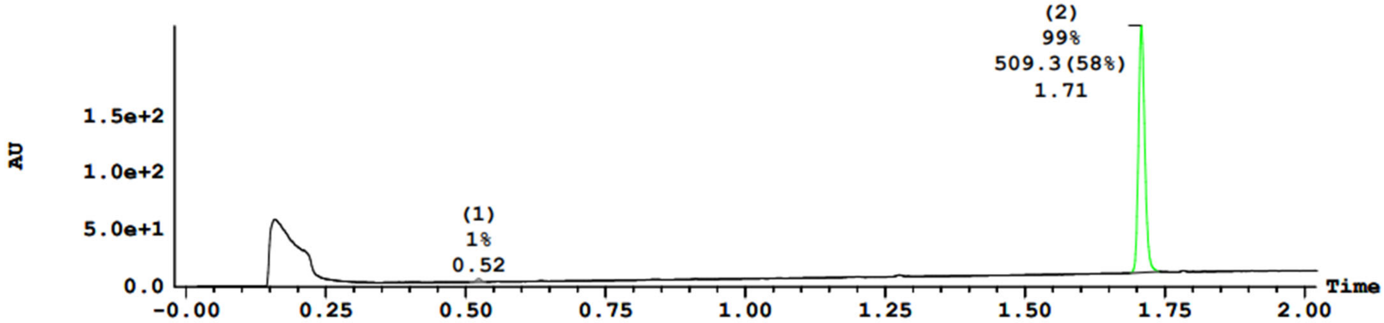

Peak Number	Compound	Time	AreaAbs	Area %Total	Width	Height	Mass Found
1		0.52	3e+004	1.04	0	3e+006	
2	Found	1.71	3e+006	98.96	0	2e+008	509.3122

(1) ELSD Signal Smooth (Mn, 2x2)

663.893
Range: 664.378

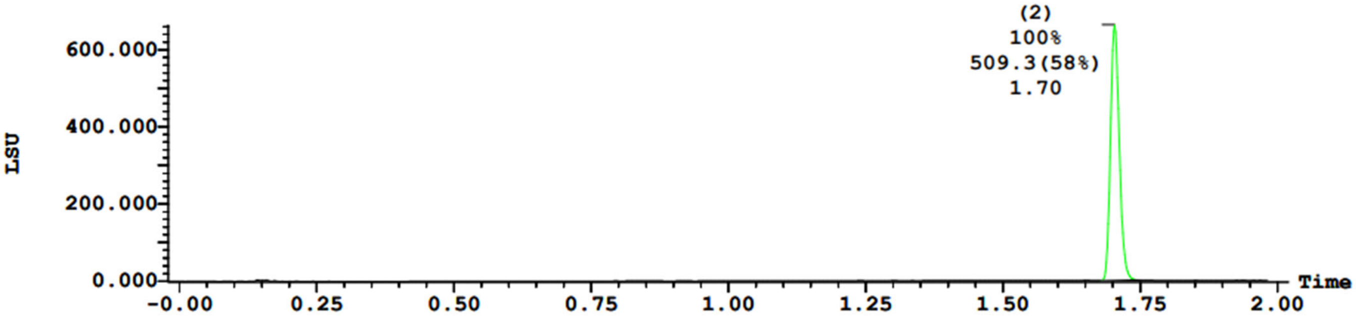

Peak Number	Compound	Time	AreaAbs	Area %Total	Width	Height	Mass Found
2	Found	1.70	1e+004	100.00	0	7e+005	509.3122

Supplementary Figure 39. HPLC of compound SJPYT-328.

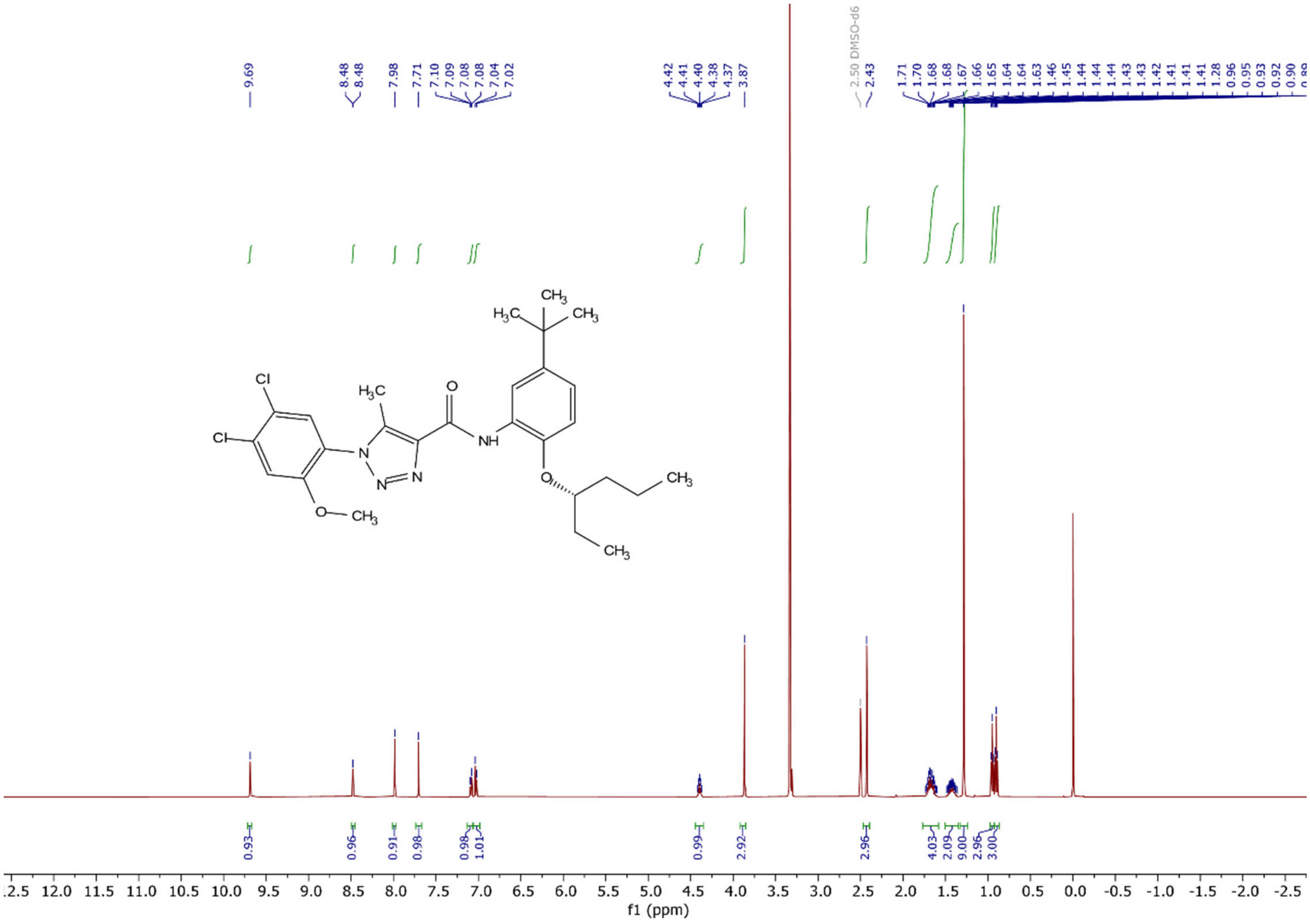

Supplementary Figure 40. ^{1}H NMR of compound SJPYT-329.

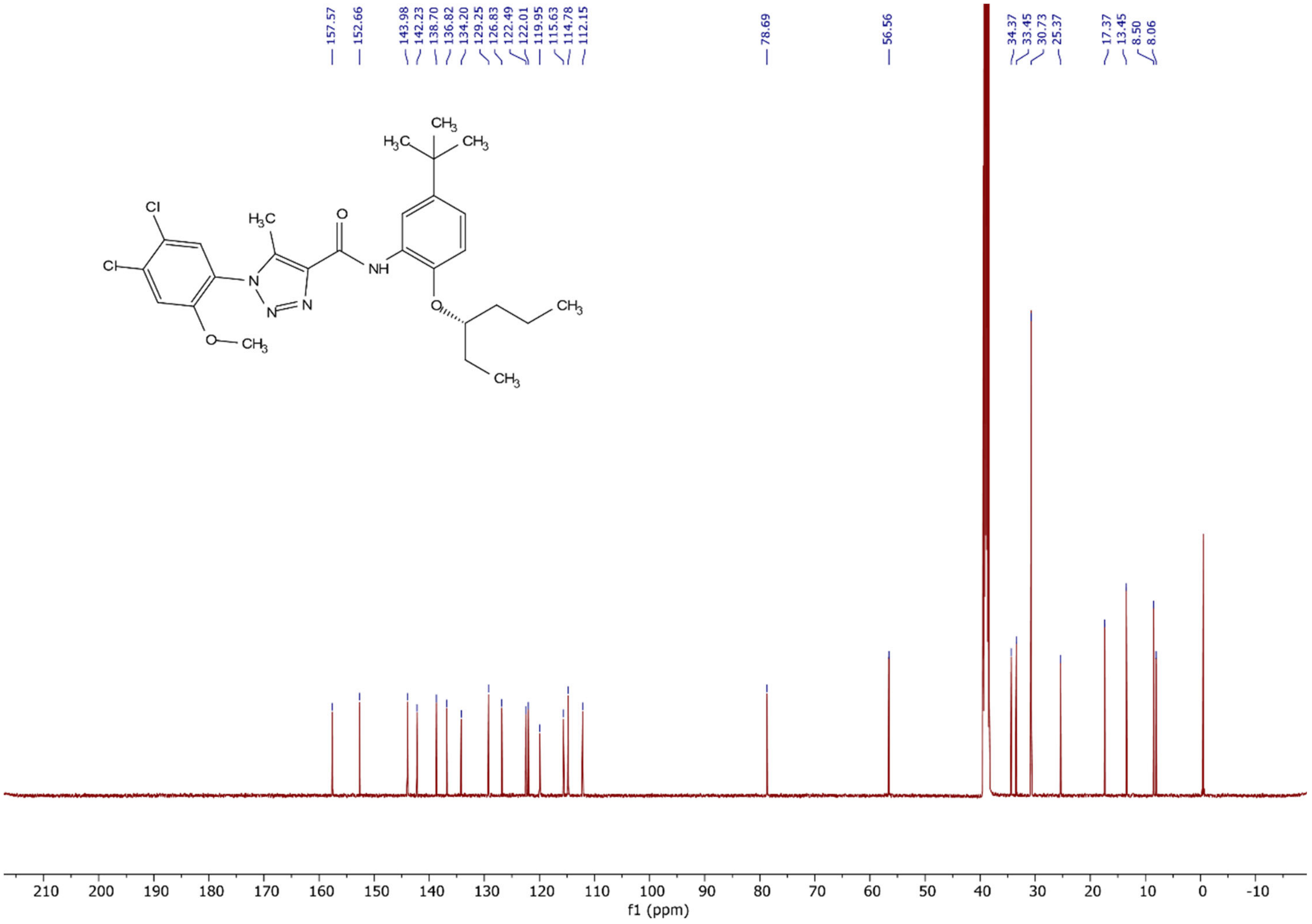

Supplementary Figure 41. ^{13}C NMR of compound SJPYT-329.

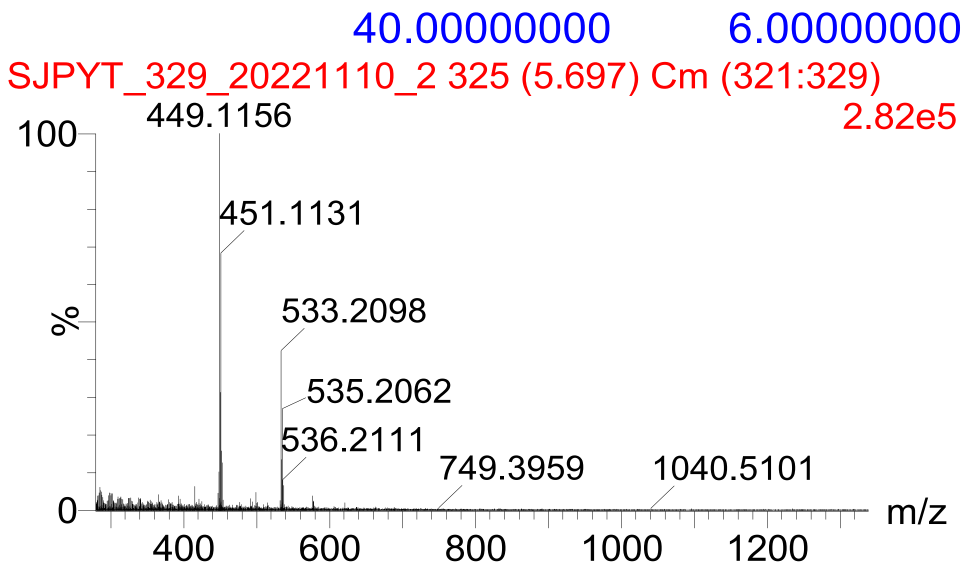

Supplementary Figure 42. HRMS of compound SJPYT-329.

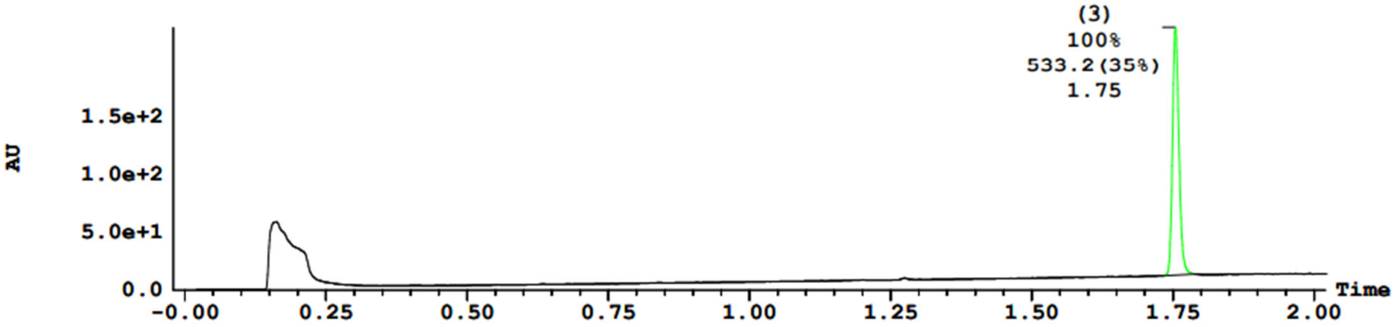

Peak Number	Compound	Time	AreaAbs	Area %Total	Width	Height	Mass Found
3	Found	1.75	3e+006	100.00	0	2e+008	533.2081

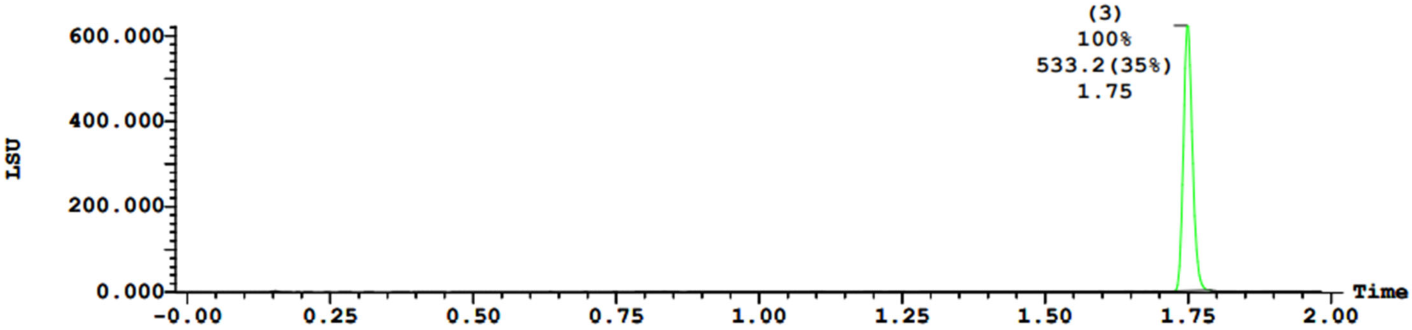

Peak Number	Compound	Time	AreaAbs	Area %Total	Width	Height	Mass Found
3	Found	1.75	1e+004	100.00	0	6e+005	533.2081

Supplementary Figure 43. HPLC of compound SJPYT-329.

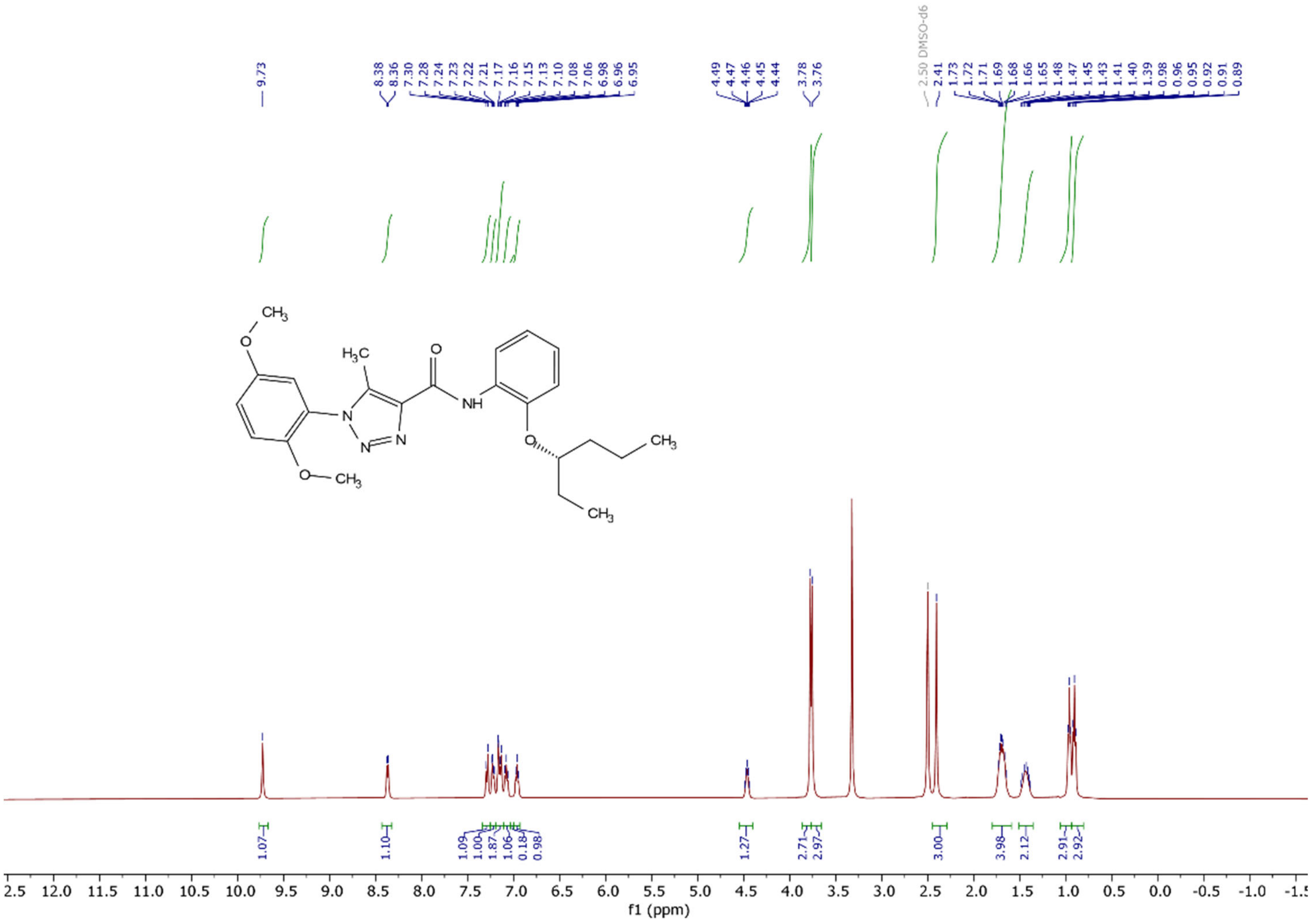

Supplementary Figure 44. ^{1}H NMR of compound SJPYT-330.

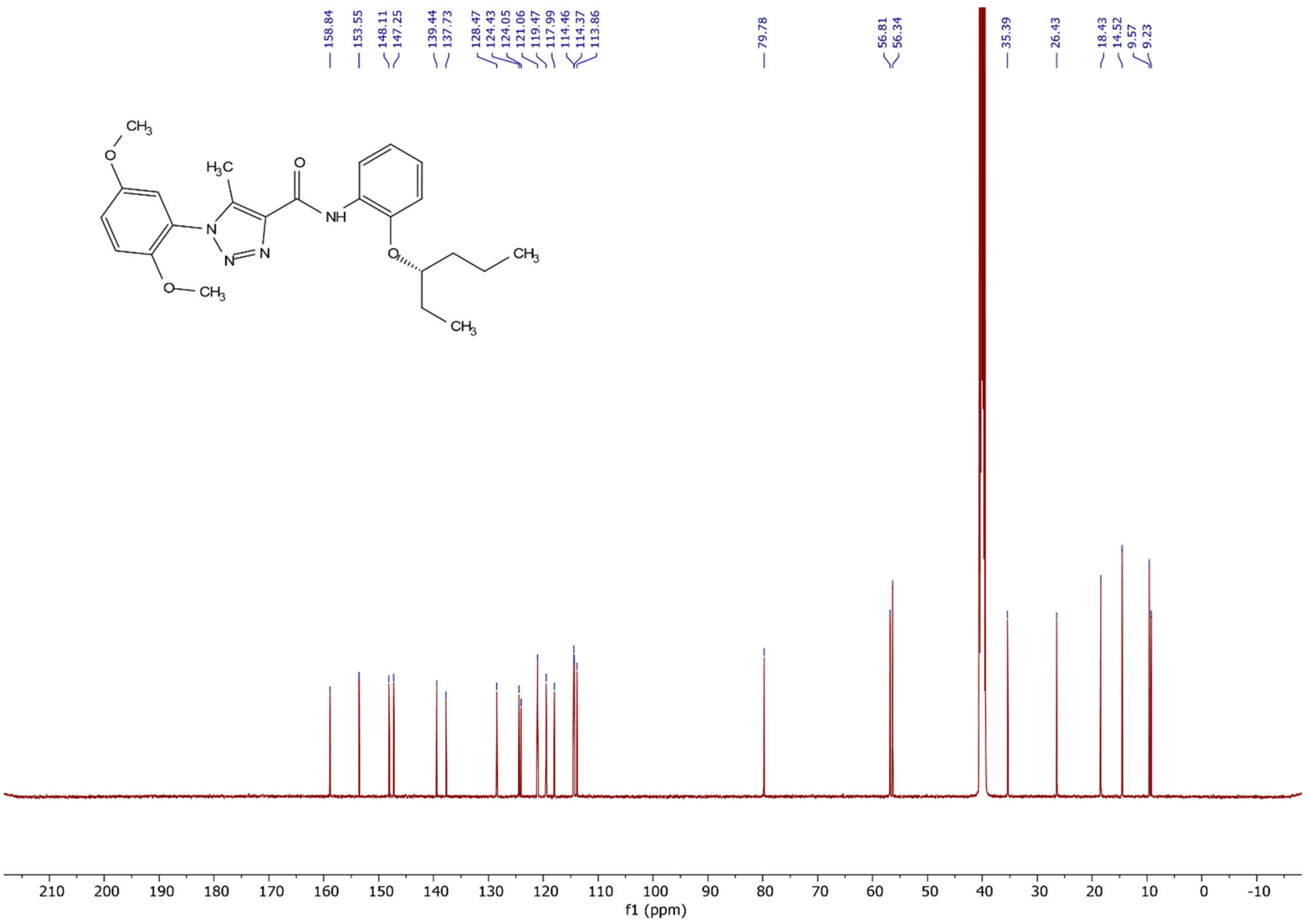

Supplementary Figure 45. ^{13}C NMR of compound SJPYT-330.

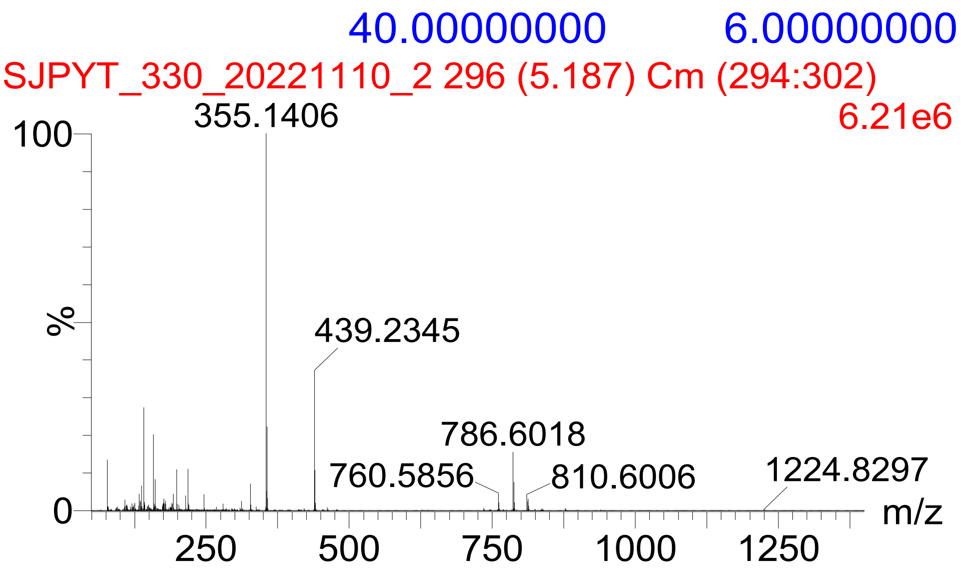

Supplementary Figure 46. HRMS of compound SJPYT-330.

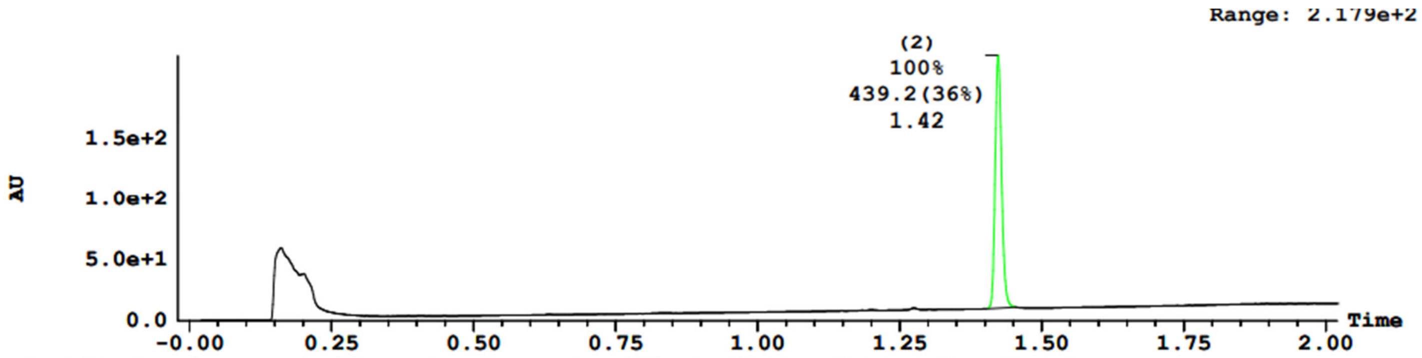

Peak Number	Compound	Time	AreaAbs	Area %Total	Width	Height	Mass Found
2	Found	1.42	3e+006	100.00	0	2e+008	439.2340

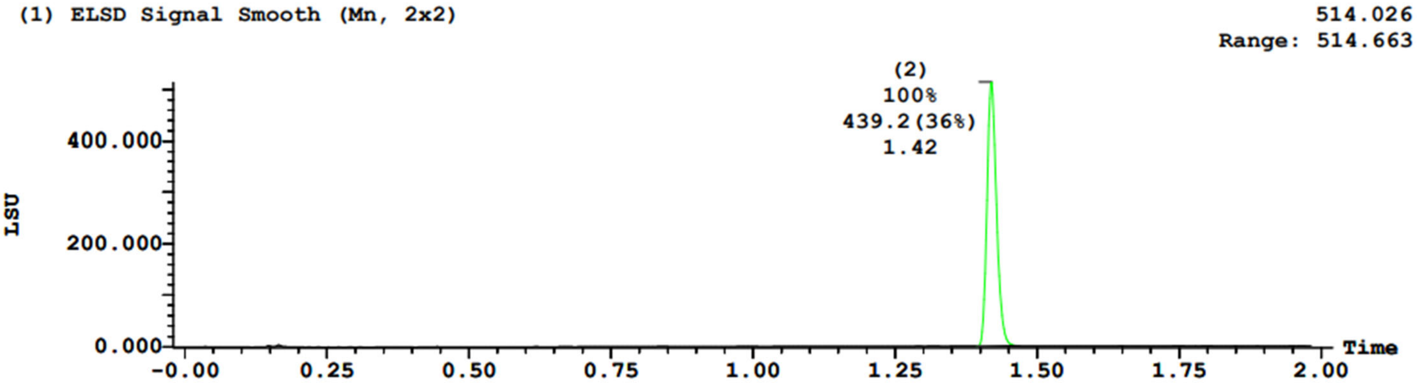

Peak Number	Compound	Time	AreaAbs	Area %Total	Width	Height	Mass Found
2	Found	1.42	1e+004	100.00	0	5e+005	439.2340

Supplementary Figure 47. HPLC of compound SJPYT-330.

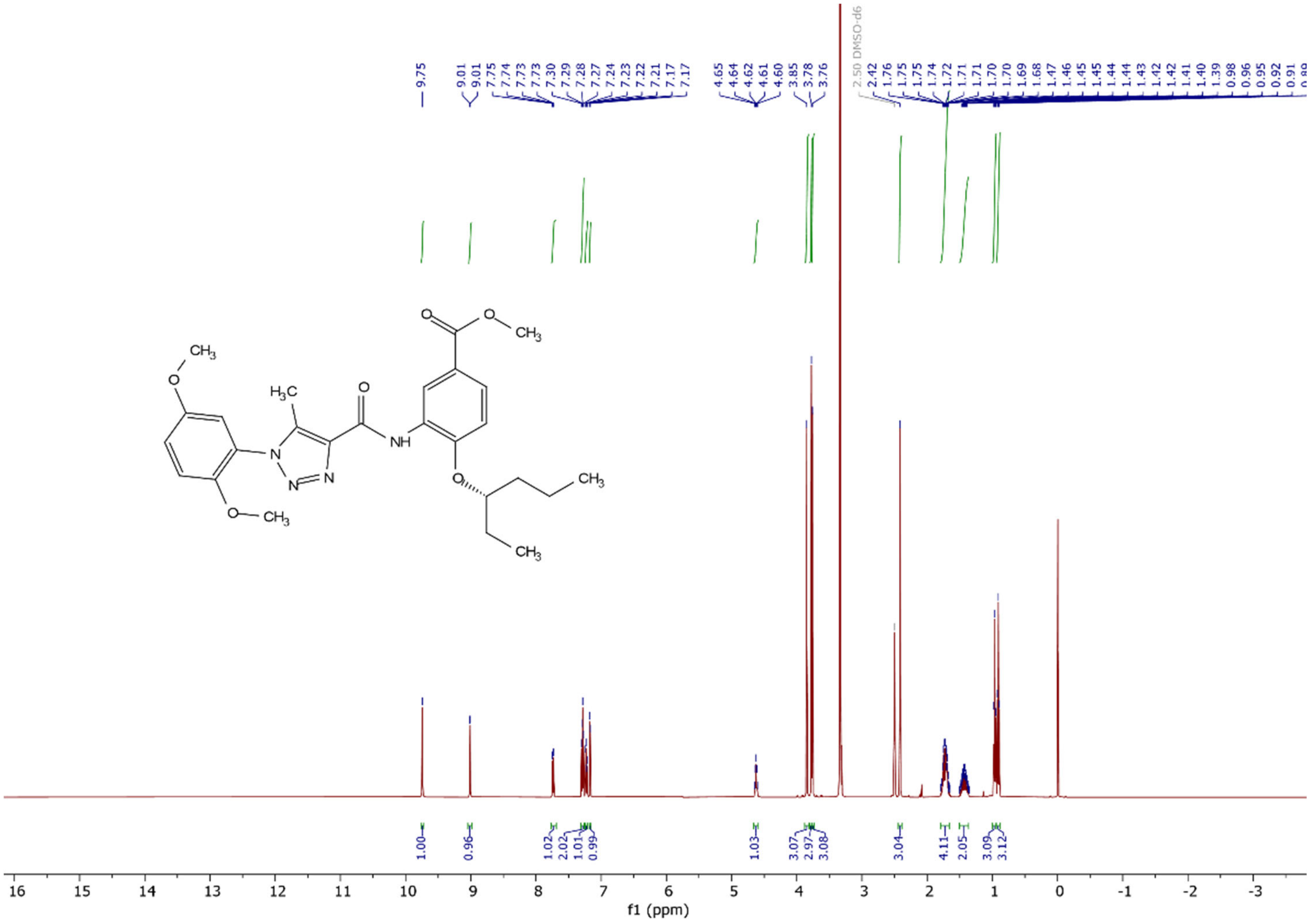

Supplementary Figure 48. ^{1}H NMR of compound SJPYT-331.

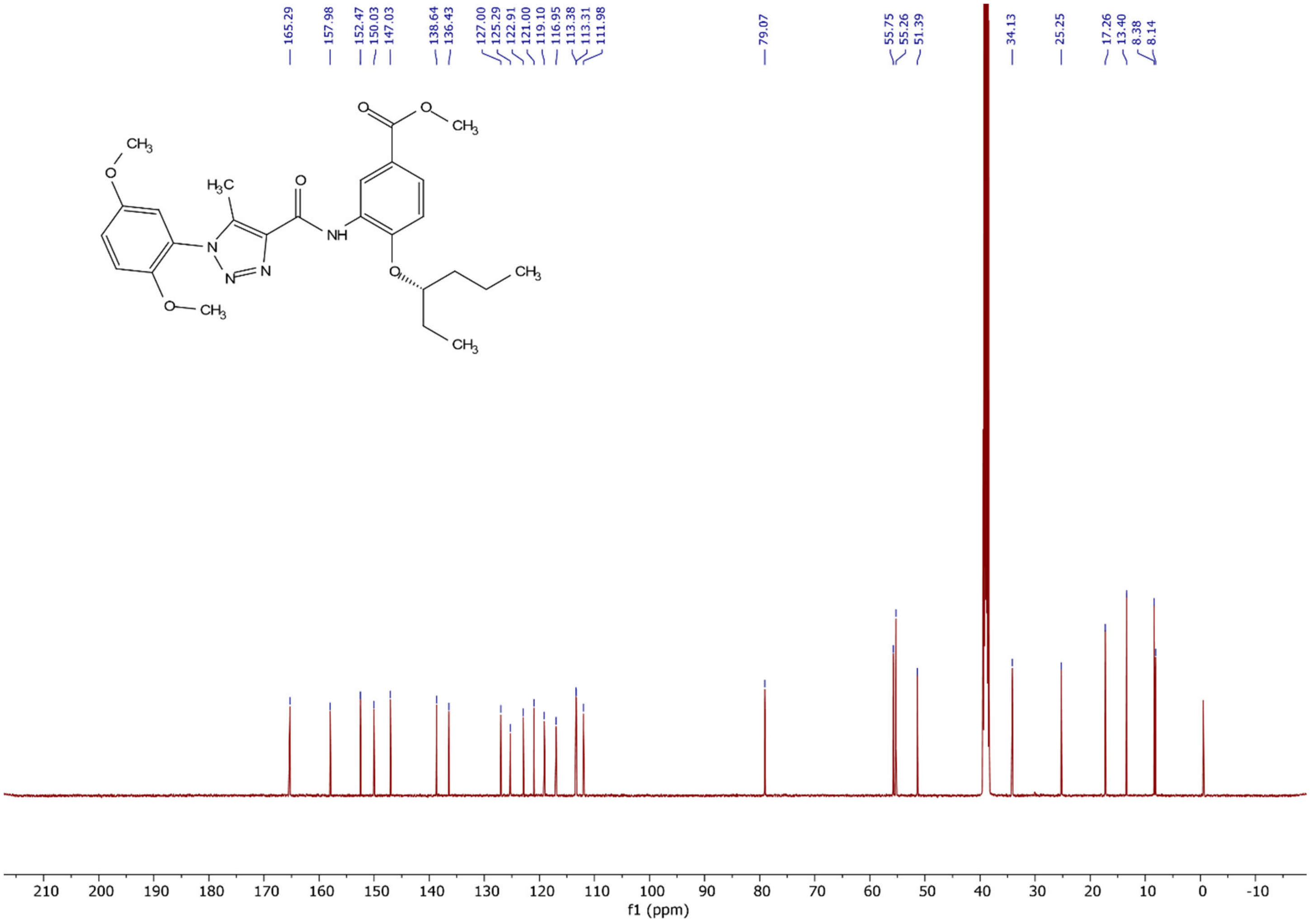

Supplementary Figure 49. ^{13}C NMR of compound SJPYT-331.

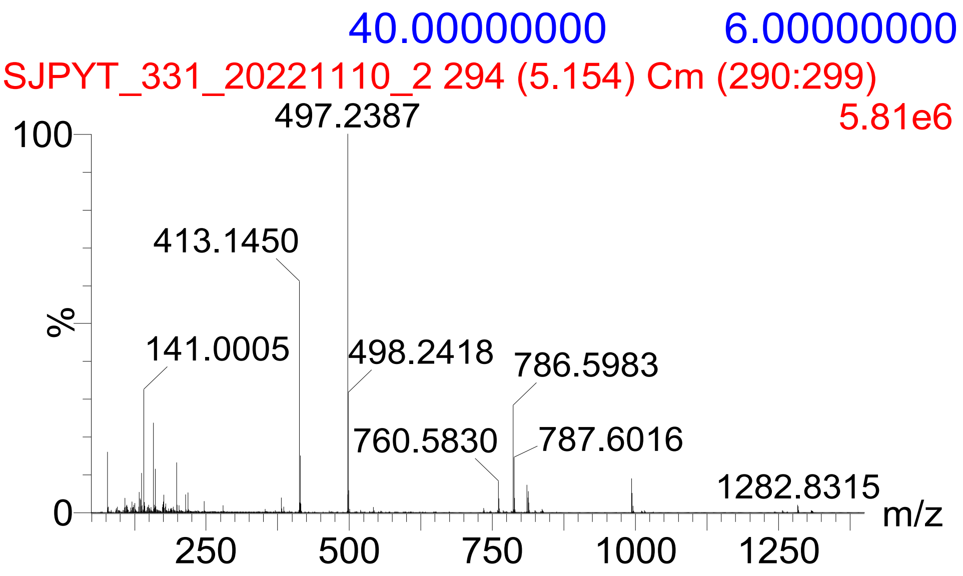

Supplementary Figure 50. HRMS of compound SJPYT-331.

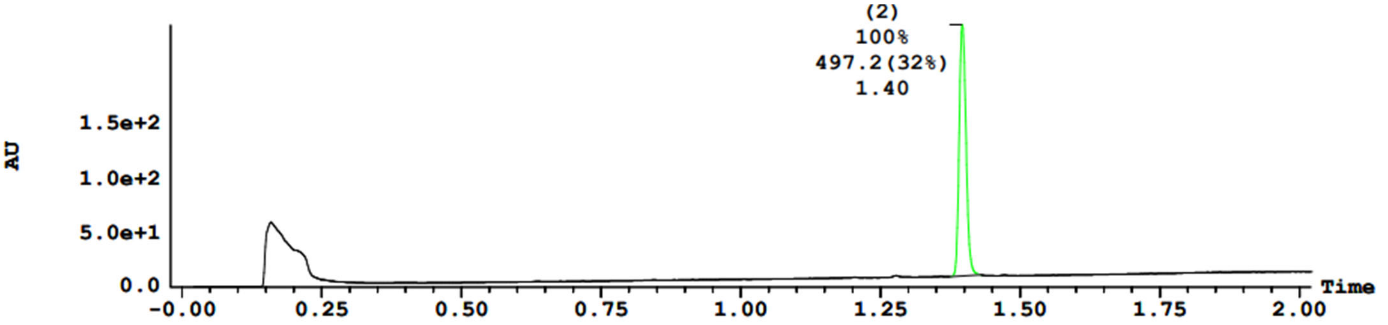

Peak Number	Compound	Time	AreaAbs	Area %Total	Width	Height	Mass Found
2	Found	1.40	3e+006	100.00	0	2e+008	497.2395

(1) ELSD Signal Smooth (Mn, 2x2) 616.186
Range: 616.877

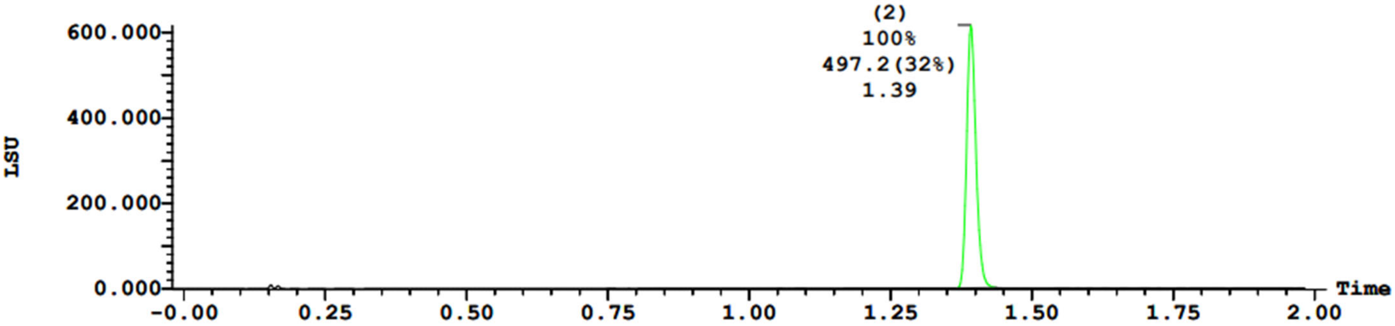

Peak Number	Compound	Time	AreaAbs	Area %Total	Width	Height	Mass Found
2	Found	1.39	1e+004	100.00	0	6e+005	497.2395

Supplementary Figure 51. HPLC of compound SJPYT-331.

Supplementary Table 1. Data collection and refinement statistics for PXR-apo, PXR-SJPYT-278, PXR-SJPYT-310, and PXR-SJPYT-312.[†]

	PXR-apo	PXR-SJPYT-278	PXR-SJPYT-310	PXR-SJPYT-312
Data collection				
Wavelength	1.000	0.920	1.000	1.000
Beamline	SER-CAT/22-ID	BNL/AMX-17-ID-1	SER-CAT/22-ID	SER-CAT/22-ID
Resolution range (Å)	89.65 – 2.20 (2.27 - 2.20)[a]	29.29 - 3.32 (3.59 - 3.32)[a]	89.20 – 2.35 (2.43 - 2.35)[a]	84.33 – 2.75 (2.88 – 2.75)[a]
Space group	$P\,2_1\,2_1\,2_1$	$P\,2_1\,2_1\,2_1$	$P\,2_1\,2_1\,2_1$	$P\,2_1\,2_1\,2_1$
Unit cell: *a*, *b*, *c* (Å) α, β, γ (˚)	83.99 89.653 106.87 90 90 90	85.04 87.35 106.27 90 90 90	84.28 89.20 105.78 90 90 90	84.33 87.90 104.53 90 90 90
Mosaicity (˚)	0.09	0.13	0.16	0.17
Total reflections	565041	63947	410147	217642
Unique reflections	41701 (4094)	12094 (2432)	33919 (3308)	20837 (2709)
Multiplicity	13.5 (14.0)	5.3 (5.4)	12.1 (12.6)	10.4 (11.0)
Completeness (%)	100.0 (100.0)	99.3 (98.5)	100.0 (100.0)	100.0 (100.0)
Mean $I/\sigma(I)$	13.6 (1.9)	9.4 (3.1)	13.9 (1.6)	12.4 (1.8)
Wilson B-factor (Å^2)	58.01	88.53	63.29	75.9
R_{merge}[b]	0.10 (1.47)	0.13 (0.53)	0.09 (1.60)	0.11 (1.33)
$CC_{1/2}$ (%)	99.8 (75.3)	99.7 (89.4)	99.8 (63.3)	99.9 (68.5)
Refinement statistics				
Resolution range (Å)	53.44 – 2.20 (2.28 – 2.20)[a]	29.29 – 3.32 (3.44 – 3.32)[a]	53.01 – 2.35 (2.43 - 2.35)[a]	67.28 – 2.75 (2.85 – 2.75)[a]
Reflections used in refinement	41639 (4088)	12068 (1163)	33861 (3305)	20790 (2040)
Reflections used for R_{free}	2133 (210)	611 (62)	1623 (149)	1000 (120)
R_{work}	0.211 (0.309)	0.219 (0.285)	0.206 (0.288)	0.230 (0.381)
R_{free}	0.235 (0.334)	0.252 (0.288)	0.238 (0.317)	0.262 (0.408)
No. of non-hydrogen atoms				
Protein	4594	4258	4525	4470
Ligand	-	144	68	72
Water	72	6	60	10
Protein residues	576	541	568	563
RMS (bonds)	0.003	0.002	0.003	0.002
RMS (angles)	0.57	0.41	0.50	0.45
Ramachandran favored (%)	98.21	97.12	97.83	98.16
Ramachandran allowed (%)	1.79	2.88	2.17	1.84
Ramachandran outliers (%)	0	0	0	0
Rotamer outliers (%)	0.80	0.20	0	0.20
Clashscore	2.31	3.75	3.45	3.02
Average B-factor (Å^2)	70.57	88.96	73.05	79.80
Protein	70.71	88.91	73.33	79.74
Ligand	-	91.86	63.02	86.05
Water	61.61	60.19	63.36	58.68
PDB ID	8SVN	8SVP	8SVO	8SVQ

[†]PXR LBD is referred to as PXR

[a]Values in parentheses for the highest resolution shell

[b]$R_{merge} = \Sigma_{hkl}\Sigma_i|I_i(hkl)-I_{av}(hkl)|/\Sigma_{hkl}\Sigma_i I_i(hkl)$

Supplementary Table 2. RMSD[a] values of apo PXR[†] structures.

PDB ID	RMSD (Å)	
	PXR-apo_A[b]	PXR-apo_B[b]
PXR-apo_A[b]	-	1.121
PXR-apo_B[b]	1.121	-
1ILG_A	0.824	1.617
7AX8_A	0.989	0.582
3CTB_A	0.513	1.032
3CTB_B	1.011	1.136
4J5W_A	1.002	1.248
4J5W_B	1.147	1.266

[†]PXR LBD is referred to as PXR

[a]The root-mean square deviation (RMSD) of Cα atoms was calculated using the “cealign” command in PyMOL after removing SRC-1 atoms

[b]Current study

Supplementary Table 3. Data collection and refinement statistics for PXR-SYPYT-326, PXR-SJPYT-328, and PXR-SJPYT-331.[†]

	PXR-SJPYT-326	PXR-SJPYT-328	PXR-SJPYT-331
Data collection			
Wavelength	1.000	1.000	0.920
Beamline	SER-CAT/22-ID	SER-CAT/22-ID	BNL/AMX-17-ID-1
Resolution range (Å)	100.00 – 2.92 (3.00 – 2.92)[a]	88.26 – 2.68 (2.81 – 2.68)[a]	29.20 – 2.39 (2.45 – 2.39)[a]
Space group	$P\ 2_1\ 2_1\ 2_1$	$P\ 2_1\ 2_1\ 2_1$	$P\ 2_1\ 2_1\ 2_1$
Unit cell: *a*, *b*, *c* (Å)<br>α, β, γ (˚)	84.08 88.50 105.38<br>90 90 90	84.47 88.26 107.26<br>90 90 90	77.20 83.25 105.88<br>90 90 90
Mosaicity (˚)	0.45	0.17	0.16
Total reflections	198103	277353	202470
Unique reflections	17445 (2407)	22874 (2764)	27402 (2548)
Multiplicity	11.4 (8.0)	12.1 (7.6)	7.4 (6.1)
Completeness (%)	99.6 (99.5)	98.7 (91.7)	99.05 (91.86)
Mean $I/\sigma(I)$	20.2 (2.0)	14.3 (2.0)	14.3 (2.2)
Wilson B-factor (Å^2)	48.23	78.04	46.73
R_{merge}[b]	0.11 (0.81)	0.09 (0.77)	0.09 (0.65)
$CC_{1/2}$ (%)	100.00 (68.8)	99.8 (64.2)	99.8 (82.0)
Refinement statistics			
Resolution range (Å)	30.81 – 2.92 (3.02 – 2.92)[a]	61.03 – 2.68 (2.77 – 2.68)[a]	28.30 – 2.39 (2.48 – 2.39)[a]
Reflections used in refinement	17161 (1362)	22800 (2036)	27351 (2505)
Reflections used for R_{free}	845 (59)	1174 (105)	1388 (121)
R_{work}	0.224 (0.350)	0.224 (0.43)	0.202 (0.285)
R_{free}	0.258 (0.284)	0.250 (0.47)	0.237 (0.397)
No. of non-hydrogen atoms			
Protein	4448	4429	4569
Ligand	64	74	72
Water	3	4	78
Protein residues	560	558	571
RMS (bonds)	0.002	0.002	0.002
RMS (angles)	0.47	0.42	0.47
Ramachandran favored (%)	97.41	97.78	97.65
Ramachandran allowed (%)	2.59	2.22	2.35
Ramachandran outliers (%)	0	0	0
Rotamer outliers (%)	0.6	0.6	0.8
Clashscore	4.41	2.38	3.39
Average B-factor (Å^2)	43.26	82.77	59.38
Protein	43.04	82.84	59.42
Ligand	58.96	79.53	63.58
Water	31.27	64.40	52.30
PDB ID	8SVR	8SVS	8SVT

[†]PXR LBD is referred to as PXR

[a]Values in parentheses for the highest resolution shell

[b]$R_{merge} = \Sigma_{hkl}\Sigma_i|I_i(hkl)-I_{av}(hkl)|/\Sigma_{hkl}\Sigma_i I_i(hkl)$

Supplementary Table 4. Data collection and refinement statistics for PXR^{L428V}-apo and PXR^{L428V}-SJPYT-331.[†]

	**PXR^{L428V}-apo**	**PXR^{L428V}-SJPYT-331**
Data collection		
Wavelength	0.920	1.000
Beamline	BNL/FMX-17-ID-2	SER-CAT/22-ID
Resolution range (Å)	29.68 - 2.89 (3.07 - 2.89)[a]	89.26 - 2.14 (2.20 - 2.14)[a]
Space group	$P\ 2_1\ 2_1\ 2_1$	$P\ 2_1\ 2_1\ 2_1$
Unit cell: *a*, *b*, *c* (Å) α, β, γ (˚)	85.28 89.78 106.12 90 90 90	85.04 89.26 105.71 90 90 90
Mosaicity (˚)	0.15	0.11
Total reflections	124670	502346
Unique reflections	18765 (2923)	43922 (3029)
Multiplicity	6.6 (6.6)	11.4 (5.4)
Completeness (%)	99.6 (98.1)	97.4 (83.3)
Mean $I/\sigma(I)$	13.1 (2.2)	19.6 (1.6)
Wilson B-factor (Å^2)	75.39	53.02
R_{merge}[b]	0.09 (0.82)	0.06 (0.56)
$CC_{1/2}$ (%)	99.9 (71.9)	99.9 (75.9)
Refinement statistics		
Resolution range (Å)	28.80 - 2.89 (2.99 - 2.89)[a]	44.63 - 2.14 (2.22 - 2.14)[a]
Reflections used in refinement	18721 (1778)	43858 (3711)
Reflections used for R_{free}	886 (97)	2212 (174)
R_{work}	0.204 (0.267)	0.207 (0.348)
R_{free}	0.244 (0.329)	0.235 (0.352)
Number of non-hydrogen atoms		
Protein	4404	4546
Ligand	-	72
Water	5	88
Protein residues	562	574
RMS (bonds)	0.004	0.002
RMS (angles)	0.55	0.42
Ramachandran favored (%)	97.61	98.03
Ramachandran allowed (%)	2.39	1.97
Ramachandran outliers (%)	0	0
Rotamer outliers (%)	0	0.4
Clashscore	3.41	1.76
Average B-factor (Å^2)	82.22	64.27
Protein	82.24	64.43
Ligand	-	65.11
Water	58.42	55.09
PDB ID	8SVU	8SVX

[†]PXR^{L428V} LBD is referred to as PXR^{L428V}

[a]Values in parentheses for the highest resolution shell

[b]$R_{merge} = \Sigma_{hkl}\Sigma_i|I_i(hkl)-I_{av}(hkl)|/\Sigma_{hkl}\Sigma_i I_i(hkl)$

Supplementary Table 5. Comparison of RMSD[a] values of SJPYT-331-bound PXR^{L428V} with PXR^{L428V}-apo and SJPYT-331-bound WT PXR structures.

Crystal structures†	RMSD (Å)	
	PXR^{L428V}-SJPYT-331_A	PXR^{L428V}-SJPYT-331_B
PXR^{L428V}-apo_A	0.847	1.420
PXR^{L428V}-apo_B	0.746	0.735
PXR-SJPYT-331_A	1.021	0.894
PXR-SJPYT-331_B	1.222	1.485

†PXR LBD and PXR^{L428V} LBD are referred to as PXR and PXR^{L428V}, respectively

[a]The root-mean square deviation (RMSD) of Cα atoms was calculated using the "cealign" command in PyMOL after removing SRC-1 atoms

Supplementary Methods

General methods and synthesis. Organic reagents were purchased from commercial suppliers unless otherwise noted and were used without further purification. All solvents were analytical or reagent grade and the solvents were dried using the Glass Contour Solvent Systems by SG Water USA. All reactions with water- and/or air-sensitive starting materials were carried out in pre-dried glassware under argon atmosphere with standard procedure. Flash column chromatography was performed by using Biotage Isolera Flash Systems and Biotage SNAP Ultra or Biotage SNAP Ultra C18 columns. All reactions as well as compound purities were monitored by UPLC-MS by using a Waters Acquity UPLC MS system with a C18 column in a 2-min gradient [H_2O + 0.1% formic acid (FA) → acetonitrile (ACN) + 0.1% FA] and detectors of PDA (215–400 nm), ELSD, and Acquity SQD ESI-positive MS (Waters Corporation, Milford, MA). High-resolution mass spectra were determined by using a Waters Acquity UPLC system with a C18 column (H_2O + 0.1% FA → ACN + 0.1% FA gradient over 2.5 min) and Xevo G2Q-TOF ESI-positive MS in resolution mode. Compounds were internally normalized to leucine-enkephalin lock solution, with a calculated error of <3 ppm. All final compounds used for SAR studies have purity at 95% or greater by HPLC. All NMR spectra were recorded on Bruker 500 MHz spectrometer in the solvents as indicated, and spectra were processed using MestReNova (14.1.0). The chemical shift values are expressed in parts per million (ppm) relative to tetramethylsilane as the internal standard. Coupling constants (*J*) are reported in hertz (Hz).

The synthesis of SPA70 amide derivatives is illustrated in Supplementary Figures 13–14. In Supplementary Figure 13, compound **1** was coupled with the corresponding aniline in the presence of EDCI and HOBt to give compounds **2-4**.[1] The intermediate compounds **2** was then subjected to S_N2 nucleophilic substitutions with the corresponding alkyl halides to give compounds **SJPYT-278**.[2] Compounds **2-4** were coupled with the respective (*R*)-pentan-2-ol or (*S*)-pentan-2-ol to generate the corresponding compounds **SJPYT-312**, **SJPYT-313**, **SJPYT-330** and **SJPYT-331** via a Mitsunobu reaction.[3] In Supplementary Figure 14, 4-(*tert*-butyl)-2-nitrophenol (**5**) was coupled with the (*R*)-pentan-2-ol to generate (S)-4-(*tert*-butyl)-1-(hexan-3-yloxy)-2-nitrobenzene (**6**) via a Mitsunobu reaction.[3] Then the nitro group of (*S*)-4-(*tert*-butyl)-1-(hexan-3-yloxy)-2-nitrobenzene (**6**) was reduced by using Pd/C and $HCOONH_4$ in MeOH to generate (*S*)-5-(*tert*-butyl)-2-(hexan-3-yloxy)aniline (**7**).[4] Substituted aniline compounds **8a–d** were first converted to the corresponding triazole compounds **9a–d** through Wolff 1,2,3-triazole synthesis.[5,6] Then, compounds **9a–d** were hydrolyzed by LiOH to generate the corresponding acid.[7] Finally, the corresponding acid were coupled with the (*S*)-5-(*tert*-butyl)-2-(hexan-3-yloxy)aniline (**7**) to generate the corresponding compounds **SJPYT-326**, **327**, **328** and **329**. Characterization of all compounds is shown in Supplementary Figures 15-51.

N-(5-(*tert*-Butyl)-2-(hexan-3-yloxy)phenyl)-1-(2,5-dimethoxyphenyl)-5-methyl-1*H*-1,2,3-triazole-4-carboxamide (**SJPYT-278**). 2-amino-4-(*tert*-butyl)phenol (0.165 g, 1.000 mmol) was added to a solution of 1-(2,5-dimethoxyphenyl)-5-methyl-1*H*-1,2,3-triazole-4-carboxylic acid (**1**, 0.263 g, 1 mmol), EDCI (0.288 g, 1.5 mmol), HOBt (wetted with not less than 20% by weight of water, 0.203 g, 1.2 mmol), and DIEA (0.258 g, 2.000 mmol) in DMF (5 mL). The resulting mixture was stirred at room temperature overnight, then it was diluted with water (50 mL) and extracted with EtOAc (50 mL × 2). The combined organic phase was washed with saturated aq. $NaHCO_3$, water, and brine, then dried with $MgSO_4$ and concentrated. The residue was purified by flash chromatography (0% to 100% EtOAc in hexane) to give compound **2** as a white solid (181.8 mg, 56% yield). ^1H NMR (400 MHz, DMSO-d_6) δ 10.01 (s, 1H), 9.61 (s, 1H), 8.33 (d, *J* = 2.4 Hz, 1H), 7.29 (d, *J* = 9.2 Hz, 1H), 7.22 (dd, *J* = 9.1, 3.0 Hz, 1H), 7.16 (d, *J* = 3.0 Hz, 1H), 6.99 (dd, *J* = 8.4, 2.4 Hz, 1H), 6.86 (d, *J* = 8.5 Hz, 1H), 3.78 (s, 3H), 3.76 (s, 3H), 2.41 (s, 3H), 1.27 (s, 9H). ^{13}C NMR (101 MHz, DMSO-d_6) δ 158.45, 153.06, 147.65, 144.15, 141.51, 138.80, 137.30, 125.64, 123.61, 120.65, 117.44, 116.80, 114.28, 113.98, 113.89, 56.32, 55.85, 33.91, 31.39, 8.72.

To a solution of *N*-(5-(*tert*-butyl)-2-hydroxyphenyl)-1-(2,5-dimethoxyphenyl)-5-methyl-1*H*-1,2,3-triazole-4-carboxamide (**2**, 100 mg, 0.244 mmol) in DMF (5 mL) Cs_2CO_3 (175 mg, 0.536 mmol) and 3-bromohexane (48.3 mg, 0.292 mmol) were added. The suspension was stirred at 60 ℃ for overnight. The reaction mixture was then diluted with water (50 mL) and extracted with EtOAc (50 mL × 2). The EtOAc layer was washed with water, dried with anhydrous Na_2SO_4, and concentrated. The residue was purified by silica gel chromatography (0% to 100% EA in hexane) to give product **SJPYT-278** as a white solid (95.3 mg, 79% yield, 99.24% purity). ^1H NMR (500 MHz, $CDCl_3$) δ 9.88 (s, 1H), 8.66 (d, *J* = 2.4 Hz, 1H), 7.08 (dd, *J* = 9.1, 3.0 Hz, 1H), 7.07 – 7.01 (m, 2H), 6.97 (d, *J* = 3.0 Hz, 1H), 6.84 (d, *J* = 8.6 Hz, 1H), 4.27 (p, *J* = 5.8 Hz, 1H), 3.82 (s, 3H), 3.76 (s, 3H), 2.54 (s, 3H), 1.82 – 1.63 (m, 4H), 1.53 – 1.42 (m, 2H), 1.36 (s, 9H), 1.01 (t, *J* = 7.5 Hz, 3H), 0.94 (t, *J* = 7.4 Hz, 3H). ^{13}C NMR (126 MHz, $CDCl_3$) δ 159.42, 153.68, 148.01, 145.03, 143.60, 139.04, 138.56, 128.36, 124.53, 120.05, 117.46,

117.01, 113.71, 113.30, 112.03, 80.27, 56.34, 56.01, 35.51, 34.45, 31.60, 26.57, 18.69, 14.23, 9.58, 9.32. ESI-TOF HRMS: *m/z* 495.2967 ($C_{28}H_{38}N_4O_4$ + H^+ requires 495.2966).

(*S*)-*N*-(5-(*tert*-butyl)-2-(hexan-3-yloxy)phenyl)-1-(2,5-dimethoxyphenyl)-5-methyl-1*H*-1,2,3-triazole-4-carboxamide (**SJPYT-312**). *N*-(5-(tert-butyl)-2-hydroxyphenyl)-1-(2,5-dimethoxyphenyl)-5-methyl-1*H*-1,2,3-triazole-4-carboxamide (**2**, 100 mg, 0.244 mmol), (*R*)-hexan-3-ol (45.6 µl, 0.365 mmol), and PPh_3 (83 mg, 0.317 mmol) were added to dry DCM (10 mL) in a dried 25-mL round-bottom flask charged with a stir bar under an inert atmosphere. To the solution was added diisopropyl diazene-1,2-dicarboxylate (DIAD, 72.0 µl, 0.365 mmol). The reaction mixture was stirred at room temperature overnight. Then the reaction mixture was quenched by adding sodium hydroxide solution (0.5 N, 10 mL) and extracted with EtOAc (25 mL × 2). The combined organic layers were washed with water and brine, dried over anhydrous $MgSO_4$, and concentrated. The residue was purified by silica gel chromatography (0% to 100% EtOAc in hexane) to give product (*S*)-N-(5-(*tert*-butyl)-2-(hexan-3-yloxy)phenyl)-1-(2,5-dimethoxyphenyl)-5-methyl-1*H*-1,2,3-triazole-4-carboxamide as a white solid (83.2 mg, 71% yield, 99.57% purity, %ee = 100%). ^1H NMR (500 MHz, DMSO-d_6) δ 9.70 (s, 1H), 8.50 (d, *J* = 2.4 Hz, 1H), 7.29 (d, *J* = 9.2 Hz, 1H), 7.22 (dd, *J* = 9.2, 3.1 Hz, 1H), 7.17 (d, *J* = 3.0 Hz, 1H), 7.08 (dd, *J* = 8.6, 2.4 Hz, 1H), 7.03 (d, *J* = 8.7 Hz, 1H), 4.40 (p, *J* = 5.7 Hz, 1H), 3.78 (s, 3H), 3.76 (s, 3H), 2.41 (s, 3H), 1.74 – 1.60 (m, 4H), 1.48 – 1.38 (m, 2H), 1.29 (s, 9H), 0.95 (t, *J* = 7.4 Hz, 3H), 0.91 (t, *J* = 7.4 Hz, 3H). ^{13}C NMR (126 MHz, DMSO-d_6) δ 158.84, 153.55, 148.10, 145.02, 143.31, 139.38, 137.80, 127.99, 124.06, 120.93, 117.99, 116.66, 114.43, 114.37, 113.22, 79.76, 56.82, 56.34, 35.45, 34.53, 31.81, 26.47, 18.47, 14.53, 9.59, 9.24. ESI-TOF HRMS: *m/z* 495.2956 ($C_{28}H_{38}N_4O_4$ + H^+ requires 495.2966).

(*R*)-*N*-(5-(*tert*-butyl)-2-(hexan-3-yloxy)phenyl)-1-(2,5-dimethoxyphenyl)-5-methyl-1*H*-1,2,3-triazole-4-carboxamide (**SJPYT-313**). This compound was synthesized by using a procedure similar to that described for compound **SJPYT-312**, employing compound **2** and (*S*)-hexan-3-ol, to give a white solid (98.8 mg, 82% yield, 100% purity, %ee = 100%). ^1H NMR (500 MHz, DMSO-d_6) δ 9.70 (s, 1H), 8.50 (d, *J* = 2.4 Hz, 1H), 7.29 (d, *J* = 9.2 Hz, 1H), 7.22 (dd, *J* = 9.2, 3.1 Hz, 1H), 7.17 (d, *J* = 3.0 Hz, 1H), 7.08 (dd, *J* = 8.7, 2.4 Hz, 1H), 7.03 (d, *J* = 8.7 Hz, 1H), 4.40 (p, *J* = 5.8 Hz, 1H), 3.78 (s, 3H), 3.76 (s, 3H), 2.41 (s, 3H), 1.78 – 1.60 (m, 4H), 1.50 – 1.38 (m, 2H), 1.29 (s, 9H), 0.95 (t, *J* = 7.4 Hz, 3H), 0.91 (t, *J* = 7.4 Hz, 3H). ^{13}C NMR (126 MHz, DMSO-d_6) δ 158.84, 153.55, 148.10, 145.02, 143.31, 139.38, 137.80, 127.99, 124.06, 120.93, 117.99, 116.66, 114.43, 114.37, 113.22, 79.76, 56.82, 56.34, 35.45, 34.53, 31.81, 26.47, 18.47, 14.53, 9.59, 9.24. ESI-TOF HRMS: *m/z* 495.2980 ($C_{28}H_{38}N_4O_4$ + H^+ requires 495.2966).

(*S*)-1-(2,5-Dimethoxyphenyl)-*N*-(2-(hexan-3-yloxy)phenyl)-5-methyl-1*H*-1,2,3-triazole-4-carboxamide (**SJPYT-330**). Compound **3** was synthesized by using a procedure similar to that described for compound **2**, employing compound **1** and 2-aminophenol, to give a white solid (369.2 mg, 55% yield). ^1H NMR (500 MHz, DMSO-d_6) δ 10.27 (s, 1H), 9.61 (s, 1H), 8.22 (dd, *J* = 8.0, 1.4 Hz, 1H), 7.29 (d, *J* = 9.2 Hz, 1H), 7.22 (dd, *J* = 9.1, 3.0 Hz, 1H), 7.15 (d, *J* = 3.0 Hz, 1H), 7.01 – 6.90 (m, 2H), 6.84 (ddd, *J* = 8.5, 6.8, 2.1 Hz, 1H), 3.78 (s, 3H), 3.75 (s, 3H), 2.40 (s, 3H). ^{13}C NMR (126 MHz, DMSO-d_6) δ 157.85, 152.47, 147.08, 145.89, 138.28, 136.66, 125.56, 123.51, 123.00, 119.07, 118.65, 116.87, 114.22, 113.41, 113.30, 55.74, 55.27, 8.14.

Compound **SJPYT-330** was synthesized by using a procedure similar to that described for compound **SJPYT-312**, employing compound **3** and (*R*)-hexan-3-ol, to give a white solid (140.2 mg, 71% yield, 100% purity). ^1H NMR (500 MHz, DMSO-d_6) δ 9.73 (s, 1H), 8.37 (d, *J* = 8.0 Hz, 1H), 7.29 (d, *J* = 9.1 Hz, 1H), 7.22 (dd, *J* = 9.3, 3.0 Hz, 1H), 7.18 – 7.12 (m, 2H), 7.08 (t, *J* = 7.9 Hz, 1H), 6.96 (t, *J* = 7.7 Hz, 1H), 4.46 (p, *J* = 5.7 Hz, 1H), 3.78 (s, 3H), 3.76 (s, 3H), 2.41 (s, 3H), 1.69 (tt, *J* = 14.6, 6.9 Hz, 4H), 1.43 (dp, *J* = 14.3, 6.9 Hz, 2H), 0.96 (t, *J* = 7.4 Hz, 3H), 0.91 (t, *J* = 7.4 Hz, 3H). ^{13}C NMR (126 MHz, DMSO-d_6) δ 158.84, 153.55, 148.11, 147.25, 139.44, 137.73, 128.47, 124.43, 124.05, 121.06, 119.47, 117.99, 114.46, 114.37, 113.86, 79.78, 56.81, 56.34, 35.39, 26.43, 18.43, 14.52, 9.57, 9.23. ESI-TOF HRMS: *m/z* 439.2345 ($C_{24}H_{30}N_4O_4$ + H^+ requires 439.2340).

Methyl (*S*)-3-(1-(2,5-Dimethoxyphenyl)-5-methyl-1*H*-1,2,3-triazole-4-carboxamido)-4-(hexan-3-yloxy)benzoate (**SJPYT-331**). Compound **4** was synthesized by using a procedure similar to that described for compound **2**, employing compound **1** and methyl 3-amino-4-hydroxybenzoate, to give a white solid (2.34 g, 50% yield). ^1H NMR (500 MHz, DMSO-d_6) δ 11.35 (s, 1H), 9.63 (s, 1H), 8.91 (d, *J* = 2.2 Hz, 1H), 7.65 (dd, *J* = 8.4, 2.2 Hz, 1H), 7.29 (d, *J* = 9.2 Hz, 1H), 7.22 (dd, *J* = 9.2, 3.1 Hz, 1H), 7.16 (d, *J* = 3.1 Hz, 1H), 7.04 (d, *J* = 8.4 Hz, 1H), 3.82 (s, 3H), 3.78 (s, 3H), 3.76 (s, 3H), 2.42 (s, 3H). ^{13}C NMR (126 MHz, DMSO-d_6) δ 165.49, 158.02, 152.49, 150.60, 147.09, 138.53, 136.48, 125.58, 125.47, 122.97, 119.94, 119.76, 116.92, 113.88, 113.43, 113.32, 55.76, 55.28, 51.21, 8.15.

Compound **SJPYT-331** was synthesized by using a procedure similar to that described for compound **SJPYT-312**, employing compound **4** and (*R*)-hexan-3-ol, to give a white solid (113.0 mg, 94% yield, 100% purity). ^{1}H NMR (500 MHz, DMSO-d_6) δ 9.75 (s, 1H), 9.01 (d, *J* = 2.1 Hz, 1H), 7.74 (dd, *J* = 8.6, 2.2 Hz, 1H), 7.31 – 7.26 (m, 2H), 7.23 (dd, *J* = 9.1, 3.1 Hz, 1H), 7.17 (d, *J* = 3.0 Hz, 1H), 4.62 (p, *J* = 5.7 Hz, 1H), 3.85 (s, 3H), 3.78 (s, 3H), 3.76 (s, 3H), 2.42 (s, 3H), 1.82 – 1.61 (m, 4H), 1.54 – 1.34 (m, 2H), 0.96 (t, *J* = 7.4 Hz, 3H), 0.91 (t, *J* = 7.3 Hz, 3H). ^{13}C NMR (126 MHz, DMSO-d_6) δ 165.29, 157.98, 152.47, 150.03, 147.03, 138.64, 136.43, 127.00, 125.29, 122.91, 121.00, 119.10, 116.95, 113.38, 113.31, 111.98, 79.07, 55.75, 55.26, 51.39, 34.13, 25.25, 17.26, 13.40, 8.38, 8.14. ESI-TOF HRMS: *m*/*z* 497.2387 ($C_{26}H_{32}N_4O_6 + H^+$ requires 497.2395).

(*S*)-4-(*tert*-Butyl)-1-(hexan-3-yloxy)-2-nitrobenzene (**6**). Compound **6** was synthesized by using a procedure similar to that described for compound **SJPYT-312**, employing 4-(*tert*-butyl)-2-nitrophenol and (*R*)-hexan-3-ol, to give a yellow oil (2.93 g, 94% yield). ^{1}H NMR (500 MHz, DMSO-d_6) δ 7.73 (d, *J* = 2.5 Hz, 1H), 7.60 (dd, *J* = 8.9, 2.5 Hz, 1H), 7.27 (d, *J* = 8.9 Hz, 1H), 4.49 (p, *J* = 5.8 Hz, 1H), 1.72 – 1.48 (m, 4H), 1.43 – 1.27 (m, 2H), 1.27 (s, 9H), 0.94 – 0.81 (m, 6H). ^{13}C NMR (126 MHz, DMSO-d_6) δ 149.31, 143.68, 141.00, 131.68, 122.05, 116.29, 80.49, 35.69, 34.93, 31.73, 26.75, 18.71, 14.82, 9.88.

(*S*)-5-(*tert*-Butyl)-2-(hexan-3-yloxy)aniline (**7**). 10% Pd/C (0.6 g) was added to a solution of (S)-4-(*tert*-butyl)-1-(hexan-3-yloxy)-2-nitrobenzene (2.93 g, 10.49 mmol) and ammonium formate (3.31 g, 52.4 mmol) in ethanol (75 mL). The reaction mixture was heated at reflux for 12 h. Then the reaction mixture was cooled to 25 °C, and filtered through a plug of Celite. The Celite was then washed with methanol (20 mL) and the filtrate concentrated. The residue was added to saturated $NaHCO_3$ - water solution (100 mL), and extracted with EtOAc (100 mL × 2). The combined organic layers were washed with water and brine, dried over anhydrous $MgSO_4$, and concentrated. The residue was purified by silica gel chromatography (0% to 100% EtOAc in hexane) to give product (*S*)-5-(*tert*-butyl)-2-(hexan-3-yloxy)aniline as a red oil (1.13 g, 43% yield). ^{1}H NMR (500 MHz, DMSO-d_6) δ 6.74 – 6.60 (m, 2H), 6.54 – 6.45 (m, 1H), 4.47 (s, 2H), 4.11 (p, J = 5.8 Hz, 1H), 1.64 – 1.47 (m, 4H), 1.46 – 1.29 (m, 2H), 1.20 (s, 9H), 0.99 – 0.74 (m, 6H). ^{13}C NMR (126 MHz, DMSO-d_6) δ 143.29, 143.25, 138.20, 113.31, 113.23, 112.17, 78.84, 35.73, 34.12, 31.90, 26.59, 18.62, 14.59, 9.85.

Ethyl 5-Methyl-1-(2,4,5-trimethoxyphenyl)-1*H*-1,2,3-triazole-4-carboxylate (**9a**). To a solution of 2,4,5-trimethoxyaniline (0.630 g, 3.44 mmol) and ethyl 2-diazo-3-oxobutanoate (500 μL, 3.44 mmol) in toluene (5 mL) and THF (3 mL) was added titanium(IV) chloride (1.0 M solution in toluene, 5.16 mL, 5.16 mmol). The mixture was warmed to 75 °C and stirred for overnight. The reaction mixture was treated with 50 mL water and extracted with EA (2 × 50 mL). The combined organic layer was washed with brine (50 mL), dried over $MgSO_4$ and filtered. The filtrate was concentrated in vacuum, and then purified by silica gel column chromatography (0% to 100% EtOAc in hexane) to give product as a yellow solid (210.3 mg, 19% yield). ^{1}H NMR (500 MHz, DMSO-d_6) δ 7.09 (s, 1H), 6.95 (s, 1H), 4.34 (q, *J* = 7.1 Hz, 2H), 3.90 (s, 3H), 3.77 (s, 3H), 3.73 (s, 3H), 2.31 (s, 3H), 1.33 (t, *J* = 7.1 Hz, 3H). ^{13}C NMR (126 MHz, DMSO-d_6) δ 160.53, 150.81, 147.65, 141.94, 140.17, 134.35, 113.81, 111.45, 97.76, 59.76, 55.96, 55.73, 55.49, 13.60, 8.57.

Ethyl 1-(4-Fluoro-2,5-dimethoxyphenyl)-5-methyl-1*H*-1,2,3-triazole-4-carboxylate (**9b**). Compound **9b** was synthesized by using a procedure similar to that described for compound **9a**, employing 4-fluoro-2,5-dimethoxyaniline and ethyl 2-diazo-3-oxobutanoate, to give a white solid (315.3 mg, 30% yield). ^{1}H NMR (500 MHz, DMSO-d_6) δ 7.42 (d, *J* = 5.6 Hz, 1H), 7.39 (d, *J* = 9.6 Hz, 1H), 4.35 (q, *J* = 7.1 Hz, 2H), 3.82 (s, 3H), 3.75 (s, 3H), 2.32 (s, 3H), 1.33 (t, *J* = 7.1 Hz, 3H). ^{13}C NMR (126 MHz, DMSO-d_6) δ 160.43, 153.32, 151.34, 147.49, 147.42, 140.33, 140.24, 140.22, 134.48, 118.03, 118.00, 113.87, 113.84, 102.06, 101.88, 59.84, 56.24, 56.23, 13.59, 8.50.

Ethyl 1-(2,4-Dimethoxy-5-methylphenyl)-5-methyl-1*H*-1,2,3-triazole-4-carboxylate (**9c**). Compound **9c** was synthesized by using a procedure similar to that described for compound **9a**, employing 2,4-dimethoxy-5-methylaniline and ethyl 2-diazo-3-oxobutanoate, to give a white solid (372.1 mg, 35% yield). ^{1}H NMR (500 MHz, DMSO-d_6) δ 7.22 (s, 1H), 6.86 (s, 1H), 4.33 (q, *J* = 7.1 Hz, 2H), 3.93 (s, 3H), 3.81 (s, 3H), 2.29 (s, 3H), 2.13 (s, 3H), 1.33 (t, *J* = 7.1 Hz, 3H). ^{13}C NMR (126 MHz, DMSO-d_6) δ 160.54, 159.21, 152.38, 140.05, 134.36, 128.59, 117.24, 114.43, 95.77, 59.75, 55.59, 55.36, 14.33, 13.59, 8.54.

Ethyl 1-(4,5-Dichloro-2-methoxyphenyl)-5-methyl-1*H*-1,2,3-triazole-4-carboxylate (**9d**). Compound **9d** was synthesized by using a procedure similar to that described for compound **9a**, employing 4,5-dichloro-2-

methoxyaniline and ethyl 2-diazo-3-oxobutanoate, to give a white solid (411 mg, 36% yield). ^{1}H NMR (500 MHz, DMSO-*d*$_6$) δ 7.93 (s, 1H), 7.69 (s, 1H), 4.35 (q, *J* = 7.1 Hz, 2H), 3.85 (s, 3H), 2.34 (s, 3H), 1.33 (t, *J* = 7.1 Hz, 3H). ^{13}C NMR (126 MHz, DMSO-*d*$_6$) δ 160.31, 152.67, 140.39, 134.63, 134.17, 129.21, 122.45, 121.99, 114.76, 59.92, 56.54, 13.57, 8.45.

(*S*)-*N*-(5-(*tert*-Butyl)-2-(hexan-3-yloxy)phenyl)-5-methyl-1-(2,4,5-trimethoxyphenyl)-1*H*-1,2,3-triazole-4-carboxamide (**SJPYT-326**). To a solution of ethyl 5-methyl-1-(2,4,5-trimethoxyphenyl)-1*H*-1,2,3-triazole-4-carboxylate (**9a,** 210 mg, 0.654 mmol) in water (3 mL)/DMSO (3 mL) was added lithium hydroxide (78 mg, 3.27 mmol). The mixture was stirred at room temperature for overnight. The reaction mixture was diluted by NH_4Cl (1 N, 50 mL) and extracted with EA (2 × 50 mL). The combined organic layer was washed with brine (50 mL), dried over MgSO4 and filtered. The filtrate was concentrated in vacuum, and the residue was used without purification for next step (grey solid, 136.2 mg).

In a 50 mL round-bottom flask, 5-methyl-1-(2,4,5-trimethoxyphenyl)-1*H*-1,2,3-triazole-4-carboxylic acid (**10a**, 100 mg, 0.341 mmol) was dissolved in DMF (5 mL). (*S*)-5-(*tert*-butyl)-2-(hexan-3-yloxy)aniline (102 mg, 0.409 mmol), EDCI (98 mg, 0.511 mmol), HOBt (wetted with not less than 20% by weight of water, 69.1 mg, 0.409 mmol) and DIEA (122 µl, 0.682 mmol) were added to the reaction mixture. The mixture was stirred at room temperature for overnight. Then EA (50 mL) was added to the reaction mixture and washed by water and brine. The organic phase was concentrated, and the residue was purified by flash chromatography (0−100% EtOAc in hexane) to give compound **SJPYT-326** as a white solid (129.7 mg, 72% yield, 100% purity). ^{1}H NMR (500 MHz, DMSO-*d*$_6$) δ 9.70 (s, 1H), 8.50 (d, *J* = 2.3 Hz, 1H), 7.15 (s, 1H), 7.08 (dd, *J* = 8.6, 2.4 Hz, 1H), 7.03 (d, *J* = 8.7 Hz, 1H), 6.96 (s, 1H), 4.40 (p, *J* = 5.8 Hz, 1H), 3.92 (s, 3H), 3.80 (s, 3H), 3.74 (s, 3H), 2.39 (s, 3H), 1.68 (dtd, *J* = 16.4, 13.7, 7.5 Hz, 4H), 1.51 – 1.36 (m, 2H), 1.29 (s, 9H), 0.95 (t, *J* = 7.4 Hz, 3H), 0.91 (t, *J* = 7.3 Hz, 3H). ^{13}C NMR (126 MHz, DMSO-*d*$_6$) δ 157.84, 150.82, 147.61, 143.90, 142.23, 141.95, 138.44, 136.59, 126.95, 119.80, 115.53, 113.84, 112.14, 111.46, 97.75, 78.67, 55.97, 55.73, 55.49, 34.37, 33.45, 30.74, 25.38, 17.38, 13.46, 8.50, 8.17. ESI-TOF HRMS: *m*/*z* 525.3069 ($C_{29}H_{40}N_4O_5$ + H^+ requires 525.3071).

(*S*)-*N*-(5-(*tert*-Butyl)-2-(hexan-3-yloxy)phenyl)-1-(4-fluoro-2,5-dimethoxyphenyl)-5-methyl-1*H*-1,2,3-triazole-4-carboxamide (**SJPYT-327**). Compound **SJPYT-327** was synthesized by using a procedure similar to that described for compound **SJPYT-326**, employing ethyl 1-(4-fluoro-2,5-dimethoxyphenyl)-5-methyl-1*H*-1,2,3-triazole-4-carboxylate (**9b**) and (*S*)-5-(*tert*-butyl)-2-(hexan-3-yloxy)aniline, to give a white solid (143.6 mg, two steps 57% yield, 100% purity). ^{1}H NMR (500 MHz, DMSO-*d*$_6$) δ 9.70 (s, 1H), 8.49 (d, *J* = 2.3 Hz, 1H), 7.47 (d, *J* = 8.9 Hz, 1H), 7.41 (d, *J* = 12.9 Hz, 1H), 7.08 (dd, *J* = 8.7, 2.4 Hz, 1H), 7.03 (d, *J* = 8.7 Hz, 1H), 4.40 (p, *J* = 5.8 Hz, 1H), 3.83 (s, 3H), 3.77 (s, 3H), 2.41 (s, 3H), 1.75 – 1.59 (m, 4H), 1.50 – 1.36 (m, 2H), 1.29 (s, 9H), 0.95 (t, *J* = 7.4 Hz, 3H), 0.90 (t, *J* = 7.3 Hz, 3H). ^{13}C NMR (126 MHz, DMSO-*d*$_6$) δ 157.72, 153.34, 151.36, 147.46, 147.39, 143.94, 142.24, 140.37, 140.28, 138.52, 136.72, 126.91, 119.88, 118.08, 118.06, 115.58, 113.90, 113.87, 112.15, 102.08, 101.89, 78.68, 56.25, 34.37, 33.46, 30.73, 25.38, 17.38, 13.46, 8.49, 8.11. ESI-TOF HRMS: *m*/*z* 513.2876 ($C_{28}H_{37}FN_4O_4$ + H^+ requires 513.2872).

(*S*)-*N*-(5-(*tert*-Butyl)-2-(hexan-3-yloxy)phenyl)-1-(2,4-dimethoxy-5-methylphenyl)-5-methyl-1*H*-1,2,3-triazole-4-carboxamide (**SJPYT-328**). Compound **SJPYT-328** was synthesized by using a procedure similar to that described for compound **SJPYT-326**, employing ethyl 1-(2,4-dimethoxy-5-methylphenyl)-5-methyl-1*H*-1,2,3-triazole-4-carboxylate (**9c**) and (*S*)-5-(*tert*-butyl)-2-(hexan-3-yloxy)aniline, to give a white solid (104.9 mg, two steps 50% yield, 99.5% purity). ^{1}H NMR (500 MHz, DMSO-*d*$_6$) δ 9.69 (s, 1H), 8.49 (d, *J* = 2.4 Hz, 1H), 7.27 (s, 1H), 7.08 (dd, *J* = 8.6, 2.4 Hz, 1H), 7.02 (d, *J* = 8.6 Hz, 1H), 6.88 (s, 1H), 4.39 (p, *J* = 5.8 Hz, 1H), 3.94 (s, 3H), 3.83 (s, 3H), 2.38 (s, 3H), 2.14 (s, 3H), 1.76 – 1.59 (m, 4H), 1.51 – 1.36 (m, 2H), 1.28 (s, 9H), 0.95 (t, *J* = 7.4 Hz, 3H), 0.90 (t, *J* = 7.3 Hz, 3H). ^{13}C NMR (126 MHz, DMSO-*d*$_6$) δ 159.23, 157.87, 152.40, 143.93, 142.23, 138.33, 136.62, 128.63, 126.96, 119.79, 117.25, 115.56, 114.47, 112.16, 95.79, 78.73, 55.61, 55.37, 34.39, 33.45, 30.73, 25.40, 17.39, 14.35, 13.45, 8.52, 8.14. ESI-TOF HRMS: *m*/*z* 509.3141 ($C_{29}H_{40}N_4O_4$ + H^+ requires 509.3122).

(*S*)-*N*-(5-(*tert*-Butyl)-2-(hexan-3-yloxy)phenyl)-1-(4,5-dichloro-2-methoxyphenyl)-5-methyl-1*H*-1,2,3-triazole-4-carboxamide (**SJPYT-329**). Compound **SJPYT-329** was synthesized by using a procedure similar to that described for compound **SJPYT-326**, employing ethyl 1-(4,5-dichloro-2-methoxyphenyl)-5-methyl-1*H*-1,2,3-triazole-4-carboxylate (**9d**) and (*S*)-5-(*tert*-butyl)-2-(hexan-3-yloxy)aniline, to give a white solid (143.6 mg, two steps 68% yield, 100% purity). ^{1}H NMR (500 MHz, DMSO-*d*$_6$) δ 9.69 (s, 1H), 8.48 (d, *J* = 2.4 Hz, 1H), 7.98 (s, 1H), 7.71 (s, 1H), 7.09 (dd, *J* = 8.6, 2.4 Hz, 1H), 7.03 (d, *J* = 8.7 Hz, 1H), 4.40 (p, *J* = 5.8 Hz, 1H), 3.87 (s, 3H), 2.43 (s, 3H), 1.79 – 1.57 (m, 4H), 1.51 – 1.32 (m, 2H), 1.28 (s, 9H), 0.95 (t, *J* = 7.4 Hz, 3H), 0.90 (t, *J* = 7.4 Hz,

3H). ^{13}C NMR (126 MHz, DMSO-d_6) δ 157.57, 152.66, 143.98, 142.23, 138.70, 136.82, 134.20, 129.25, 126.83, 122.49, 122.01, 119.95, 115.63, 114.78, 112.15, 78.69, 56.56, 34.37, 33.45, 30.73, 25.37, 17.37, 13.45, 8.50, 8.06. ESI-TOF HRMS: *m*/*z* 533.2098 ($C_{27}H_{34}Cl_2N_4O_3 + H^+$ requires 533.2081).

Supplementary References

1. Lin, W., Li, Y., Yang, L. & Chen, T. Development of BODIPY FL VH032 as a High-Affinity and Selective von Hippel-Lindau E3 Ligase Fluorescent Probe and Its Application in a Time-Resolved Fluorescence Resonance Energy-Transfer Assay. *ACS Omega* **6**, 680-695 (2021). https://doi.org/10.1021/acsomega.0c05221
2. Hartmann, M. & Studer, A. Cyclizing radical carboiodination, carbotelluration, and carboaminoxylation of aryl amines. *Angew Chem Int Ed Engl* **53**, 8180-8183 (2014). https://doi.org/10.1002/anie.201403968
3. Gao, D. *et al.* Rational drug design of benzothiazole-based derivatives as potent signal transducer and activator of transcription 3 (STAT3) signaling pathway inhibitors. *Eur J Med Chem* **216**, 113333 (2021). https://doi.org/10.1016/j.ejmech.2021.113333
4. Hadida, S. *et al.* Discovery of N-(2,4-di-tert-butyl-5-hydroxyphenyl)-4-oxo-1,4-dihydroquinoline-3-carboxamide (VX-770, ivacaftor), a potent and orally bioavailable CFTR potentiator. *J Med Chem* **57**, 9776-9795 (2014). https://doi.org/10.1021/jm5012808
5. Wang, Z. *et al.* Intramolecular hydrogen bonding-assisted cyclocondensation of alpha-diazoketones with various amines: a strategy for highly efficient Wolff 1,2,3-triazole synthesis. *Chem Commun (Camb)* **48**, 7076-7078 (2012). https://doi.org/10.1039/c2cc33157h
6. Kamiyama, H. and Araki, T. Iso-oxazoline compound and application thereof. JP2011098956A (2011).
7. Wang, Z. J. *et al.* Design, synthesis, and fungicidal evaluation of a series of novel 5-methyl-1H-1,2,3-trizole-4-carboxyl amide and ester analogues. *Eur J Med Chem* **86**, 87-94 (2014). https://doi.org/10.1016/j.ejmech.2014.08.029
