# Supplementary material for: Chemical manipulation of an activation/inhibition switch in the nuclear receptor PXR
Source: Nat Commun. 2024 May 14;15:4054. doi: 10.1038/s41467-024-48472-1 (PMC11094003; doi:10.1038/s41467-024-48472-1)
Supplement: Supplementary file 3 — Description of additional supplementary files [file 41467_2024_48472_MOESM3_ESM.pdf]

Description of Additional Supplementary Files

File Name: Supplementary Data 1
Description: List of key reagents used in this study and their sources.

File Name: Supplementary Data 2
Description: Coordinates of MD inputs and outputs, a representative equilibration setup file ('desmond_md_job_2.msj'), and a representative production run setup file ('desmond_md_job_2.cfg').
